# A 3D magnetic tissue stretcher for remote mechanical control of embryonic stem cell differentiation

**DOI:** 10.1038/s41467-017-00543-2

**Published:** 2017-09-12

**Authors:** Vicard Du, Nathalie Luciani, Sophie Richard, Gaëtan Mary, Cyprien Gay, François Mazuel, Myriam Reffay, Philippe Menasché, Onnik Agbulut, Claire Wilhelm

**Affiliations:** 1 Laboratoire Matière et Systèmes Complexes (MSC), UMR 7057, CNRS and Université Paris Diderot, 75205 Paris Cedex 13, France; 2 Department of Cardiovascular Surgery, Hôpital Européen Georges Pompidou; Paris Cardiovascular Research Center, INSERM U970, Université Paris Descartes, Paris, 75015 France; 3 Sorbonne Universités, UPMC Univ Paris 06, Institut de Biologie Paris-Seine (IBPS), UMR CNRS 8256, Biological Adaptation and Ageing, 75005 Paris, France

## Abstract

The ability to create a 3D tissue structure from individual cells and then to stimulate it at will is a major goal for both the biophysics and regenerative medicine communities. Here we show an integrated set of magnetic techniques that meet this challenge using embryonic stem cells (ESCs). We assessed the impact of magnetic nanoparticles internalization on ESCs viability, proliferation, pluripotency and differentiation profiles. We developed magnetic attractors capable of aggregating the cells remotely into a 3D embryoid body. This magnetic approach to embryoid body formation has no discernible impact on ESC differentiation pathways, as compared to the hanging drop method. It is also the base of the final magnetic device, composed of opposing magnetic attractors in order to form embryoid bodies in situ, then stretch them, and mechanically stimulate them at will. These stretched and cyclic purely mechanical stimulations were sufficient to drive ESCs differentiation towards the mesodermal cardiac pathway.

## Abstract (web summary)

The development of embryoid bodies that are responsive to external stimuli is of great interest in tissue engineering. Here, the authors culture embryonic stem cells with magnetic nanoparticles and show that the presence of magnetic fields could affect their aggregation and differentiation.

## Introduction

Research in regenerative medicine has advanced rapidly over the past decade thanks to the development of multiple tools (e.g., 3D printing and 3D culture, controlled forces and microenvironments, cell differentiation and reprogramming)^1–4^. Stem cells and their unique potential for differentiation lie at the heart of this emerging field.

In particular, a growing number of studies have evidenced that mechanical factors can influence stem cell differentiation^5^. This idea of a physical guidance of differentiation emerged from studies using adult mesenchymal stem cells, and was then tested on pluripotent/embryonic stem cells. Most techniques applied on two-dimensional (2D) cell cultures, focusing in particular on the role of microenvironmental mechanical cues such as substrate rigidity^6–11^, flow-induced shear stress^12–14^, strains imposed on cell monolayers by the stretching of deformable supporting membranes^15–17^, or local forces applied on beads attached to the cell surface^18, 19^.

Multicellular three-dimensional (3D) approaches have also received an increasing interest for studying stem cell behavior beyond the classical 2D culture conditions. First, scaffold-based constructions not only allow to stimulate mechanically the seeded stem cells^20, 21^, but also provide precise 3D control of extracellular matrix cues^22, 23^. Second, scaffold-free magnetic or printing technologies make it possible to control spatial patterning of aggregates^24^ or to create multilayer structures^25^.

One current challenge is now to provide other methodologies to assemble and organize stem cells (only) into a 3D tissue structure that can be stimulated at will, in order to explore the physical differentiation approaches in 3D purely cellular tissues.

To create a 3D cell assembly, one needs to enable remote spatial organization of component cells. Magnetic cellular forces acting at a distance are appealing candidates for this application, provided the individual cells are first magnetized by the internalization of magnetic nanoparticles. Magnetic nanoparticles in regenerative medicine are mostly used either for noninvasive in vivo tracking of stem cells by magnetic resonance imaging^26–29^, or for magnetic cell targeting to sites of tissue damage^21, 30–32^. The idea of using magnetic cell manipulation for tissue engineering is more recent, and the first works featured bioprinting and cell sheet engineering, by magnetically creating or manipulating spheroids^33–35^ or organizing layers of several cell types^36, 37^, respectively. To use magnetic forces not only to form tissues, but also to remotely stimulate them, is still to be unraveled.

Incorporating nanoparticles to magnetize and stimulate cells raises several issues. The first is the impact of nanoparticle internalization on the cell phenotype, and particularly differentiation capacity. Previous studies^31, 38^ of mesenchymal stem cells have shown that magnetic nanoparticles generally do not inhibit their differentiation, except for chondrogenesis in some cases^39^, in particular at high iron doses^40^. Besides, magnetic nanoparticles can also be beneficial to mesenchymal stem cells differentiation, e.g., for myocardial repair^41, 42^. Only few studies have investigated the impact of magnetic nanoparticles on embryonic stem cells (ESCs). One reported that cardiomyogenesis was unaffected^43^, another that the self-renewal ability or surface phenotypic markers expressed after forced differentiation into hematopoietic cells were unchanged^44^. To the best of our knowledge, the impact of magnetic nanoparticles on the whole ESC differentiation profile, with no biochemical triggers, is still unknown.

ESC differentiation is initiated within an embryoid body (EB), generally created with the hanging drop method. A second important question is thus whether 3D magnetic printing of ESCs could be equivalent to this method and what would be its impact on the differentiation profile after cell maturation. The ultimate and most challenging question is whether magnetic forces alone could drive stem cells differentiation within a magnetically formed 3D model tissue.

**Fig. 1:**
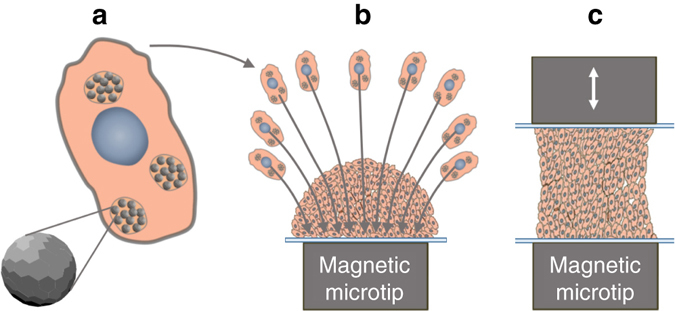
Schematic illustrating the different steps involved in the magnetic stretcher. **a** Nanoparticles incorporation in ESCs, **b** EBs formation from magnetized ESCs driven by a magnetic microtip, and **c** EBs magnetic stimulation in situ, in the 3D geometry, and without the need for a supporting matrix

Here we address all three issues by using magnetized ESCs to create an EB and remote magnetic forces to stimulate it (Fig. 1). We first carefully analyzed iron oxide nanoparticle internalization by ESCs, and its impact on their viability, pluripotency and differentiation. Second a magnetic attraction method was developed to create EBs, and its impact on the ESC differentiation profile was evaluated. We then designed an all-in-one magnetic stretcher capable of both creating and stimulating the EB and evidenced the impact of purely mechanical stimulation on EB differentiation.

## Results

### ESCs magnetic labeling, viability and pluripotency

**Fig. 2:**
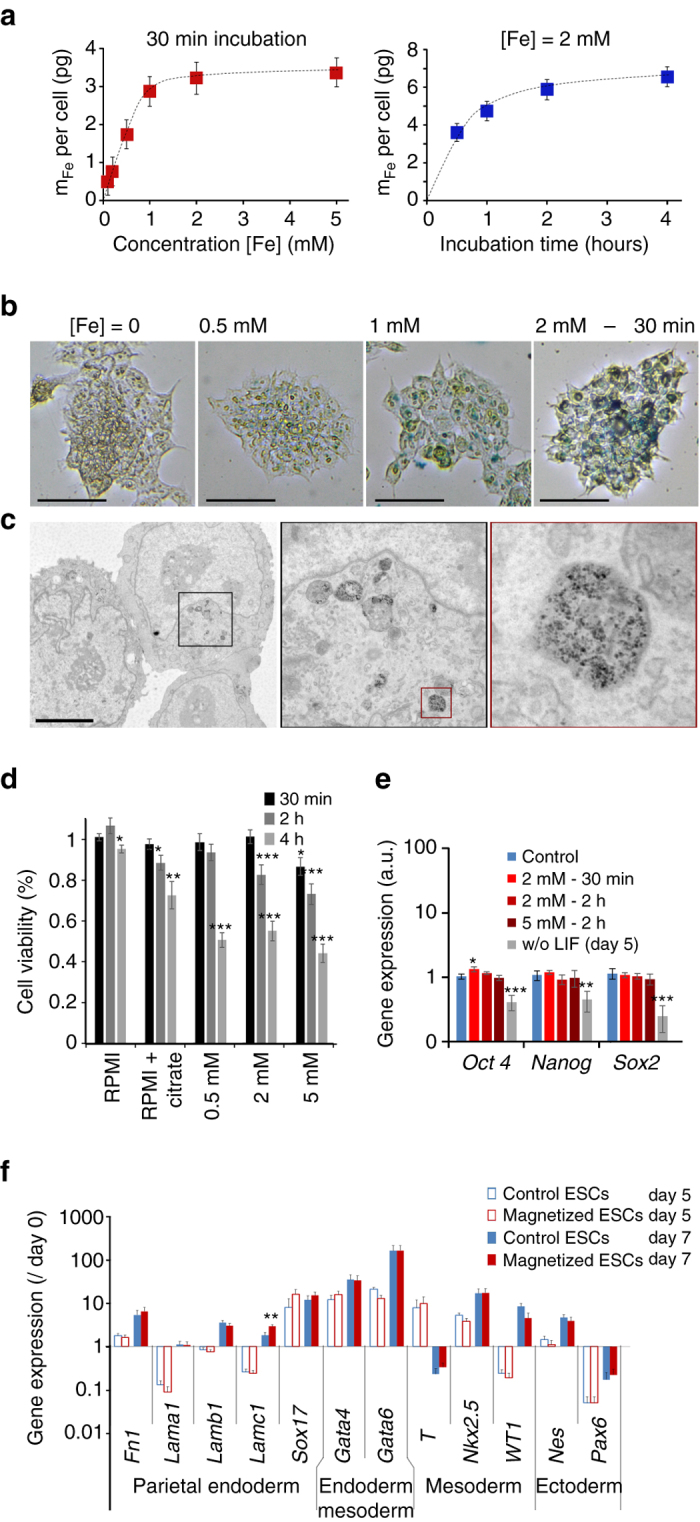
Optimization of embryonic stem cell (ESC) magnetic labeling. **a** Magnetic labeling of ESCs at different extracellular iron concentrations (for a fixed incubation time of 30 min) and during different incubation periods (for a fixed iron concentration of [Fe] = 2 mM). **b** Perls’ Prussian bl﻿ue staining of ESCs after labeling with different concentrations of extracellular iron (between 0.5 mM and 2 mM), and a fixed incubation time of 30 min. *Scale bar*: 250 µm. **c** Transmission electron micrograph of ESC after labeling for 30 min at [Fe] = 2 mM (successive zooms of framed areas). Scale bar: 5 µm. Nanoparticles are all located inside the lysosomes. **d** Cell viability testing using Alamar Blue detection of cell metabolic activity. Cell viability was calculated relative to the control (unlabeled cells in complete medium) and was measured 2 h after different incubation periods (in RPMI) with different extracellular iron concentrations and incubation times. **e** Expression of pluripotency genes *Oct4*, *Nanog* and *Sox2* measured by real-time PCR. The gene expression level was calculated with respect to *RPLP0* mRNA and expressed as compared to control (unlabeled cells, cultured in complete medium with LIF, = 1 ± SEM). A positive control was added in which the LIF has been removed during 5 days before analysis (culture in complete medium without LIF). One can note that only one condition led to a significant upregulation (*Oct4*—incubation at 2 mM for 30 min). However the gene was upregulated <1.5-fold (1.3-fold exactly). Besides, higher doses (2 h incubation at 2 and 5 mM) provide the same *Oct4* expression as the control. **f** Expression of several genes characteristic of the different embryonic layers in hanging drop EB formation conditions with 1000 unlabeled (control, *blue bars*) or labeled cells (magnetic, *red bars*), 5 days (*open bars*) and 7 days (*solid bars*) after initiation of differentiation. All values were calculated with respect to *RPLP0* mRNA and normalized by the expression value of the same gene measured at day 0. Two-sample *t*-test was used to compare the control group with the magnetic group, for same gene and same day; *p < 0.05; **p < 0.01; ***p < 0.001. All *error bars* represent the SEM

The first step was to incorporate magnetic nanoparticles within ESCs. Magnetophoretic measurements^40^ of the iron load per cell (Fig. 2a) yielded uptake curves (in terms of pg of iron per cell) that saturated as a function of both the extracellular iron concentration [Fe] and the incubation time. Thus, by setting the incubation time to 30 min, a saturation value of 3.3 ± 0.5 pg was obtained with [Fe] = 2 mM. When the concentration was set at [Fe] = 2 mM, a saturation value of 6.6 ± 0.5 pg was obtained after 2–4 h. Nanoparticle uptake can be directly imaged with Perls’ Prussian blue staining (Fig. 2b and Supplementary Fig. 1 for other views). Observation of cell colonies showed that the stronger the labeling, the more intense the blue color, up to saturation, correlating well with the magnetophoretic measurements. Finally, transmission electron microscopy of labeled cells showed that the nanoparticles were all internalized and localized within lysosomes; no nanoparticles were observed outside the cells (see Fig. 2c and Supplementary Fig. 2 for other views).

Because ESCs are particularly sensitive to perturbations, the possibility that nanoparticles might impact their viability and proliferation had to be first considered. To test the viability, ESCs metabolic activity was measured after magnetic labeling in different conditions (iron masses between 1.8 and 6.6 pg per cell). It is important to note that the cells must be incubated in RPMI medium with 5 mM citrate to prevent nanoparticle aggregation before cell internalization. It is therefore also necessary to quantify the impact of citrate itself on cell viability. Figure 2d shows cell viability for all the conditions tested, as compared to the control (unlabeled cells in culture medium). The short labeling period of 30 min had no impact at 0.5 or 2 mM extracellular iron, but a slight effect (about −10%) was noted at 5 mM iron. Upon increasing the labeling period, viability fell at all iron concentrations. However, the viability of the cells incubated with citrate alone was modified in the same way as during magnetic labeling. Citrate alone thus impacted cell viability, whereas the nanoparticles had only a slight impact on viability at high iron concentrations. Cell viability was also examined on the long-term, over 9 days after labeling for the 30 min incubation condition (3 pg per cell), and no impact was observed on the cells’ viability and ability to replicate compared to control cells (Supplementary Fig. 3).

The next step was to analyze the impact of magnetic labeling on ESC pluripotency. The first indicator of ESC pluripotency is their morphology; undifferentiated cells form round colonies. As shown in Supplementary Fig. 4, ESC colonies remained well-rounded after labeling under different conditions. Figure 2e shows that the expression level of the key pluripotency genes *Oct 4*, *Nanog* and *Sox2* were unaffected by the internalization of the nanoparticles, whatever the labeling condition, as compared to the control (unlabeled cells). By contrast, the positive control (culture without the leukemia inhibitor factor LIF, essential to maintain pluripotency) suffered a significant decrease of these genes at day 5. In view of these different results, the labeling condition of 30 min at [Fe] = 2 mM was chosen for subsequent experiments, as it yielded an iron mass of 3 pg per cell without affecting viability, proliferation, morphology and pluripotency. Hereafter, the resulting ESCs are referred to as magnetized ESCs.

### Impact of magnetic labeling on differentiation profile

The next mandatory step was to retrieve the differentiation profile of EBs formed with magnetized ESCs, and compare it with the one of control EBs (unlabeled ESCs). We chose the standard hanging drop EB formation method to initiate differentiation. This method begins with the seeding of 1000 ESCs within a 30 µl drop, which spontaneously assembles into an EB. Supplementary Fig. 5 shows images of EB formation with unlabeled control and magnetized ESCs. No difference in EB morphology was observed. mRNA from control EBs and magnetized EBs were then collected and analyzed on days 5 and 7. The expression levels (shown in Fig. 2f) of *Fn1*, *Lama1*, *Lamb1*, *Lamc1*, *Sox17* (endoderm markers); *T*, *Nkx2.5*, *Wt1* (mesoderm markers), *Gata4*, *Gata6* (meso-endoderm markers); and *Nes* and *Pax 6* (ectoderm markers) were measured by real-time PCR. Importantly, the obtained results showed no differences between control and magnetized EBs, demonstrating that the temporal expression pattern of the different genes was very similar.

### Magnetic formation of EBs

**Fig. 3:**
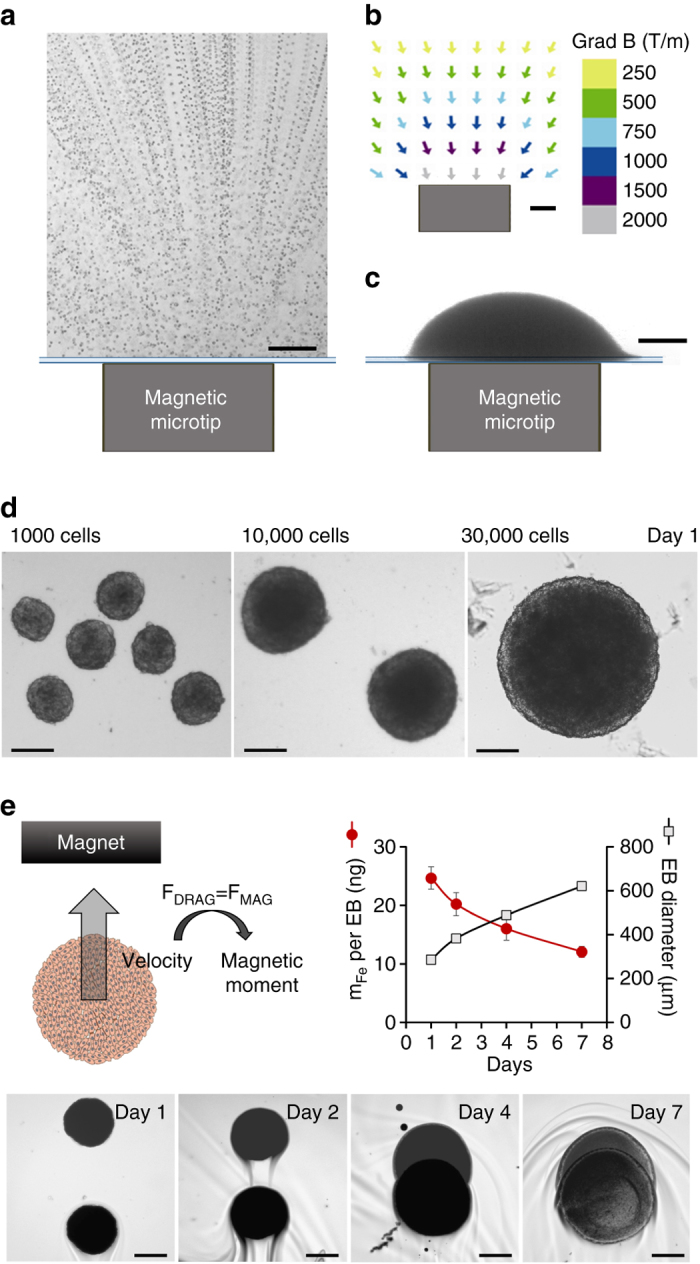
Magnetic formation of embryoid bodies. **a** ESC attraction by a magnetic microtip (750 µm in diameter). To visualize cell movement, the microtip was introduced into a chamber containing suspended cells under a microscope, and cell movements were video-monitored with a ×10 objective. Here 100 movie images were superimposed (0.1 s time intervals) in order to directly observe the trajectories of the cells migrating towards the magnetic microtip. At 1 mm from the microtip, the cells migrate at an average velocity of 300 µm/s, which corresponds to an iron mass of 3 pg cell in a magnetic field gradient of 300 mT/mm. **b** The field gradient was mapped around the microtip by studying the migration of monodisperse magnetic beads with a calibrated diameter of 4.6 µm (Dynal). At 1 mm from the microtip, it was 300 mT/mm. *Scale bar*: 200 µm. **c** Final image of the aggregate obtained 1 min after seeding 30 000 ESCs over the magnetic microtip. *Scale bar*: 200 µm. **d** Microscopic images of embryoid bodies (EBs) on day 1, obtained by seeding 1000, 10,000 and 30,000 cells per microtip. *Scale bar*: 200 µm. **e** Monitoring of EBs magnetism over 7 days after nanoparticles cellular incorporation, and EB formation (day 0). It consists of tracking the EB magnetic migration towards a magnet, and measuring the corresponding velocity, which translates into the EB magnetic moment (proportional to the mass of iron per EB) by balancing the viscous drag and the magnetic force. Typical migrations are shown for the different times (days 1, 2, 4 and 7), corresponding to the superimposition of two images at 3 s interval. *Scale bar*: 200 µm. The mass of iron (*circles*) and the EBs diameters (*squares*), averaged over eight different EBs, were then plotted as a function of time. *Error bars* represent the SEM

Having established the optimal conditions for ESC magnetic labeling, the next challenge was to create EBs magnetically, as an alternate method to hanging drop or others. The idea was simple: nanoparticles contained in cells confer a cellular magnetic moment M_cell_ (measured by magnetophoresis), which allows the cells to be attracted by a magnetic force F_m_ = M_cell_ gradB. In order to use this force to confine cells within a spheroid, gradB must be at submillimeter scales. To achieve this, we developed submillimetric (750 µm diameter) metal tips to channel the magnetic lines of an external field (about 0.2 T), creating a strong field gradient localized in space. Figure 3a shows ESC attraction by one magnetic microtip, perfectly matching the gradient map (Fig. 3b). The field gradient is 500 T m^−1^ at 1 mm from the surface of the microtip (1000 T m^−1^ at 0.4 mm), equivalent to a force of about 100 pN (200 pN, respectively) on an ESC containing 3 pg of iron.

Networks of these magnetic spikes were used to assemble several EBs in the same dish (Supplementary Fig. 6 explains in detail the fabrication of these magnetic devices). The microtips were spaced a few millimeters apart in order to preserve the localization of the field gradient created by each microtip. Magnetic formation of EBs was then straightforward (Supplementary Fig. 7): on day 0, the cells were labeled, detached and placed in a Petri dish (after non-adhesive treatment with PLL-PEG), itself placed over the magnetic microtip array. We tested 1000, 10,000 and 30,000 cells per microtip (and thus per EB). Figure 3c shows EB formed from 30,000 cells, positioned over a microtip, right after deposition. Figure 3d show the spheroids thus obtained, one day after cell deposition (day 1).

### Long-term intracellular fate of the nanoparticles in the EBs

One essential question remains that of the fate of the nanoparticles once internalized within ESCs. Or alternatively, will the EB stay magnetic over long-term culture conditions? To address this issue, we monitored EBs’ magnetism (initially 10,000 cells) at different times after EB formation, by magnetophoresis (Fig. 3e). Briefly, it consists in tracking the EB magnetic mobility when submitted to a homogeneous magnetic field gradient created by a permanent magnet. The magnetic velocity can then be directly converted into the EB magnetic moment, or alternatively the amount of nanoparticles (expressed in mass of iron) contained within the EB. At day 1 after formation, each EB contains on average 25 ng of iron, consistent with the initial iron load per single ESC of about 3 pg. This amount progressively decreases during EB growth, reaching about half its initial value at day 7. This is due to the lysosomal degradation of the nanoparticles, as recently evidenced in MSC spheroids^45, 46^. While the degradation is beneficial for long-term ability of magnetically-labeled tissue to get rid of the initial nanoparticles, the fact that at day 7, EBs still retain half their magnetization is also beneficial for multiple magnetic stimulations before tissue maturation.

### Magnetic EB formation versus hanging drop

**Fig. 4:**
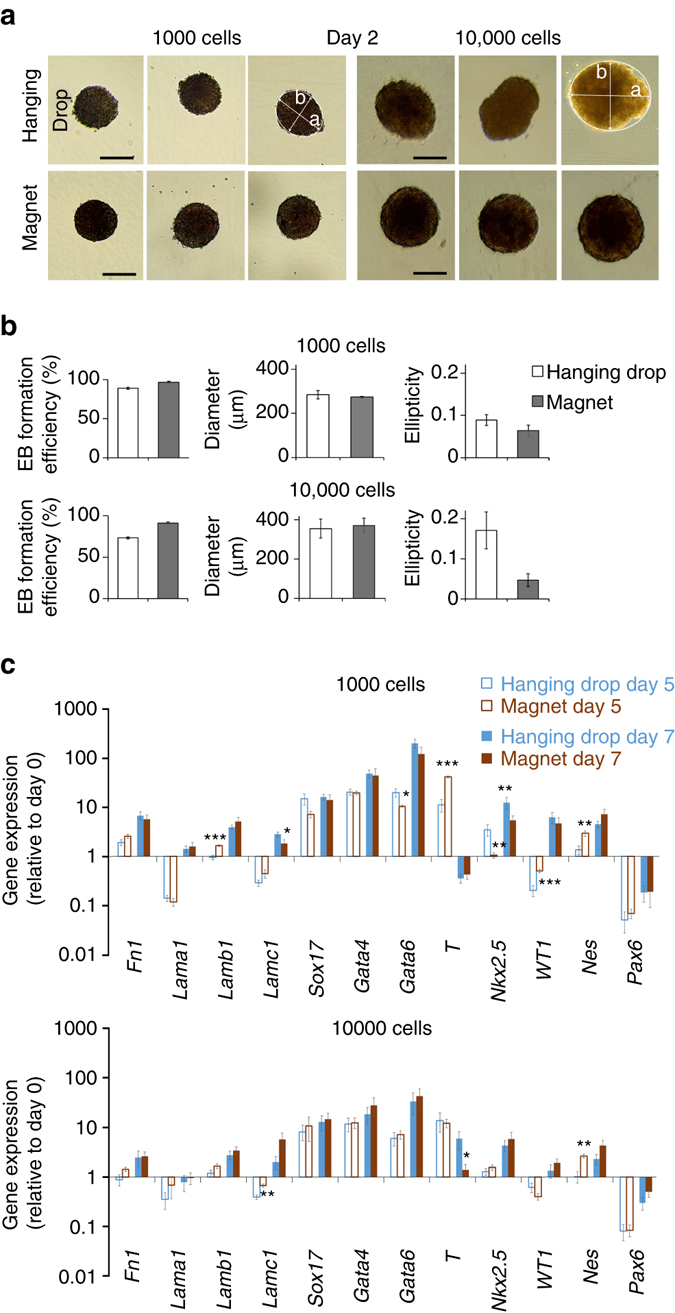
Comparison between magnetic EB formation and hanging drop method. **a** Typical images of EBs observed at day 2 after seeding (of 1000 or 10,000 ESCs), either in hanging drop or over a magnetic attractor. The short axis b and the long-axis a of the equivalent ellipse were determined by image analysis (Image J). *Scale bar*: 200 µm. **b** Quantification over 50 EBs: Efficiency is calculated as the number of EBs actually formed over the number of hanging drops deposited or of magnetic attractors present below the dish; the diameter (expressed in µm) is the effective diameter computed from the EBs areas; and the ellipticity is defined as 1-b/a. **c** Expression of a panel of genes characteristic of the different embryonic cell layers in EB obtained with hanging drop or magnetic aggregation with either 1000 or 10,000 cells. All gene expressions were normalized using the reference gene *RPLP0* mRNA, and calculated relatively to the expression of the same gene obtained at day 0, prior to EB formation. The values varied very little from one condition to another. Even statistically significant differences (over- or under-expression) were small: with 1000 cells on day 5, *Lamb1* expression increased by a factor of 1.7 and *T* expression by 3.7, while *Nkx2.5* fell by a factor of 3.2, *Wt1* rose by 2.5 and Nes rose by 2.1. On day 7, *Lamc1* expression fell 1.5-fold and *Nkx2.5* fell 2.3-fold. With 10,000 cells, *Lamc1* increased 1.7-fold and *Nes* 2.6-fold on day 5, while *T* fell 4.2-fold on day 7. All *error bars* represent the SEM

This system of magnetic formation allows tight control of EB size, contrary to the hanging drop method, which yields EBs of more variable size and, in some cases, no EBs at all. Figure 4a shows the percent of EB successfully formed, the EB average diameter and ellipticity, for magnetic EB formation or hanging drop, starting from 1000 or 10,000 ESCs. Magnetic EB formation appears particularly advantageous when starting from 10,000 cells, where the success rate of formation increases from 73 to 91% when using magnetic formation instead of the hanging drop approach, and EB ellipticity decreases from 0.17 to 0.04. The size control is also increased as demonstrated by a thinner distribution of EBs sectional areas in case of magnetic formation (see Supplementary Fig. 8). Finally, magnetic EB formation is almost instantaneous, and the transfer step (drops to dishes on day 2) necessary for the hanging drop method is avoided.

We then studied ESC differentiation under these magnetic conditions of EB formation. As previously, differentiation is initiated at day 0 of magnetic EBs formation by LIF removal from culture medium. Figure 4b compares the magnetic formation technique (magnet condition) to the hanging drop method, for 1000 and 10,000 cells deposited per EB, on day 5 and day 7 of maturation. The expression level of most of the genes involved in the three embryonic layers of the EBs formed magnetically was unchanged compared to control EBs formed with the hanging drop. Significant differences were observed in only 10 of the 48 conditions, and significant differences observed for one gene are never spread to all conditions (from day 5 to day 7, or from 1000 cells to 10,000 cells).

### Magnetic stretcher for in situ EB formation and stimulation

**Fig. 5:**
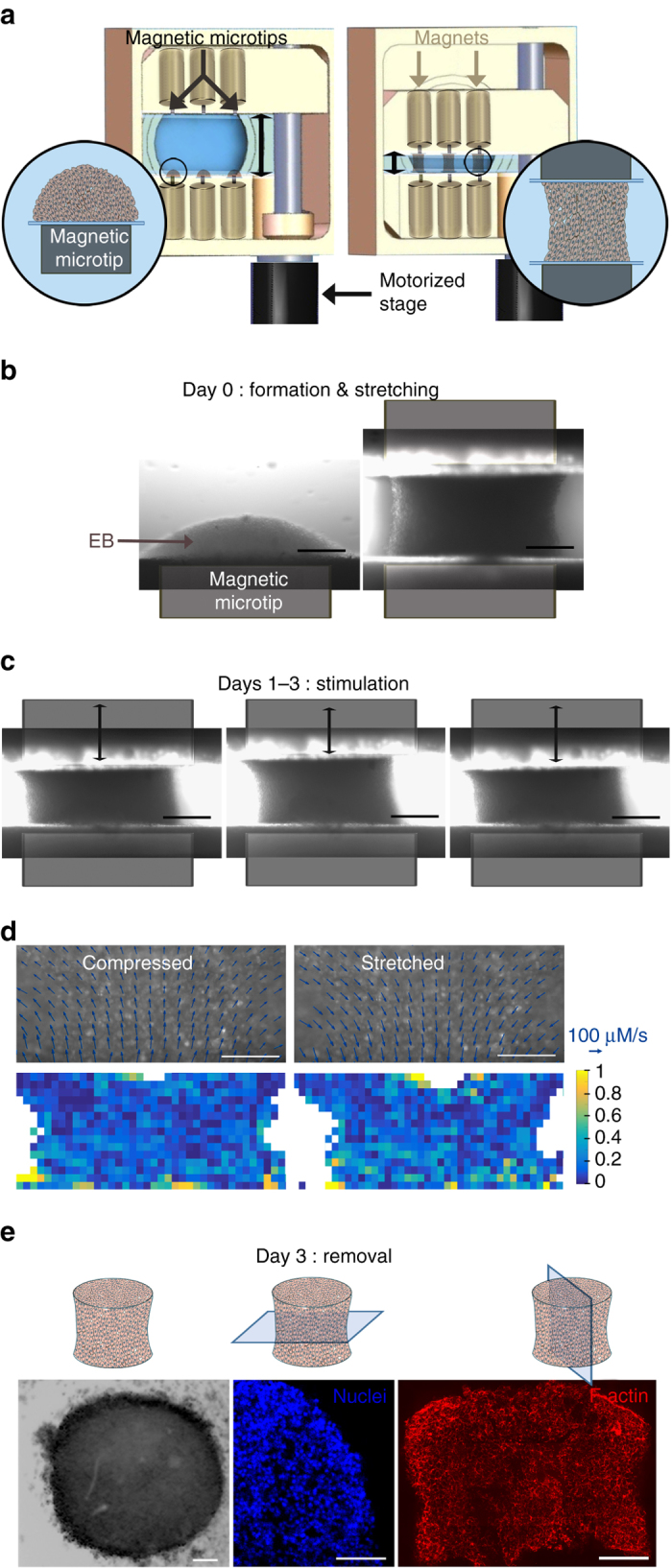
Magnetic stretcher: formation and stimulation of EBs. **a** Diagram of the magnetic stretcher device developed. Three EBs could be created on magnetic microtips (magnetized by permanent magnets), and then could be stretched/stimulated by approaching another 3 microtips (also magnetized by permanent magnets). The system is motorized to realize micrometer displacement of the second mobile magnetic microtips system. **b** Typical images of the first phases on day 0: EB formation and stretching. *Scale bar*: 200 µm. **c** Typical images of the cyclic stimulation (here at days 1-3). Scale bar: 200 µm. **d** Fluorescence images of membrane-stained cells in compressed and stretched EBs (10% imposed strain) are overlaid with velocity vectors extracted from PIV analysis (*arrow bar* scales for a speed of 100 µm/s). Only one fourth of the vectors are represented for easy reading. *Scale bar*: 100 µm. The divergence of the velocity field (for stretching) or its opposite (for compression) representative for the strain rate is mapped in both cases. For compression and stretching steps the mean effective strain rate sensed by cells is calculated at 0.32 ± 0.08 and 0.32 ± 0.06 s^−1^, respectively. **e** EB sampling on day 3 (here shown for a “cyclic” condition): Optical microscopy right after magnet removal and fluorescent imaging (DAPI staining, middle; F-actin staining, right) of 16-µm cryosections in the perpendicular and parallel direction of the tissue axis. The nuclei image shows a homogeneous cell density in the center of the EB, while F-actin is homogenous whatever the localization of the cell inside the stretched EB. *Scale bar*: 100 µm. All EBs were formed with 10,000 ESCs

ESCs thus retain a similar differentiation profile as obtained in control condition, not only after incorporating magnetic nanoparticles, but also after magnetic formation of EBs. The concept of the magnetic stretcher then becomes relevant: the EB should be formed in the culture medium by the same magnetic microtip as described above, then another magnetic microtip would be approached to trap and deform (stretch/stimulate) it (Fig. 5a), and only this mechanical stimulation step would be responsible for any change in differentiation capacity.

The magnetic stretcher (Fig. 5a and Supplementary Fig. 9) combines fixed and mobile microtips for stretching and stimulation, in a perfectly sterile setup. With dimensions (20 × 40 × 20 cm), it fits easily inside an incubator, to maintain the EBs temperature at 37 °C, with 5% CO_2_. It was also designed to be placed directly on a microscope stage, thermalized at 37 °C, without compromising sterility. The magnetic microtips are inserted in machined structures closed by glass slides 100 μm thick to prevent contact between the metal and the culture medium, while allowing them to be very close to the cells. Removable cylindrical magnets are used to magnetize the microtips. The device allows three EBs to be formed and stimulated at the same time, and medium reservoirs (communicating with the rest of the setup) are placed between the microtips to ensure nutrient distribution to the EBs. A micrometric motor (Thorlabs, controller interfaced with Labview) ensures the normal motion of one of the structures carrying the magnetic devices (microtips and magnets), while two micrometric displacements ensure alignment along the other two axes.

The EBs are initially formed from 10,000 cells on the fixed magnetic structure (Fig. 5b). These EBs correspond to the magnet condition, similar to the one shown in Fig. 3c, which were found to be identical in terms of differentiation profile to EBs formed with hanging drop and 10,000 ESCs. As the second microtip is approached, 1 h after EB formation, the EB is deformed between the two tips (Fig. 5b). This corresponds to the stretched condition, for which, during the following 3 days, each EB is maintained in its stretched configuration. Finally the cyclic condition corresponds to an additional cyclic stimulation (at a frequency of 1 Hz and an amplitude of 10%) for two 2 h periods each day of the following 3 days (day 1, day 2 and day 3; Fig. 5c).

In this setup, the magnitude of the magnetic (intracellular) force applied to single ESCs within the magnetic EB needs first to be quantified. At 400 µm from the magnetic tip/attractor and for ESCs loaded with 3 pg of iron (or equivalently a magnetic moment of 2 × 10^−13^ A m^2^), the magnetic gradient of about 1000 T/m provides a force of approximately 200 pN per single ESC. More precisely, the magnetic force is exerted on the nanoparticles clustered within lysosomes. Each lysosome is thus submitted to a force of 1 pN since it contains a maximum of 10^4^ nanoparticles and bears a magnetic moment in the range of 10^−15^ A m^2^. Lysosomes, which are embedded in the viscoelastic cytoplasm, are observed not to agglomerate onto each other or drift significantly within the cell, see Supplementary Fig. 10 which shows that the intracellular pattern of the magnetic lysosomes is the same with or without magnet application.

To document the stimulation at single-cell levels, cell movements were monitored over several stretching cycles using a membrane cell marker (Pkh26). PIV analysis provided the velocity field of the cells submitted to stretching and compression due to magnet displacements (Fig. 5d). No shear zones are noticeable on this figure. Moreover cells inside the EBs are submitted to a uniform strain over the whole aggregate. Indeed the divergence of the velocity field which is representative for the strain rate^47, 48^ is homogenous. The average effective strain rate is 0.32 ± 0.08 s^−1^ for the stretching step and 0.32 ± 0.06 s^−1^ for the compression step. Thus all cells experience essentially the same deformation rate.

Finally, in all conditions, at the end of day 3, the spheroids could be released (Fig. 5e) by removing the permanent magnets (thus canceling the magnetic force), and transferred into dishes to mature until day 5 before mRNA collection and analysis. The fact that each EB was easily released as soon as the microtips were demagnetized shows that the stretching is due to remote forces, exerted at the heart of the tissue structure, with no direct contact. Besides, as demonstrated in Fig. 5e on cryosections parallel and perpendicular to the tissue axis, the EB maintains its engineered shape.

**Fig. 6:**
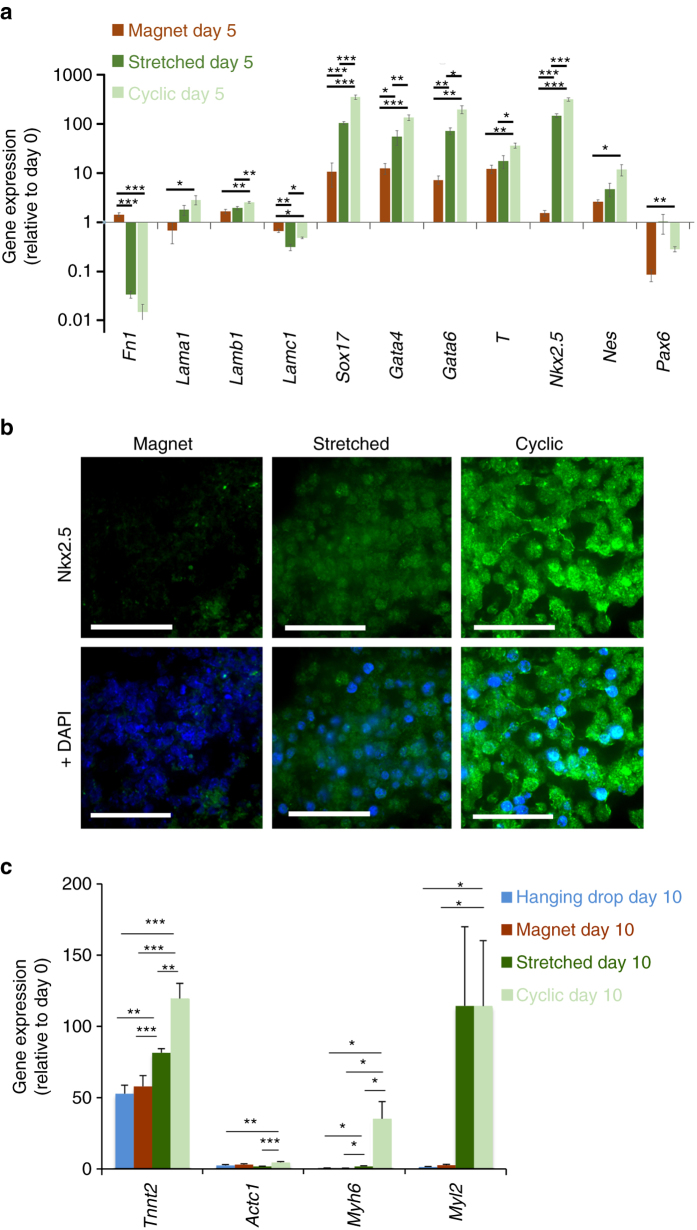
EBs characterization for the magnet, stretched and cyclic conditions. **a** Expression of genes characteristic of the different embryonic cell layers in EB after 5 days maturation (day 5). All EBs were obtained from 10,000 magnetized ESCs. Magnet: EB created on a magnetic microtip; Stretching: EB formed on a magnetic microtip, then stretched between two microtips; Cyclic: as before, plus stimulation at 1 Hz twice a day for 3 days. Gene expression (normalized to *RPLP0*) is calculated relative to the same gene expression at day 0 before EB formation. **b** Immunostaining (in green) of Nkx2.5 for EBs in the three conditions, with DAPI staining overlaid on the right. Images are obtained at the center of each EB. *Scale bar*: 50 µm. **c** Gene expression at longer maturation times (day 10) for specific cardiac markers cardiac troponin T (*Tnnt2*), cardiac α-actin (*Actc1*), α myosin heavy chain (*Myh6*) and myosin regulatory light chain 2 (*Myl2*). All EBs were obtained from 10,000 ESCs. For the hanging drop formation (blue), ESCs were not labeled with the magnetic nanoparticles. For the three other conditions, ESCs were magnetic (3 pg of iron per cell): EB formation by magnet with no further stimulation (dark red), stretched stimulation (dark green) and cyclic stimulation (light green). mRNA levels are shown relative to control (day 0, defined as 1), and normalized to reference gene RPLP0. *Error bars* represent the SEM

Figure 6a shows the expression levels of genes characteristic of the different embryonic layers, under the three conditions (magnet, stretched and cyclic), and at day 5. All levels are expressed relatively to the same genes expression at day 0, right before EB formation and LIF removal. The expression levels of the stretched and cyclic conditions must thus be compared with the ones of the magnet condition, which are almost identical to the control hanging drop method. First, we can note an increase in the expression of *Nkx2.5* involved in the cardiac mesoderm pathway for the stretched condition, enhanced for the cyclic condition. Concerning the other mesoderm gene *T*, because the decrease in the expression of this gene has already begun at day 5 (see Supplementary Fig. 11 for the timing of *T* and *Nkx2.5* involvement in cardiac differentiation), upregulation is lesser, nevertheless increased for the stretched and cyclic conditions. Second, we also measured a significant increase of the 3 genes involved in the next stage towards cardiac differentiation, *Sox17*, *Gata4* and *Gata6* (Supplementary Fig. 11 also summarizes the role of these genes in cardiac differentiation), compared to the control magnet condition, and this increase was higher for the cyclic condition. Finally, and logically, the expressions of other genes involved in the endoderm or ectoderm pathways, were either lowly upregulated, with levels close to the ones for undifferentiated cells (*Lama1*, *Lamb1*, *Lamc1*, *Nes* and *Pax6*) or down-regulated (*Lamc1* and *Fn1*).

Immunostaining of the Nkx2.5 protein confirmed these results (Fig. 6b). Its detection increased markedly in the stretched condition, and even more strongly after cyclic stimulation.

Finally, in order to detect if a commitment towards the cardiac lineage was really enhanced, we analyzed EB at longer maturation times (day 10, Fig. 6c), and quantified by qPCR the expression of transcripts encoding for specific cardiomyocyte markers. We tested troponin T (*Tnnt2*), involved in cardiomyocyte contraction, cardiac α-actin (*Actc1*), the cardiac cytoskeletal marker, α myosin heavy chain (*Myh6*), involved in contraction and considered as a maturation marker, and myosin regulatory light chain 2 (*Myl2*), involved in the regulation of myosin ATPase activity and known as a ventricular cardiomyocyte marker. The hanging drop and magnet conditions of EBs formation led to similar results for all genes and, overall, the efficiency of differentiation towards functional cardiomyocytes was increased upon application of stretched and cyclic stimulations. Compared to the magnet condition, *Tnnt2*, *Myh-6* and *Myl2* genes were overexpressed for the stretched condition, and this upregulation was higher following cyclic stimulation for *Tnnt2* and *Myh-6*. The impact on cardiac α-actin was less pronounced, with a significant upregulation only for the cyclic condition. This protein is, among others involved in the left ventricular compaction^49^, and probably expressed later.

## Discussion

The main objective of this work was to provide a method for assembling embryonic stem cells into 3D embryonic bodies without the need for a scaffold and further stimulating mechanically this embryoid body in situ, with the overriding aim to determine whether embryonic stem cell differentiation could be enhanced in this 3D setting through mechanical stimulation. The engineering approach we propose is a magnetic one, in which magnetic forces are created intracellularly on internalized magnetic nanoparticles and used to manipulate single ESC within a 3D construct, to produce 3D embryoid bodies with an inherent capacity for further physical stimulation.

First, one must insist on the fact that, while ESCs are particularly vulnerable to external perturbations, yet, their differentiation profile after magnetic nanoparticle incorporation was remarkably preserved. This was mandatory for the next steps of EBs formation and stimulated differentiation. This result must also be put into perspective with the ongoing use of magnetic nanoparticles for regenerative medicine. Indeed, ahead of this application, the question of magnetic nanoparticles on stem cells differentiation must be addressed, and yet rarely was. Most works investigated impact on mesenchymal stem cells differentiation, with controversial results: some showed no effect on differentiation^31^ while others showed an inhibition of chondrogenetic differentiation pathway^50^. One possible cause of this inhibition seems to be the cellular iron dose, with no impact at a low dose and inhibition at higher doses^40^. Here we provide a comprehensive analysis of the impact of magnetic nanoparticles on the whole differentiation profile of embryonic stem cells, and we evidence a striking preservation of this profile, boding well for their medical applications.

Studies of ESC differentiation require the creation of 3D multicellular aggregates (embryoid bodies). There are three main conventional approaches to EB formation in vitro^51^: suspension culture with spontaneous aggregate formation; encapsulation in hydrogel; and the hanging drop method. The latter is the most widely used but it is time-consuming and requires multiple transfer steps. Control of EB size is a fundamental issue, as it plays an important role in differentiation, as demonstrated with alternative methods using microfabrication to control the size, cell number and shape of EBs^52–55^. Here we propose another alternative method for in situ EB formation on a magnetic attractor, with no impact on the differentiation profile when compared to the hanging drop technique. In addition, the equivalence between the two methods is robust, as demonstrated by the passage from 1000 cells to 10,000 cells per EB: with both the hanging drop method and the magnetic method, a fall in the expression of endoderm and mesoderm marker genes (especially the *T* gene on day 5) was observed as EB size increased, in keeping with the few studies that have examined the influence of EB size on ESC differentiation^52, 54, 56^. The magnetic method of EB formation could thus be an interesting alternative to conventional techniques, especially as it avoids most of the manipulations associated with the hanging drop method.

**Fig. 7:**
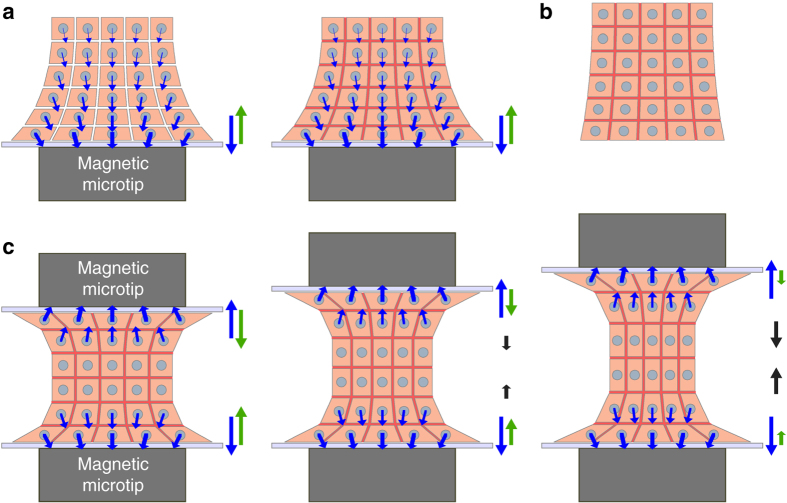
Schematic view of the forces involved within the EB in the magnetic stretcher. **a** Formation of the EB on the magnetic microtip located below a glass wall. Each cell is subjected to a magnetic force (*blue arrow*). The total resulting magnetic force (shown on the *right-hand side*, also in *blue*) is exactly balanced by the wall reaction force (*green arrow*). This pair of forces act like a “clamp” that holds mainly the “proximal” region of the sample, closest to the glass wall. After the magnetic contact, adhesion molecules (in *red*) develop the EB cohesion, without affecting forces. **b** The whole aggregate can then be used as a standalone EB. **c** When another magnetic microtip is approached with another glass wall, the upper cell layers are “clamped” against the upper wall in a similar way as in **a**. Varying the separation of both “clamps” makes it possible to adjust or cycle the (tensile) strain of the main part of the EB (represented here with a thickness of only two cells for simplicity, but actually corresponding to the major part of the entire EB)

In order to fully understand the formation and stimulation of the EB from a mechanical point of view, let us now examine the corresponding force balance in the magnetic stretcher apparatus, as depicted in Fig. 7.

During EB formation (Fig. 7a), the magnetic microtip subjects each cell to a magnetic force (blue arrow), pulling it against its neighbors, and thus contributing to squeezing all cells, but more strongly the ones closer to the microtip. The total resulting cellular magnetic force is then transmitted to the glass wall above the microtip, and is exactly balanced by the wall reaction force (green arrow). Straight after magnetic cell assembling, cohesion builds up through cell–cell junctions, and the whole assembly displays enough cohesion to be used as a standalone EB (Fig. 7b) and sustain stretching. Magnetic stretching is initiated by approaching a second magnetic microtip. The upper cell layers are then pulled against the upper wall (Fig. 7c). At each wall, the magnetic stretcher thus plays the role of a clamp acting on a proximal region of the sample held by the opposing magnetic force and the wall reaction force. At first, for a small distance between the microtips, the intermediate part of the EB is at rest while within each clamp, the wall reaction force exactly balances the corresponding total magnetic attraction force. Moving the clamps apart stretches the intermediate part of the EB and exerts pulling forces (black arrows) on the upper and lower (thin) proximal regions. Modulating the distance between the walls affects the degree of stretching of the large intermediate part (in a uniform manner as shown by the PIV measurements depicted in Fig. 5d) and the corresponding pulling forces and wall reaction forces. Meanwhile, the magnetic forces remain unchanged.

Let us now discuss whether the magnetic forces used to manipulate the magnetic EB can endanger the EB cohesion that results from cell–cell adhesion forces. Adhesive forces are generated by E-cadherin/E-cadherin bonds, measured at 73 pN each, which amounts to about 900 nN per mESC-mESC pair^57^. This is of the same order as the force required to separate two cells (several hundred nN)^58^. One should compare this intensity to the highest tensile cell–cell forces within the aggregate. This happens to be within the intermediate region in the stretched configuration (Fig. 7c). The magnitude of the tensile force in this region is at most equal to that of the total magnetic force, around 1000 nN. This tensile force is distributed over all cells within a horizontal section of the aggregate (for instance the mid-height plane), corresponding to roughly 500 cells. It yields a typical maximum tensile force of 2 nN per cell pair, safely below the mESC-mESC separation force.

It is also important to emphasize that, using only one magnetic microtip does not alter significantly the gene expression (Fig. 4b), while using two magnets, whether in the stretching (static) or cyclic condition, clearly upregulate some genes (Fig. 6a, c). With just one magnetic microtip (Fig. 7a), the tip behaves as a clamp holding a limited (proximal) region of the EB, the only region where substantial forces are present. Indeed, the applied magnetic forces and resulting compression strongly decay with distance from the tip. The major part of the EB thus undergoes negligible stress in this one-magnet situation, and overall gene expression is not affected. By contrast, in the two-magnet situation, the major part of the EB is stretched (Fig. 7c). As a result, gene expression is expected to be altered in most cells, as detected with global PCR measurement.

The magnetic stretcher brings several unique advantages: a 3D aggregate composed solely of its component cells can be formed in situ, without a supporting matrix, and can then be deformed and stimulated, with no transfer step, simply by applying remote magnetic forces. This remote deformation is perfectly illustrated by the immediate release of the tissue when the magnets are removed, and represents another advantage of the approach: the stimulated tissue is easily recovered, perfectly intact. Moreover, it is important to note that this approach is theoretically feasible with any cell type capable of cell–cell adhesion to form a cohesive assembly.

Finally, we show that remote magnetic stimulation (stretching and cyclic stretching) can promote cardiac differentiation without the need of chemical factors. In brief, we found that the stimulation significantly increased the expression of the *Gata4*, *Gata6*, *Sox17* and *Nkx2.5* genes, and that cyclic stimulation had an even more important effect, at day 5 of differentiation. The pattern of these increases is consistent with cardiac mesoderm differentiation (Supplementary Fig. 8). First, the cardiac mesoderm marker gene *Nkx2.5* is importantly upregulated. Then, *Gata4* and *Gata6*, endo/mesoderm genes are also upregulated, and were described to play a decisive role in cardiac differentiation^59, 60^. Finally *Sox17* is overexpressed here as described during cardiomyogenesis^61^. Besides, we then demonstrated that later on, at day 10 of differentiation, later cardiac markers such as troponin T (*Tnnt2*), cardiac α-actin (*Actc1*), α myosin heavy chain (*Myh6*), and myosin regulatory light chain 2 (*Myl2*) were upregulated as well by the stimulation. The use of the magnetic stretcher therefore revealed that mechanical deformation is by itself sufficient to enhance ESC differentiation towards a cardiac phenotype. 2D cell cyclic stretching was similarly found to improve cardiac differentiation and maturation of cardiomyocytes^16, 62^.

Here we do not provide any molecular mechanisms for the cardiac commitment. However, because the mechanical stimulation is that of a global strain applied to the EB, and resembles the situation of a mechanical stretching on a 2D deformable substrate, we can extrapolate from these works how stem cells may translate external forces to mesodermal differentiation. In brief, mechanical stimuli such as stretch and shear stress can activate several signaling pathways such as PI3K/Akt, ERK1/2, GSK-3ß, Tgf-β^63^, Fgf5^64^, and in turn initiate a cardiovascular differentiation program^65–67^, or facilitate cell–cell connections^68^.

To conclude, we proposed here a technology capable of creating embryoid bodies (EBs) from individual embryonic stem cells (ESCs) through the use of remote magnets, while maintaining the cells’ functionality and differentiation profile. This approach is an interesting alternative to conventional techniques for EBs’ formation, such as the hanging drop method. The magnetic stretching and stimulation of the resulting EBs then provided a tool to explore the impact of physical biosensing on ESCs differentiation. This purely mechanical stretching enhanced the EBs towards the cardiac mesoderm differentiation pathway, and this was even more pronounced with the application of cyclic stimulation mimicking heart muscle contraction.

## Methods

### ESC culture

CGR8 cell line was purchased from Sigma-Aldrich (09C028). This cell line was established from the inner cell mass of a 3.5 day male pre-implantation mouse embryo (Mus musculus, strain 129). Regular monitoring of cell cultures for Mycoplasma infections was performed using VenorGeM OneStep Mycoplasma Detection Kit (Sigma-Aldrich). To maintain their pluripotency, the cells were cultured on 0.1% gelatin (Sigma-Aldrich)-coated tissue culture plates in Glasgow’s modified Eagle’s medium (GMEM, Thermo Fisher Scientific) supplemented with 10% fetal calf serum (Thermo Fisher Scientific), 2 mM glutamine (Thermo Fisher Scientific), 1% nonessential amino acids (Thermo Fisher Scientific), 0.1 mM β-mercaptoethanol (Sigma-Aldrich), 1 mM sodium pyruvate (Thermo Fisher Scientific), 1% penicillin/streptomycin (Thermo Fisher Scientific); 1000 U/ml of leukemia inhibitor factor (LIF, Thermo Fisher Scientific) were added prior to use. The cells were cultured at the density of 1.2–10^4^/cm^2^ at 37 °C in a humidified 5% CO2-95% air atmosphere and passaged every 2 days.

### Iron oxide magnetic nanoparticles

Iron oxide nanoparticles (provided by PHENIX, UMR 8234, Paris) were synthetized by alkaline coprecipitation of FeCl2 (0.9 mol) and FeCl3 (1.5 mol) salts. The nanoparticles were then oxidized into maghemite with 1.3 mol of iron nitrate under boiling. After magnetic decantation, the maghemite nanoparticles were heated at 80 °C for 30 min in water, then supplemented with sodium citrate (70 g) to promote absorption of citrate anions onto their surface (to ensure electrostatic stabilization in aqueous solution) before precipitation in acetone at 25 °C and resuspension in water. The resulting nanoparticles were 8 nm in diameter, with polydispersity index of 35%.

### Magnetic cell labeling

Before their incubation with ESCs, the nanoparticles were dispersed at final [Fe] concentrations between 0.2 and 5 mM in RPMI (Thermo Fisher Scientific) supplemented with citrate at a final concentration of 5 mM to prevent nanoparticle aggregation. The ESCs were incubated in this medium between 30 min and 4 h, rinsed twice with RPMI medium, and then returned to complete proliferation medium (containing LIF) for at least 2 h before use.

### Determination of the cell iron mass by magnetophoresis

The mass of iron incorporated by the cells was determined by magnetophoresis, based on the determination of radius (R_cell_) and velocity (v_cell_) of single cells dispersed in aqueous medium (viscosity η  = 10^3^ Pa.s) and attracted by a magnet whose field and gradient were perfectly calibrated (B = 0.15 T; gradB = 17 T/m). Calculation of the cellular iron mass is straightforward: In the horizontal plane of cell movement, the magnetic force (M_cell_gradB) is counterbalanced by Stokes’ viscous force (6πηR_cell_v_cell_), thus providing the magnetic moment (M_cell_) of the analyzed cell, which can be transformed into the mass of iron internalized via the volumic magnetization (50 emu/g for the field B of 0.15 T). For each magnetophoretic measurement, the radius and velocity of 200 cells were measured using image processing (Image J). The cellular iron mass (in pg of iron per cell) obtained corresponds to the average value for the cell population (distribution of about 35%), itself averaged over at least 3 independent experiments.

### Perls staining of intracellular iron

Perls’ Prussian blue staining was used to reveal iron-labeled cells in blue. The cells were fixed in 10% formalin solution in PBS then rinsed with PBS and incubated for 30 min at room temperature with 1% potassium ferrocyanide in 1% aqueous solution of hydrochloric acid. They were then rinsed with PBS and observed by optical transmission microscopy.

### Cell viability testing

The Alamar Blue metabolic test (Thermo Fisher Scientific) was used to assess the impact of the magnetic nanoparticles on ESC viability. The active ingredient, resazurin, becomes highly fluorescent when metabolized. Fluorescence was quantified with a spectrophotometer (excitation 530 nm, emission 590 nm), and the values were interpreted relative to control values (unlabeled cells in complete medium) obtained under similar conditions.

### EB magnetophoresis for monitoring nanoparticles fate

To measure the magnetic moment (M) of the EBs, single EBs were immersed at each different time point after formation (days 1, 2, 4, and 7, *n* > 8 for each condition) in a glycerol solution (80%, room temperature 23–24 °C, viscosity η = 0.05 Pa.s) submitted to a magnetic field gradient (B = 150 mT, gradB = 17.5 T/m) generated by a permanent magnet (cylinder 25 mm in diameter, 10 mm height). Each EB thus experiences a magnetic velocity v_mag_ towards the magnet, by balancing the magnetic force MgradB, and the Stokes drag force 6πηRv_mag_, where R is the EB radius. EB migration was video-monitored every 0.1 s (×4 objective, Leica DMIRB microscope). The magnetic moment calculated (in A.m^2^, at 150 mT) can be converted to grams of (magnetic) iron (68 emu/g at 150 mT, 1 A.m^2^ = 10^3^emu).

### Relative quantification of gene expression by real-time PCR

Total RNA was isolated using NucleoSpin RNA kit (Machery-Nagel) according to the manufacturer’s instructions. To avoid genomic DNA contamination, RNA samples were incubated for 15 min with 10U of DNase. Complementary DNA (cDNA) was then synthesized using SuperScript II Reverse Transcriptase kit (Thermo Fisher Scientific) with random hexamers according to the manufacturer’s instructions. Real-time PCR analysis was then carried out with SYBR green PCR technology using the StepOnePlus system (Thermo Fisher Scientific). The expression of 60S acidic ribosomal protein P0 (*RPLP0*) was used as a reference transcript. All sequences of primers used are presented in Supplementary Table 1.

### EB formation by the hanging drop method

Two days after passage, cells were detached, centrifuged and resuspended in differentiation medium (GMEM supplemented with 20% fetal calf serum, 2 mM glutamine, 1% nonessential amino acids, 0.1 mM β-mercaptoethanol, 1 mM sodium pyruvate, 1% penicillin/streptomycin). Note that the LIF factor must be removed from culture medium since day 0 of EBs formation, resulting in initiation of differentiation. Drops containing 1000 or 10,000 cells in 30 μl of medium were plated as hanging drops on a lid of 10 cm non-adherent Petri dish, which was then inverted over the bottom of the dish filled with PBS to prevent drying. After 2 days (D0–D2), the aggregates thus formed were transferred to a Petri dish containing 10 ml of differentiation medium for a 3-day maturation period (D2-D5). Mature aggregates were finally transferred to 0.1% gelatin-coated 24-well dishes. The medium was changed every 2–3 days.

### EB formation by magnetic attraction

Cells labeled with magnetic nanoparticles were detached, centrifuged and resuspended in differentiation medium. They were then placed in a glass-bottom Petri dish. To prevent cell adhesion, petri dish was previously incubated for 30 min with 10 mg/ml PLL-PEG (SuSos) diluted in 10 mM HEPES, then rinsed with sterile water, and placed on a magnetic device composed of several magnetic attractors. The magnetic device fabrication is straightforward. First make holes (typically 9 or 16, arranged in a square 3–4 mm lattice) with 0.8 mm drill through aluminum cylindrical plates (Dural) 8 mm thick and 35 mm diameter to match the size of small Petri dishes; Then take typical sewing pin, to be used as soft-iron cylinders with a diameter of 750 μm. Insert the pins in the holes, and cut at the plate surface (use a drilling machine to level the surface; Place this magnetic pins array over a permanent magnet (typically disc neodymium magnet Ø 20 mm diameter, 8 mm height, strength about 10 kg, magnetic field created at the surface ~0.4 T). The device is ready to be used. Place it over a Petri dish with glass bottom, and deposit the ESCs in culture medium. The number of deposited cells was adjusted to obtain between 1000 and 30,000 cells per magnetic microtip. The magnetic device was subsequently removed between 5 min and 2 days, depending on the experiment.

### Formation and stimulation of EBs in the magnetic stretcher

The magnetic stretcher is described in detail in the results section. It consists of a reservoir with two magnetic structures, one fixed and the other mobile, each comprising 3 magnetic microtips and the 3 magnets used to magnetize them. The principle is to form 3 EBs over the 3 fixed magnetic microtips of the fixed structure, and then stretch them by the attractive force of the 3 matching microtips on the mobile structure. They could also be stimulated at will by subjecting the second structure to micro-controlled movements.

The reservoir was first washed in 70% ethanol for 15 min, then dried and rinsed 3 times with sterile water before being exposed to UV for 30 min. The glass slides protecting the magnetized microtips were coated with PLL-PEG to prevent cell adhesion. Before use, the stretching device was sterilized by UV and placed in a sterile box, with a sterile cover placed over the reservoir.

Three EBs, each composed of 10,000 ESCs (labeled with 2 mM iron for 30 min), were formed on day 0 on the fixed set of magnetic microtips. One hour later they were stretched, at amplitude corresponding to 50% of their original size, by approaching the mobile set of magnetic tips (stretched condition). The whole assembly was then placed overnight in an incubator (37 °C, 5% CO_2_). In some cases (cyclic condition), starting the following day (day 1), the EBs were stimulated twice daily for 2 h at a frequency of 1 Hz and an amplitude corresponding to 10% of the initial height of the aggregate. Stimulation was applied for the next 3 days (day 1–day 3). At the end of day 3, the microtips were demagnetized by removing the permanent magnets. This instantly released EBs were transferred to Petri dishes and again allowed to mature for 2 days (up to day 5) before analysis.

### Fluorescence live imaging

Cell membranes were stained with a red fluorochrome Pkh26 from Sigma. Cell stimulation with a 10% strain applied at 1 Hz was observed in situ, on living cells, by fluorescence microscopy.

Velocity mapping: The PIV analysis was computed using the Matpiv software package (a GNU public license software) for MATLAB (The MathWorks, Natick, MA)^69, 70^. We used 64 × 64-pixels (40 × 40 µm) interrogation windows with 75% overlap. Calculation of the correlation between two successive subwindows was performed by fast Fourier transform (the single method). Aberrant vectors were filtered out from the velocity fields with a median Gaussian filter.

### Immunohistology

16-µm cryosections of EBs were fixed with 4% PFA (Interchim) during 5 min, washed with PBS, and then incubated 5 min with PBS-Triton X-100 (0.1% v/v). Nonspecific sites were blocked with 5% (w/v) bovine serum albumin (BSA) diluted in PBS during 1 hour and incubated with primary antibodies overnight at 4 °C. Anti-Brachyury T (Abcam, 1:200) and anti-Nkx2.5 (Santa Cruz Technologies, 1:100) primary antibodies were used. The binding of primary antibodies was detected by incubation for 3 h with Alexa Fluor-conjugated anti-rabbit IgG (Cell Signaling, 1:1000). Finally, cells were washed in PBS and mounted with Prolong®Diamond Antifade Mounting Medium with DAPI (Life Technology) for nuclear staining. Cells were analyzed with an Olympus JX81/BX61 device/Yokogawa CSU device spinning-disk microscope (Andor Technology), equipped with a 63X oil objective (Olympus).

### Statistical analysis

All measurements were made at least three times, in independent conditions. All results are shown as the mean ± standard error of the mean (SEM). Parametric student’s *t* test, two-sided, was used to compare the mean of two values obtained for two independent conditions; *p < 0.05 indicates a significant result, **p < 0.01 a very significant result, and ***p < 0.001 a highly significant result.

### Data availability

Data supporting the findings of this study are available within the article and its Supplementary information files, and from the corresponding author upon reasonable request.

## Electronic supplementary material

Supplementary Information

Peer Review
