# Supplementary material for: A 3D magnetic tissue stretcher for remote mechanical control of embryonic stem cell differentiation
Source: Nat Commun. 2017 Sep 12;8:400. doi: 10.1038/s41467-017-00543-2 (PMC5596024; doi:10.1038/s41467-017-00543-2)
Supplement: Supplementary file 1 — Supplementary Information [file 41467_2017_543_MOESM1_ESM.pdf]

Description of Supplementary Files

File Name: Supplementary Information

Description: Supplementary Figures, Supplementary Table and Supplementary References

File Name: Peer Review File

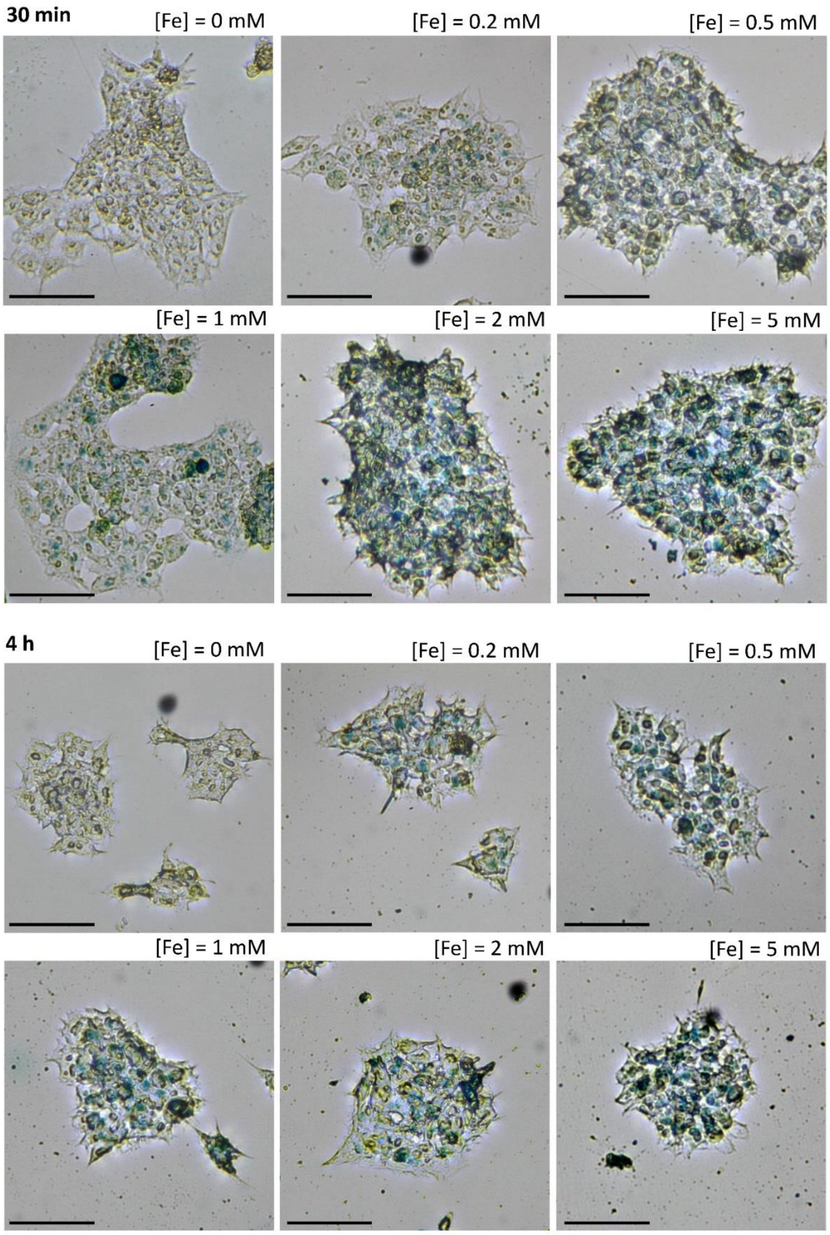

Supplementary Figure 1. Perls' Prussian blue staining of irons in ESCs. ESCs were labeled for different incubation conditions, 30 min or 4 h incubation times, and [Fe] in between 0.2 to 5 mM. Control corresponds to [Fe]=0 mM. Scale bar: 250 µm.

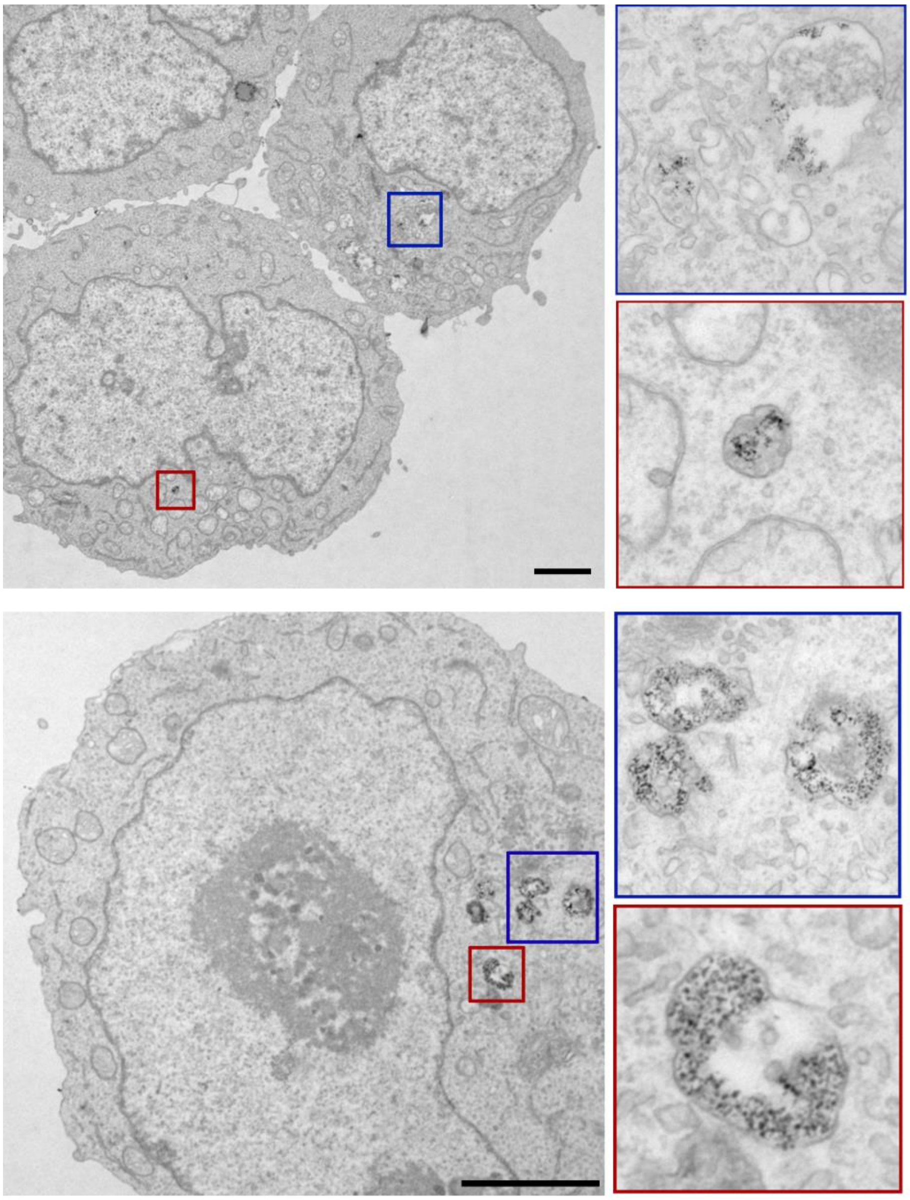

Supplementary Figure 2. Transmission electron micrograph of an ESC after labeling. Different electron microscopy views of magnetic nanoparticles internalisation, after 30 min incubation at [Fe]=2 mM. For all ESCs observed, the cellular morphology was unchanged, and nanoparticles were systematically located within endosomes (zooms of framed areas), none of them being detected outside the cells. Scale bar: 2 µm.

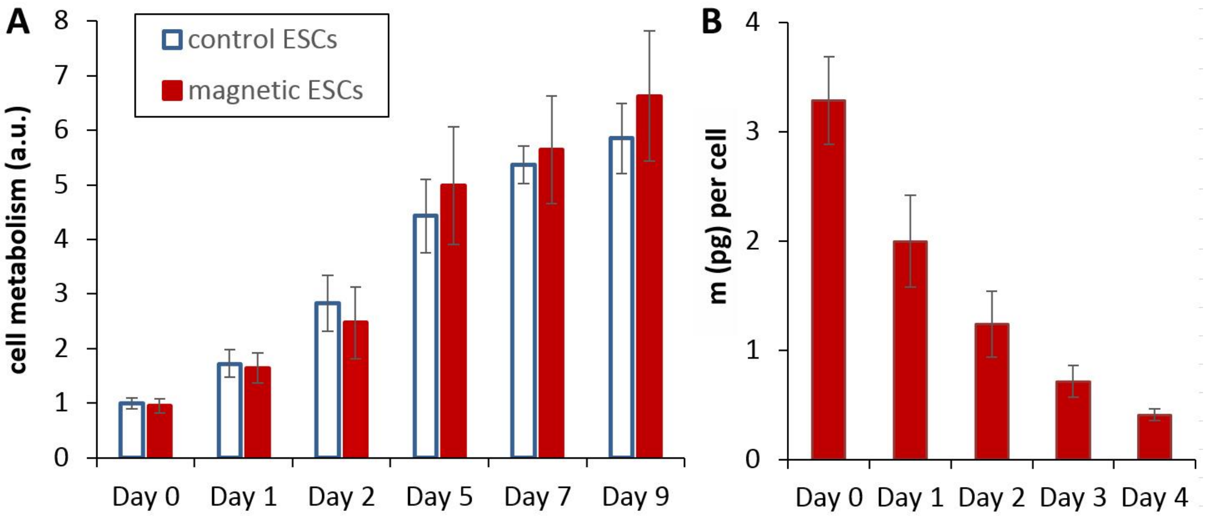

Supplementary Figure 3: **Cell viability and nanoparticles dilution over cell division**. **A**. Cell metabolism measured by Alamar Blue assay on standard 2D ESCs culture at different times after incubation with magnetic nanoparticles (cellular uptake of 3.2±0.2 pg of iron per cell): first day (day 0, two hours after incubation); 24 hours after (day 1); 48 hours after (day 2); and then 5, 7 and 9 days after. It shows that cell viability is not impacted by the intracellular presence of the nanoparticles, over long-term periods. **B**. Intracellular mass of iron (expressed in pg per cell) measured each day by single-cell magnetophoresis, during the 4 days following the initial nanoparticles incorporation (day 0). The cellular iron decrease follows the same trend as the cell proliferation on part A, demonstrating that each cell shares its content between the daughter cells during division. One can however note that the decrease in cellular iron mass is slightly more marked than the increase of cell number, revealing a degradation of the nanoparticles inside the endosomes (as quantified in Fig. 3e), more likely than a nanoparticles expulsion, which we never observed in normal culture condition.

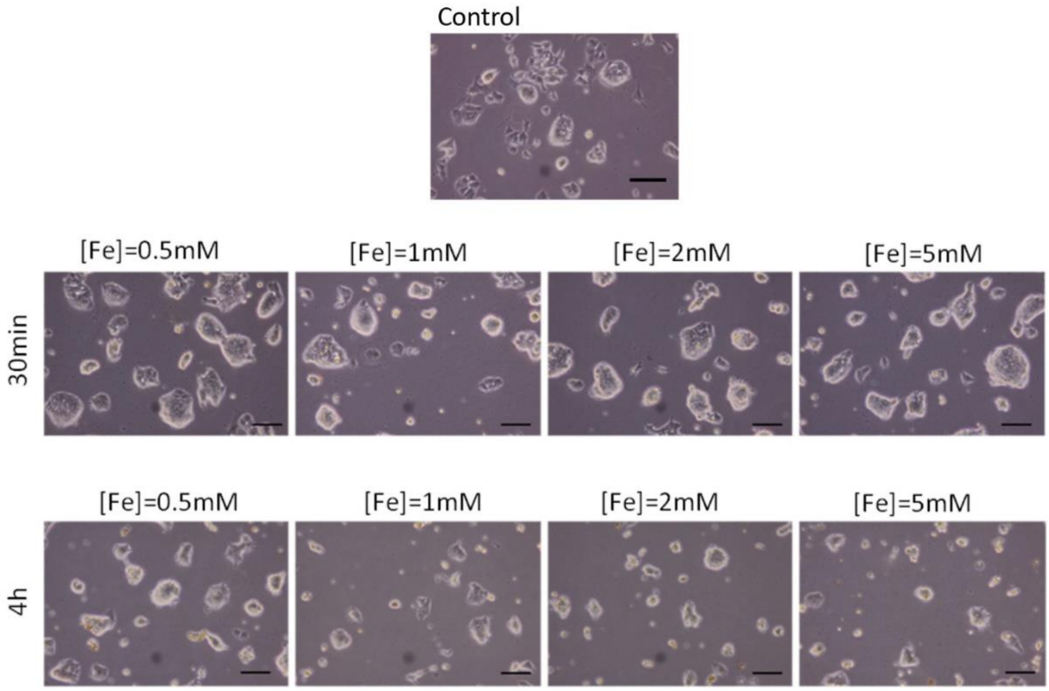

Supplementary Figure 4. Follow-up of ESCs colony morphology by optical microscopy. For 30 min incubation, whatever the iron concentration, ESCs colony morphology is similar to the control. For 4 h incubation, the colonies were slightly impacted, especially at high iron concentration, appearing more rounded and smaller. Scale bar = 100 µm.

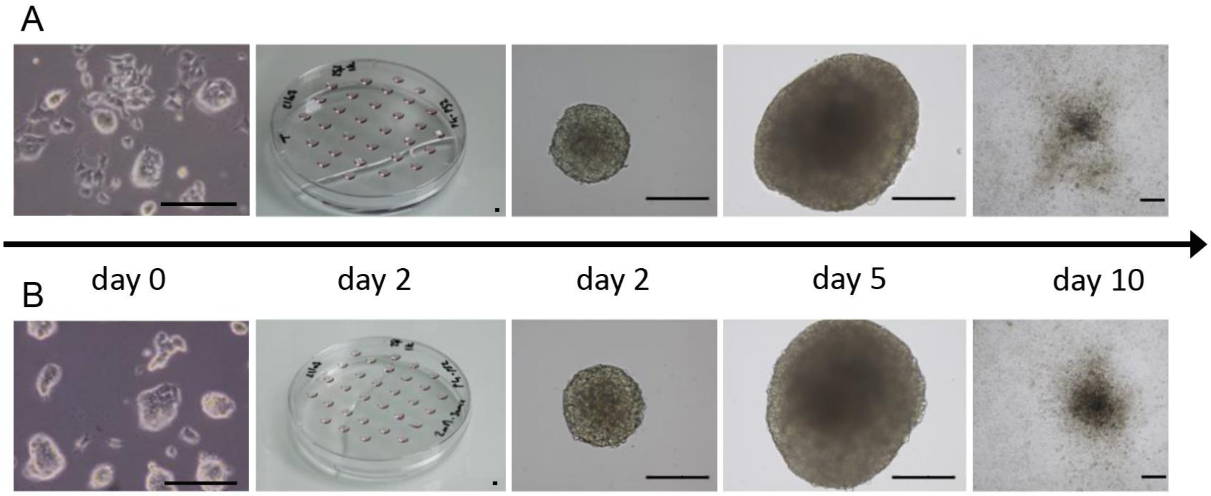

Supplementary Figure 5: Different stages of EB formation and differentiation using the hanging drop method. A) Control ESCs; **B)** Magnetic ESCs. On day 0, the cells were detached, and drops containing 1000 cells were deposited on the lid of Petri dishes, on which they remained until day 2. Magnetic labeling was carried out 4 h before detachment. At the end of day 2, each drop contained small EBs (about 250 microns in diameter), which were then transferred to non-adherent Petri dishes and allowed to grow until D5, when they reached a diameter of about 450 microns. They were then again transferred to gelatin-coated multi-well plates, where they spread out (seeding). A seeded EB is shown after 5 days (D10, last image). Scale bar : 200 µm

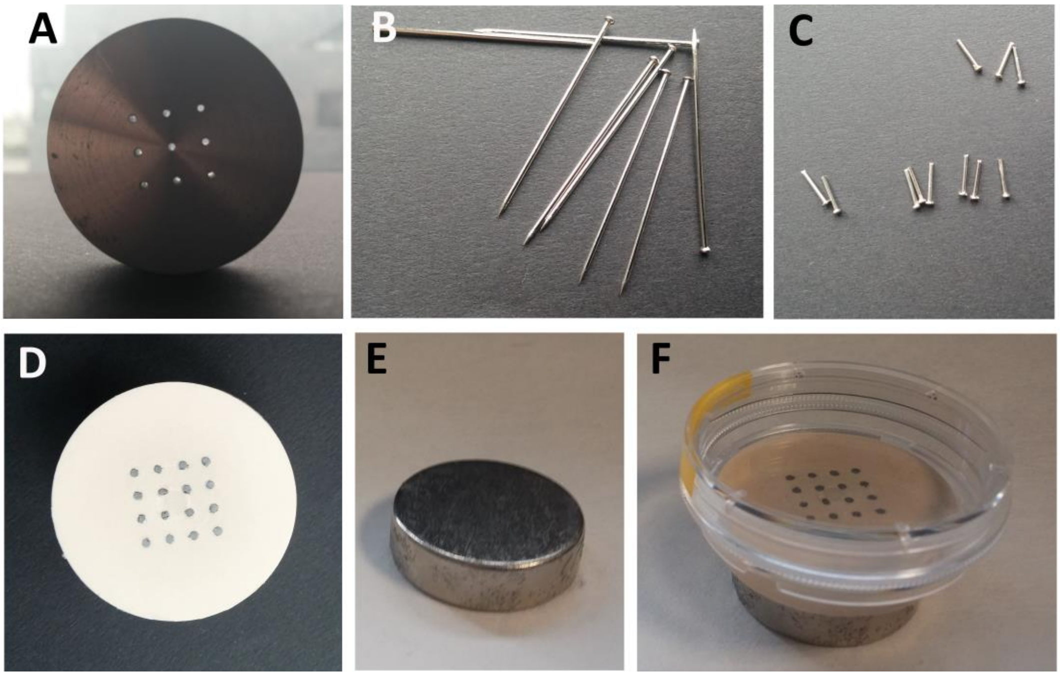

Supplementary Figure 6: **Magnetic device including micro-magnets to form EBs**. **A**) Cylindrical plate with 0.8 mm holes. **B**) Typical sewing pins. **C**) Same pins after cutting. **D**) Magnetic array with pins inserted. **E**) Typical disc neodymium magnet. **F**) Whole device placed over a Petri dish.

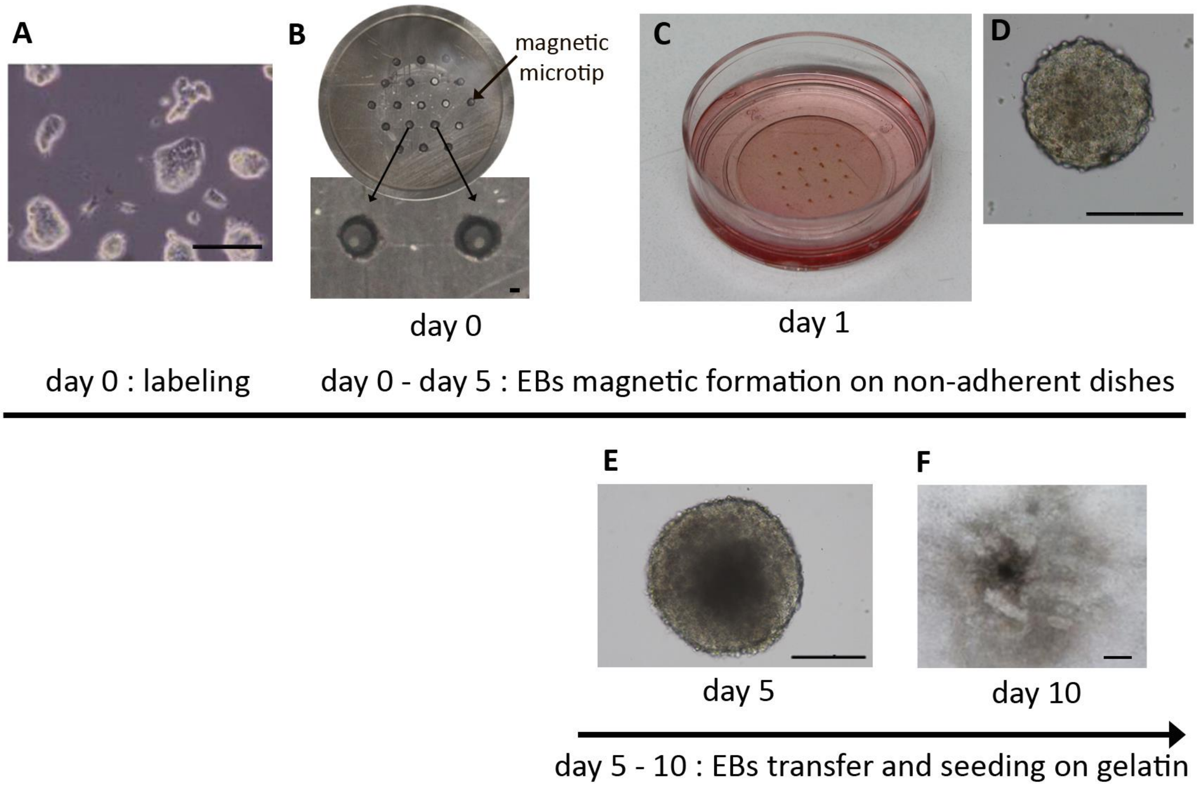

Supplementary Figure 7. Different stages of embryoid body formation by magnetic attraction. A) ESCs before magnetic labeling. Cells were labeled on day 0, detached and subjected to the magnetic attraction of the microtips to form EBs. **B)** The EBs so-formed can be seen at day 0 in the dish placed on the microtips network (photographed from above at day 0, with a zoom on two networked microtips). Here, 1000 cells were deposited per microtip. **C**) The EBs stays at their initial network position when the magnetic array is removed (photograph taken at day 1 after magnetic EBs formation. **D**) Microscope image of a typical EB at day 1. From day 0 to day 5, the EBs stays in the non-adherent Petri dishes used for the initial magnetic formation. **E)** On day 5, the EBs were transferred and seeded in gelatin-coated wells. **F)** Image of an EB on day 10. All scale bars: 200 μm.

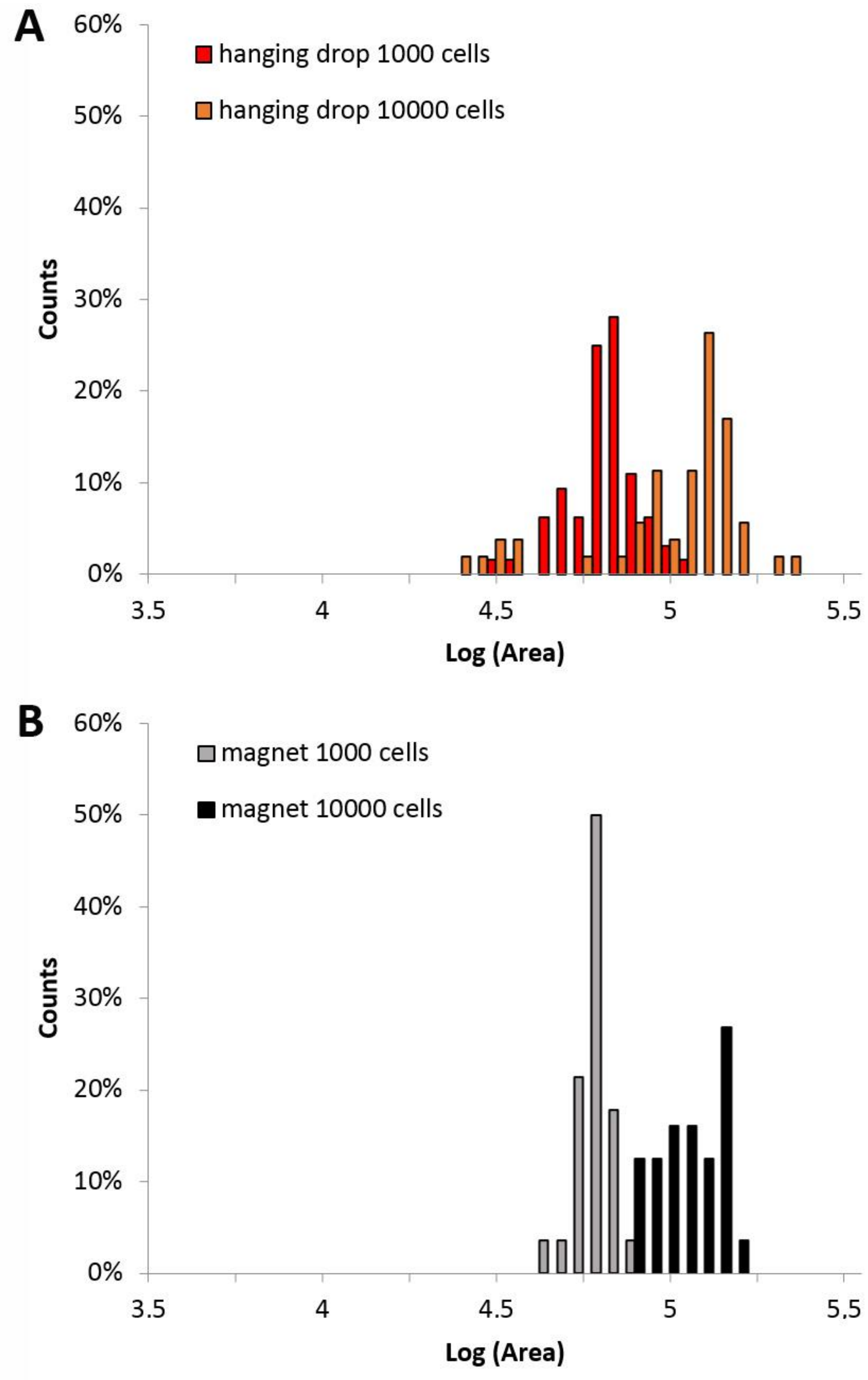

Supplementary Figure 8. Comparison of magnetic EB formation and hanging drop. Morphological analysis of EBs formed by the hanging drop (**A**, day 2, 1000 and 10000 cells) or magnetic method (**B**, day 2, 1000 and 10000 cells. The base-10 logarithm of sectional area is plotted on a histogram, demonstrating the increase in size control for magnetic formation over hanging drop.

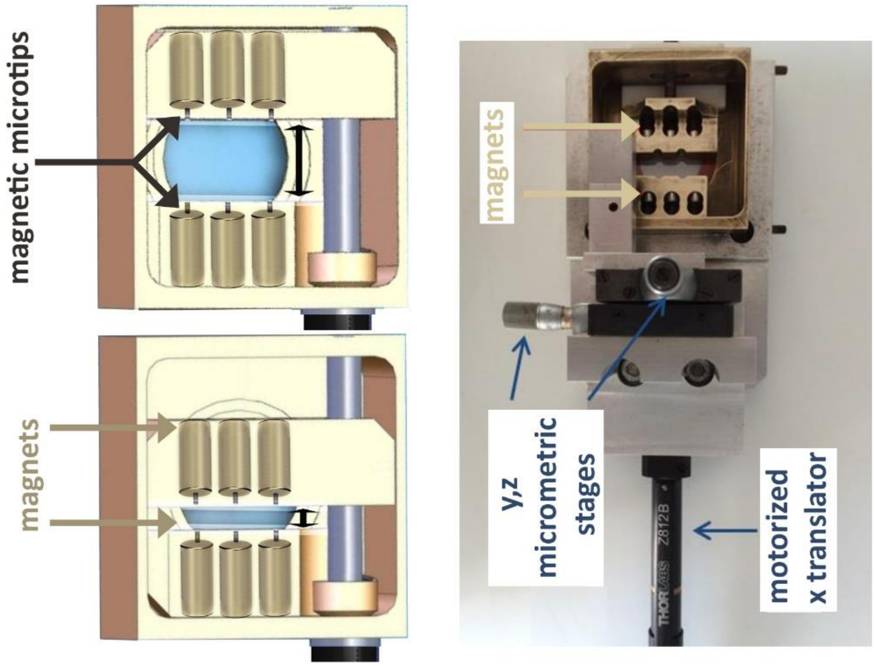

Supplementary Figure 9. Schematic and photographic representations of the magnetic stretcher. The Thorlabs motor was used to control the mobile support (along x), which could also be adjusted along y and z by two micromanipulators. The magnetic microtips are hidden inside the support and isolated from the medium by glass coverslips. Note the magnet emplacements, which have been opened to recover the EB.

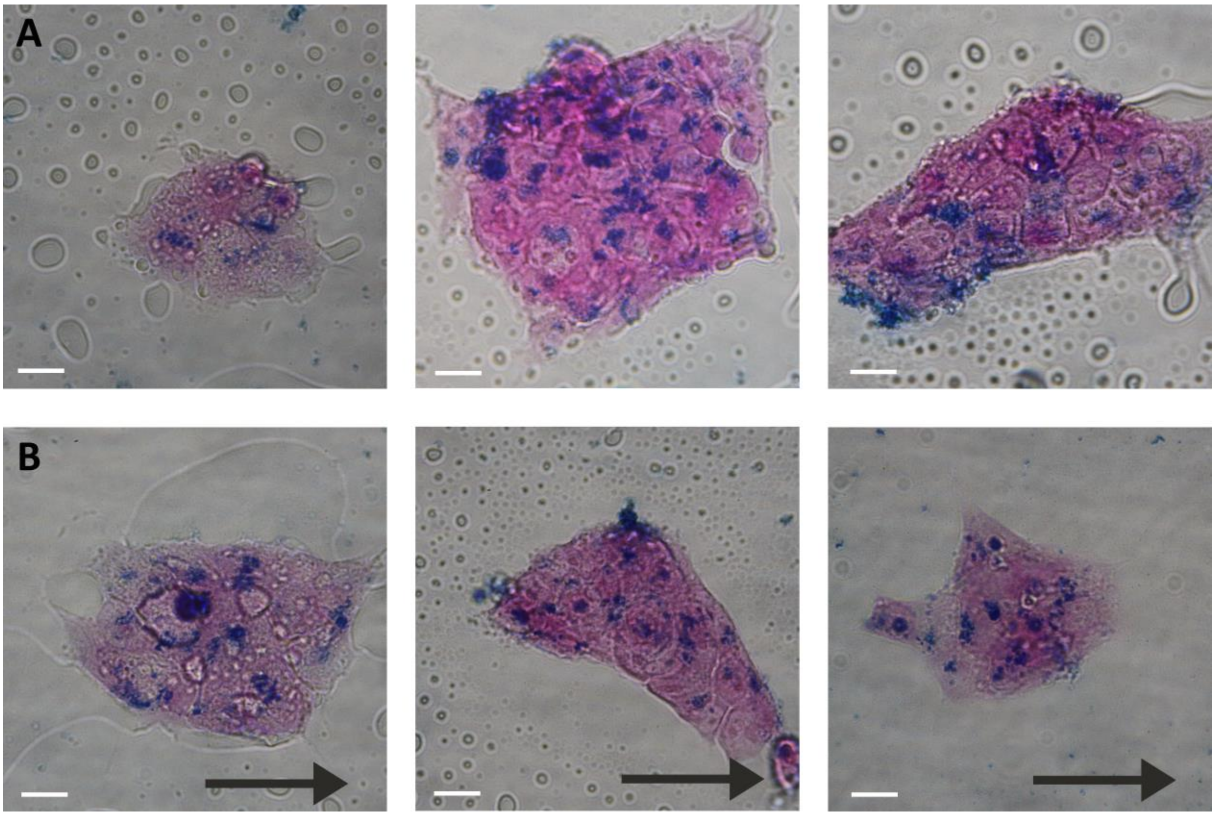

Supplementary Figure 10. Magnetic lysosomes intracellular pattern with and without the application of an external magnetic field. A) Observation of the magnetic lysosomes patterns inside the cells for control condition (no magnetic field). **B**) Lysosomes patterns when submitted to a magnetic field gradient grad B of 1000 T/m (black arrow). The lysosomes are detected by Prussian Blue staining, which colors iron in blue. No lysosomes agglomeration can be seen when the magnetic force was applied (B), as compared to without any magnetic field application (A). All scale bars: 10 µm.

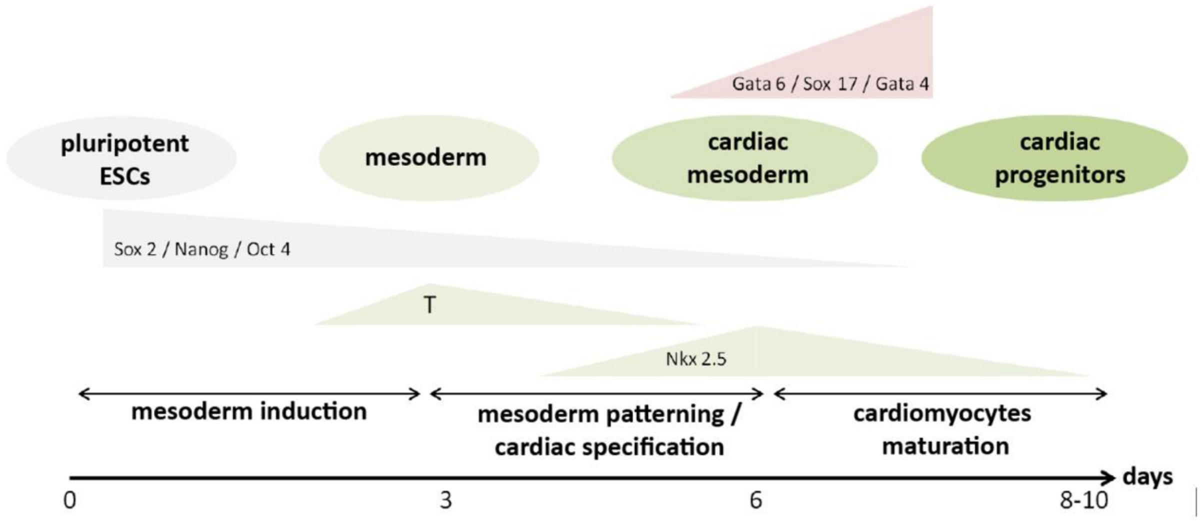

Supplementary Figure 11. Expression of genes necessary for ESC differentiation into cardiac cells. As soon as LIF is removed from the medium (day 0), the pluripotency genes start to be down-regulated, as observable from the 3rd day. Expression of brachyury (*T* gene product) begins to increase, peaks around day 3, then falls rapidly until day 6. *Nkx2.5* gene expression then rises sharply on day 6. The expression level of the *Gata 4*, *Gata 6* and *Sox 17* genes increases from D5 but does not diminish on D7, contrary to the *T* gene.
Adapted from [1, 2].

Genes	Primers sequences	Genes	Primers sequences
RPLP0	Fwd: GCCAGCTCAGAACACTGGTCTA	*T*	Fwd: CCCCAATGCCATGTACTCTTTC
	Rev: ATGCCCAAAGCCTGGAAGA		Rev: TTCCAGCGGTGGTTGTCA
Fn1	Fwd: GGT-GTA-GCA-CAA-CTT-CCA-ATT-ACG	*Nkx2.5*	Fwd: CTCCGCCAACAGCAACTTC
	Rev: GGA-ATT-TCC-GCC-TCG-AGT-CT		Rev: GGACTCTGCACGGTGTTCAA
Lama1	Fwd: ACAGAAGGAAAGACGATCGACATAT	*Wt1*	Fwd: TCT-TCC-GAG-GCA-TTC-AGG-AT
	Rev: TTAATACACCCATGGAACGAAGTC		Rev: TGC-TGA-CCG-GAC-AAG-AGT-TG
Lamb1	Fwd: TGG-AGC-TGC-CCC-AGT-ATA-CG	*Nes*	Fwd: AGCCATTGTGGTCTACGGAAGT
	Rev: AGC-GAG-TCG-ATG-AAC-GTG-TAA-G		Rev: TCCACACACCCCAGTGGTT
Lamc1	Fwd: TTT-TGA-TAG-ACG-CGT-GAA-CGA	*Pax6*	Fwd: CCATGTTGGGCCGAACA
	Rev: TGG-CGG-GAA-TTC-TCC-TTA-GAG		Rev: ATGGGTGGCAAAGCACTGTAC
Sox17	Fwd: ATA-AGC-CCG-AGA-TGG-GTC-TTC	*Sox2*	Fwd: CTGGACTGCGAACTGGAGAAG
	Rev: CCG-TGG-CTG-TCT-GAG-AGG-TT		Rev: TTTGCACCCCTCCCAATTC
Gata4	Fwd: CCT-CCC-GCA-CGA-TTT-CTG	*Oct4*	Fwd: TTCCCTCTGTTCCCGTCACT
	Rev: CTC-AGG-AAA-AAG-AAA-ATC-CCA-AAT-T		Rev: TGGTGCCTCAGTTTGAATGC
Gata6	Fwd: TCC-CCT-GCC-GAA-GTC-ACA	*Nanog*	Fwd: AGGCCTGGACCGCTCAGT
	Rev: GGC-CAG-AGC-ACA-CCA-AGA-A		Rev: AGTTATGGAGCGGAGCAGCAT
TNNT2	Fwd: CGA-CCT-GCA-GGA-AAA-GTT-CAA	*Myh6*	Fwd: CGA-CAT-CAG-TCA-GCA-GAA-CAG
	Rev: TCC-GGT-TTC-GCA-GAA-CGT		Rev: GTC-AGA-GCG-CAG-CTT-CTC-C
Myl2	Fwd: CCA-GCA-GGC-TCC-TCG-AAC-T	*ACTC1*	Fwd: GCT-TCC-GCT-GTC-CAG-AGA-CT
	Rev: TTT-GGT-GCC-ATG-GTG-TCT-GA		Rev: TGC-CAG-CAG-ATT-CCA-TAC-CA

Supplementary Table 1. Primers sequences used in real-time PCR analysis (5’ to 3’).

Supplementary References

1. Nakanishi M, *et al.* Directed induction of anterior and posterior primitive streak by Wnt from embryonic stem cells cultured in a chemically defined serum-free medium. *The FASEB Journal* **23**, 114-122 (2009).
2. Willems E, Bushway PJ, Mercola M. Natural and synthetic regulators of embryonic stem cell cardiogenesis. *Pediatr. Cardiol.* **30**, 635-642 (2009).
