# Supplementary material for: A 3D magnetic tissue stretcher for remote mechanical control of embryonic stem cell differentiation
Source: Nat Commun. 2017 Sep 12;8:400. doi: 10.1038/s41467-017-00543-2 (PMC5596024; doi:10.1038/s41467-017-00543-2)
Supplement: Supplementary file 2 — Peer Review [file 41467_2017_543_MOESM2_ESM.pdf]

Reviewers' comments:

Reviewer #1 (Remarks to the Author):

In this study, the authors investigate whether mechanical factors alone can drive differentiation of mESCs. They developed a magnetic technique to assemble single cells into 3D multicellular spheroids resembling an embryoid body formation. This technique also allows them to stretch or apply cyclic loading on the multicellular spheroids. Due to the stretching load, the EBs committed toward cardiac mesoderm lineage. I agree with the authors that making three dimensional tissue structure from individual cells and driving differentiation will advance the fields of biophysics and regenerative medicine. This is an important topic and should be of interest to a large audience. There are, however, a number of issues that should be addressed before considering the manuscript for publication.

1. The fact that "purely mechanical factors" can drive ESC differentiation, which is the main objective of this work, has been reported by many previous studies. Hence, the manuscript lacks novel findings.
2. What is the efficiency of EB formation in your method? Can you discuss how the magnetic attractor method is much simpler and more advantageous than hanging drop method? If controlling EB size is the only advantage over hanging drop method, they can also be achieved by AggreWell plates or an ultra high throughput technique developed by Peter Zandstra lab (Ungrin et al., PLoS ONE, 2008). Can you compare your technique with the ones mentioned above?
3. Were the endocytosed magnetic nanoparticles coated with targeting proteins? Or are they non-specific? Did the nanoparticles cluster inside the cells or remain dispersed after applying the magnetic field? What would be the effect of smaller forces arising from dispersed particles vs larger forces from agglomerated particles? I believe the cell will interpret applied magnetic field, i.e., the force, differently if the particles remain dispersed as opposed to agglomerated condition.
4. What would be the global stress or resultant strain per cell? Is it the stress or the strain that orchestrated mechanotransduction pathways during the cell differentiation? Can you provide some mechanistic insight? Please see earlier papers from the labs of Michael Sheetz, Dennis Discher, Ning Wang (del Rio et al., Science, 2009; Johnson et al., Science, 2007, Chowdhury et al, Nat Mater, 2010) for more details.
5. The manuscript lacks single cell analysis within the EBs and do not provide molecular mechanism of differentiation into mesoderm germ-layer.

Minor:
1. What is the reason for using RPLP0 as normalizing gene in your qPCR experiments? Why not the commons normalizing genes such as GAPDH or EF1a?
2. Overall, the introduction section lacks citations of relevant previous works. For example, in line 43-45, the cited reference on the effect of substrate rigidity on cell differentiation do not refer to Engler et al., Cell, 2006 paper. In addition, hESC related references do not apply to this study.

Reviewer #2 (Remarks to the Author):

In this manuscript, Du et al devise and test a novel method to generate and differentiate embryoid bodies from murine embryonic stem cells. This method utilises iron nanoparticles, which can be engulfed by the cells, allowing the cells to be mobilised using magnets. The authors first optimise the concentration of nanoparticles and duration of application to allow sufficient uptake of the particles but without causing overt toxicity. They test the capacity of labelled cells to aggregate into EBs and observe no overt difference compared with unlabelled ESCs using the hanging drop method. They then test the capacity of the nanoparticle-laden cells to aggregate in response to a magnetic stimulation, and thus efficiently generate EBs, which exhibit the same differentiation

potential as unlabelled cells. Using movable magnets, they then apply forces to stretch the EBs (by single pulse or cyclic stimuli to resemble functioning cardiac muscle) and observe their differentiation potential compared with unstretched EBs. They show enhanced expression of a panel of differentiation markers using qRT-PCR and some confirmatory immunohistochemstry of Brachyury and Nkx2.2. The consequences of cell stretching on differentiation have been previously reported, but not using ESCs, so the demonstration of the effect in this system that the authors equate to embryonic development is novel. However, in order for the technology to be adopted by the pluripotent stem cell field a more directed differentiation approach would need to be demonstrated as well as evidence that the engulfment of iron nanoparticles is consistent with longer term stable culture. Although the manufacture and initial validation of the system are interesting, the application to regenerative medicine are not obvious.

Reviewer #3 (Remarks to the Author):

- **What are the major claims of the paper?**

In 'An all-in-one magnetic tissue stretcher for forming and stimulating in situ embryoid bodies: towards remote mechanical control of stem cell differentiation', the authors apply and optimize mouse embryonic stem cells (mESCs) magnetic labelling and assess its impact, together with magnetic stretching, on embryoid body (EB) formation and differentiation.

More specifically, the authors address three questions/issues:

1. 'The impact of nanoparticle internalization on the cell phenotype, and particularly differentiation capacity' in mESCs.
2. 'Whether 3D magnetic "printing" of ESCs could be equivalent' and 'simpler' to EB formation using the hanging drop method.
3. 'Could magnetic forces alone drive stem cells differentiation within a magnetically formed 3D model tissue?' According to the manuscript, it is 'evidenced the impact of purely mechanical stimulation on EB differentiation' as the magnetically formed EBs(=magnetic-EBs) seem to have enhanced differentiation toward cardiac fate.

The authors address these questions using a combination of qPCR and immunofluorescence analysis of specific genes (pluripotency and differentiation, depending on the experiment), cell viability tests and visual inspection of the generated EBs. In summary, the authors incorporate the magnetic nanoparticles in mESCs, generate EBs which can be deformed using magnetic forces which seems to enhance differentiation towards all embryonic layers, which is not per se very interesting or attractive. Although the study presented here shows some novelty and is of potential interest, overall, the work presented is still in a preliminary state with no clear major contribution to the field. A number of issues need to be addressed before I can recommend its publication.

Specific major comments:

For question No1,

1. This part of the study is not novel as Parsa et al.[1] did also used iron oxide nanoparticles and analysed how they affected mESCs viability, pluripotency and differentiation using EB formation. Magnetic nanoparticles have been used multiple times in the literature in the context of adult stem cells, as indicated by the authors citing some of the relevant papers. The synthesis of the nanoparticles used in the present manuscript is not detailed enough to distinguish how different these nanoparticles are form others used in the literature.
2. The authors show that, if they incubate the cells for 30min in 2mM [Fe], the used nanoparticles do not affect mESC pluripotency following the expression of NANOG and SOX2. However there is an increase (statistically significant) of OCT4. How do the authors explain this? Does this increase of OCT4 have an impact on mESC behaviour? It has been previously shown that specific, tightly regulated levels of OCT4 are key for pluripotency[2], the increase shown here can be potentially affecting the magnetized mESCs.
3. On a related topic to point 2, the authors do not show what the long term effect of the nanoparticles in the mESCs is.

It would be good that the authors would comment on these issues.

Also the following should be tested:

-for how long are the nanoparticles maintained in the cells once they've been endocytosed? If the authors passage the cells, after how many passages do these particles disappear? This is an important issue if the application of this technique will eventually be to do in vivo work.

-the cell viability tests shown are performed after 30min or 2-4h after incubating the cells with the nanoparticles, if the incubation is done for 30min and then viability tests are done a few days after, do the magnetised cells show a decreased viability?

For issue / question 2, the authors show that their method results in equivalent EBs (differentiation marker expression profile shown in Fig. 2) as shown in[1]. They propose that generating EBs using magnetic forces is a simpler method as the magnetic-EBs are less variable in size and more homogeneous than the traditional hanging drop-EBs. However, besides a couple of images, this has not been quantified. On this, it would be advisable that the authors undertake a detailed comparison between both methods showing measurements of EB numer, size and sphericity to show that magnetic-EB are more efficiently formed. Anyway the proposed method is not simpler as it required the use of custom made devices which are not at hand to other researchers.

On issue / question 3, and this is the issue that the authors really try to oversell:

1. They show that the magnetically formed EBs (=magnetic-EBs) do differentiate in an equivalent manner to the hanging drop-formed EBs (albeit with different levels of expression of several markers, see below). Contrary to what the authors claim, this does not show that mechanical stimulation does drive differentiation: in magnetic-EBs with continuous or cyclic stretching differentiation occurs following the same pattern of gene expression as in otherwise formed EBs, albeit with enhanced expression of all embryonic layers. This indicates that magnetic forces alone do not drive differentiation, if any, they enhance differentiation. If the authors want to claim this, they should show that in the absence of differentiation cues, magnetic forces do drive differentiation. In the presented experiment, EBs are formed which in itself is a source of differentiation cues, therefore this is not a suitable model to address whether magnetic forces do drive differentiation. This is not something easily addressed experimentally, as the suitable model would require absolutely no differentiation cue but the magnetic forces applied. Maybe, they should check whether there is a cell fate change during the differentiation assays in the presence of magnetic forces. Probably this is the reason why the authors want to convince us that that cardiac mesoderm differentiation pathway is enhanced as genes such as Nkx2.5, Sox17, Gata4 and Gata6 expression levels are the ones which show a greater fold-change increase in expression. The expression of other embryonic layers 'were…almost unchanged'. This is absolutely not true: all the other makers for other fates change their expression levels indicating that the other fates are affected as well. In summary, it seems that applying magnetic forces induces general differentiation towards all embryonic layers, not only cardiac mesoderm. If the authors really want to convince the readers that cardiac fate is really enhance, they should really complete a differentiation protocol to fully differentiated cardiomyocytes (either functionally or at least expression of later markers such as cardiac □-actin, Troponin T and/or Connexin-43) and show that the efficiency of differentiation of functional cardiomyocytes is increased upon application of magnetic forces.
2. In the attempt to show more evidence towards the cardiac mesoderm differentiation, the authors show Brachyury and NKX2.5 immunostainings (Fig. 4B). Brachyury and Nkx2.5 are both transcription factor with clear nuclear localization, however in the images shown, both proteins look like membrane localised. Do the authors have any explanation for this?
3. The authors conclude the manuscript stating that 'The magnetic stretcher can be used to virtually form 3D model tissues from any cells, magnetically and in situ, and then stimulate them at will, opening windows not only for biophysical studies, but also for tissue

engineering'. While it is true that the system developed by the authors (re)opens the possibility of magnetically stimulate mESCs during differentiation, how this could be applied to tissue engineering is very obscure. As the authors show themselves, as soon as they remove the magnetic force applied, the EB recover the original shape. In order to do 3D cellengineering, it is a must that cells keep the shape within the 3D structure.

4. Something that seems contradictory in the discussion and the authors do not comment on it: according to the paper cited [3], the force required to separate 2 cells is several hundred nN, and the force the authors use to deform the EB is in the order of ☐N. How do the authors explain that cells in their EB only deform but do not separate from one another?
5. There are a number of occasions where the authors do try to oversell or interpret results in the way it seems more convenient to them:

a. P. 11 (lines 222-224):'First, we can note an increase in the expression of the 2 genes (T and Nkx2.5) involved in the (cardiac) mesoderm pathway: 1.5-fold and 3-fold for T in the stretched and cyclic conditions, respectively;' The same fold-change was disregarded in the experiment shown in Fig. 2E.
b. P. 11 (lines 232-234): 'Finally, and logically, the expressions of other genes involved in the endoderm or ectoderm pathways, were either almost unchanged (Lama1, Lamb1, Lamc1, Nes and Pax6)…' the expression of these genes do change and the difference is statistically significant.

Minor comments:
1. P. 3 line 38: There is an extra ').'
2. LamC1 does show a difference when forming the magnetized EBs, (Fig.1F), what is the explanation for this?
3. Time frames in Fig 2A are not indicated
4. Some features in the figures are too small to be legible.
5. Cells in Fig 3B do not appear elongated as claimed.
6. Fig. S3: the scale bar size is not indicated.
7. There is no reference indicating the source of the information included in the diagram shown in Fig S9.

References:
1. Parsa et al.,: it is fig S8 (2015), Cell J, 17: 221-230.
2. Radzisheuskaya et al., (2013), Nature cell biology, 15: 579-590.
3. Harris et al., (2012), Proceedings of the National Academy of Sciences of the United States of America, 109: 16449-16454.

Reviewer #1

In this study, the authors investigate whether mechanical factors alone can drive differentiation of mESCs. They developed a magnetic technique to assemble single cells into 3D multicellular spheroids resembling an embryoid body formation. This technique also allows them to stretch or apply cyclic loading on the multicellular spheroids. Due to the stretching load, the EBs committed toward cardiac mesoderm lineage. I agree with the authors that making three dimensional tissue structure from individual cells and driving differentiation will advance the fields of biophysics and regenerative medicine. This is an important topic and should be of interest to a large audience. There are, however, a number of issues that should be addressed before considering the manuscript for publication.

We thank the reviewer for all the interesting comments and very pertinent suggestions made. It helped us to improve our manuscript, in a way that we hope will fulfill the reviewer's expectations.

1. The fact that "purely mechanical factors" can drive ESC differentiation, which is the main objective of this work, has been reported by many previous studies. Hence, the manuscript lacks novel findings.

Reviewer 1 is right and we are aware that previous studies reported that mechanical factors can influence stem cell differentiation. However, most of them concerned mesenchymal stem cells or endothelial progenitor cell differentiation, and were performed at the single-cell scale, on monolayers. The most common techniques then consisted in tuning the rigidity of the substrate, stretching it when deformable, or applying shear stresses generated by flow. By contrast, stimulation on 3D constructs was rarely achieved, and generally needed the support of a scaffold to arrange cells in 3D. These 3D tissue stimulations mainly focused on bone and cartilage tissue engineering. To the best of our knowledge, 3D embryonic bodies generated from embryonic stem cells were not yet stimulated and the demonstration that a mechanical stimulation could influence their differentiation in the 3D geometry has not yet been reported.
We have modified the introduction accordingly, to discuss in a more critical way the novelty of the work, while enhancing the fact that stimulation of purely cellular 3D EBs with ESC was not yet achieved:
"It is now admitted that mechanical factors can influence stem cell differentiation. However, this idea of a physical influence on differentiation emerged from studies using adult mesenchymal stem cells. Most techniques then applied on two-dimensional cell layers, and the stimulations consisted in tuning the rigidity of the substrate[7,8], stretching it when deformable, or applying shear stresses generated by flow.[9] By contrast, stimulation on three-dimensional (3D) constructs was rarely achieved, generally needed the support of a scaffold to arrange cells in 3D, and mainly focused on bone and cartilage tissue engineering.[10,11] When moving to embryonic stem cells (ESCs), works are more scarce, performed systematically in the 2D setting, and focused on the role of microenvironmental mechanical cues[12-14] or of the strain imposed by the stretching of deformable membranes supporting ESCs adhesion.[15,16] To the best of our knowledge, 3D embryonic bodies generated from ESCs have not yet been stimulated and the demonstration that a mechanical stimulation could influence their differentiation in the 3D geometry has not yet been reported."

2. What is the efficiency of EB formation in your method? Can you discuss how the magnetic attractor method is much simpler and more advantageous than hanging drop method? If controlling EB size is the only advantage over hanging drop method, they can also be achieved

by AggreWell plates or an ultra high throughput technique developed by Peter Zandstra lab (Ungrin et al., PLoS ONE, 2008). Can you compare your technique with the ones mentioned above?

We agree with the reviewer that we should have provided more figures on the comparison between our magnetic technique of EB formation, and the hanging drop method.
This now appears as Figure 3A, showing the quantification of formation efficiency, diameter, and ellipticity of the different EBs.

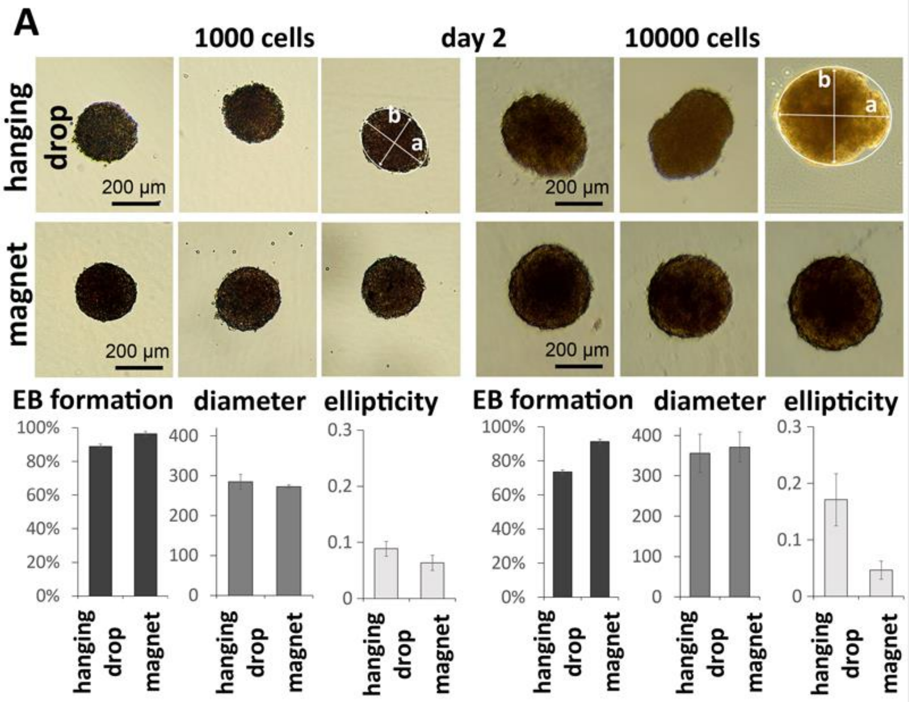

Figure 3A: Morphological comparison and formation efficiency of EBs formed by the hanging drop method, or by the 3D magnetic patterning. Top: typical images of EBs observed at day 2 after seeding (of 1000 or 10000 ESCs), either in hanging drop or over a magnetic attractor. Bottom: Quantification over 50+ EBs: Efficiency is calculated as the number of EBs actually formed over the number of hanging drops deposited or of magnetic attractors present below the dish; the diameter (expressed in µm) is the effective diameter computed from the EBs areas; and the ellipticity is defined as 1-b/a, where b is the short axis and a the long-axis of the equivalent ellipse determined by image analysis (Image J).

It demonstrates that not only the magnetic patterning increases EB circularity, but also it provides a greater efficiency of EB formation, especially in the case of 10000 cells deposited initially.
Another advantage of EB magnetic formation is the ease of implementation. Even if the throughput is not as high as with the method of Peter Zandra's lab, tenths of EB can be produced in a single small Petri dish, without the need of depositing drops one by one. More importantly, there is no need of micro-textured surfaces, and neither of a centrifuge. Furthermore, comparing the morphological characteristics of the magnetically formed EBs and the high throughput technique of Peter Zandstar's lab cannot be easily made. Indeed, in this study, EBs were compared with some generated by scraping (highly heterogeneous with poor circularity), and not by the hanging drop, and observed only 24 hours after. We can however perform the same analysis in our conditions, nevertheless 48 hours after seeding (to allow EB formation by hanging drop). The results are shown below. First they illustrate how the magnetic formation

allows a better control over size than the hanging drop (these data are now presented as a supplementary Figure S8). However, the size dispersion remains better EBs formed on microstructured wells. This is in part due to the fact that in our case, EBs are left to grow one additional day, bringing more dispersion. The corresponding article is now cited and discussed in the text (changes are copied at the end of the answer).

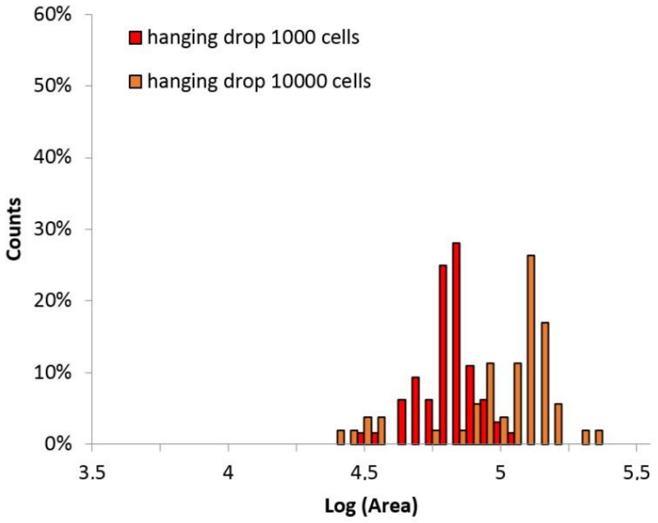

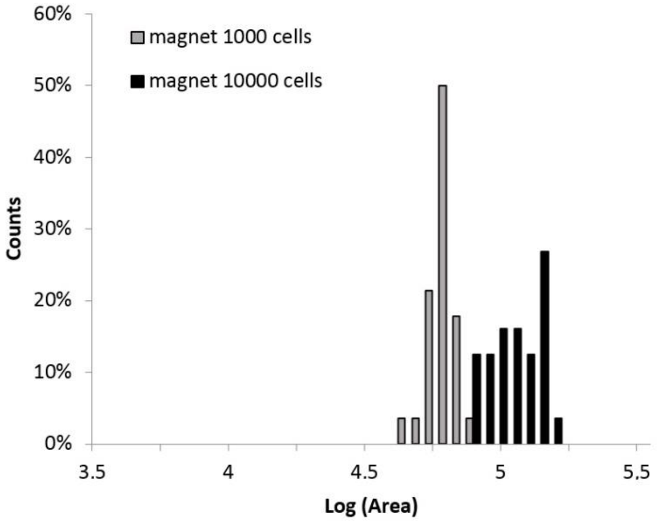

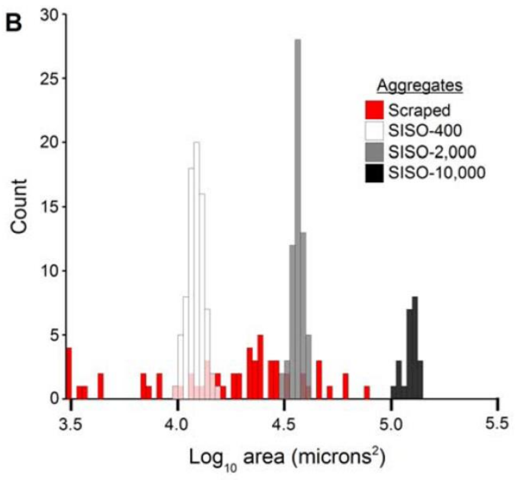

For comparison see below the graph extracted from Ungrin et al., PLoS ONE, 2008

Figure S8: Morphological analysis of EBs formed by the hanging drop (top, day 2, 1000 and 10000 cells) or magnetic method (bottom, day 2, 1000 and 10000 cells. The base-10 logarithm of sectional area is plotted on a histogram, demonstrating the increase in size control for magnetic formation over hanging drop.

We also want to emphasize that the cost of EBs magnetic formation is particularly low. Magnetic nanoparticles can be bought at prices in the range of 50€ for 1ml at 1M (see for instance http://nanocomposix.eu/products/20-nm-magnetite-nanoparticles), which can provide 1 liter of incubation medium, or equivalently about 0.1 million magnetic EBs. The magnetic pattern are even less expensive, consisting of an array of sewing needles placed in a non-magnetic metal (e.g. dural alloy) plate pierced with 0.7 mm holes (typical drill of a drilling machine), and magnetized by a permanent neodymium magnet. Details on the magnetic set-up

are now presented in supplementary Figure S6 to allow anyone to easily build his own magnetic set-up. By contrast, AggreWell plates cost about 100€ each.

All these considerations are now discussed in the text:
"***EB formation: magnetic versus hanging drop***
This system of magnetic formation allows tight control of EB size, contrary to the hanging drop method, which yields EBs of more variable size and, in some cases, no EBs at all. Figure 3A shows the percent of EB successfully formed, the EB average diameter and circularity, for magnetic EB formation or hanging drop, starting from 1000 or 10000 ESCs. Magnetic EB formation appears particularly advantageous when starting from 10000 cells, where the success rate of formation increases from 73% to 91% when using magnetic formation instead of the hanging drop approach, and EB ellipticity decreases from 0.17 to 0.04. The size control is also increased as demonstrated by a thinner distribution of EBs sectional areas in case of magnetic formation (see supplementary Figure S8)."

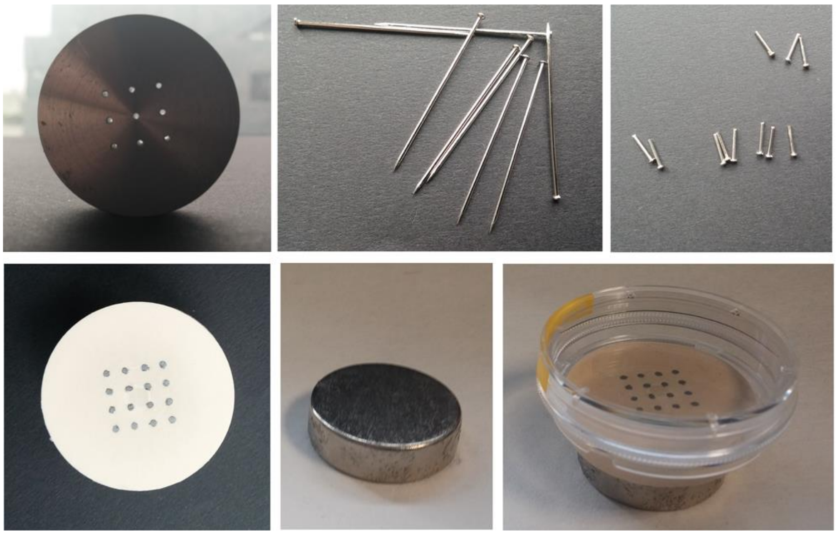

Figure S6: Magnetic device including micro-magnets to form EBs. Device fabrication is particularly simple: 1. Make holes (typically 9 or 16, arranged in a square 3-4 mm lattice) with 0.8 mm drill through aluminium cylindrical plates (Dural) 8 mm thick and 35 mm diameter to match the size of small Petri dishes; 2. Take typical sewing pin, which you insert in the holes, and cut at the plate surface (use a drilling machine to level the surface); 3. Place this magnetic pins array over a permanent magnet (typically disc neodymium magnet Ø 20 mm diameter, 8 mm height, strength about 10 kg, magnetic field created at the surface approximatively 0.4 T); 4. The device is ready to be used. Place it over a Petri dish with glass bottom, and deposit the ESCs in culture medium.

3. Were the endocytosed magnetic nanoparticles coated with targeting proteins? Or are they non-specific? Did the nanoparticles cluster inside the cells or remain dispersed after applying the magnetic field? What would be the effect of smaller forces arising from dispersed particles vs larger forces from agglomerated particles? I believe the cell will interpret applied magnetic field, i.e., the force, differently if the particles remain dispersed as opposed to agglomerated condition.

We thank the reviewer for these comments.

First, we agree that we should have added more details about the magnetic nanoparticles used. As a matter of fact, they were selected to be minimally interactive with the cells, thus with no protein coating. By contrast, they were simply coated with citrate to ensure their stability in aqueous dispersion (by electrostatic repulsion). In addition, incubation is performed without serum, to avoid opsonisation and cell activation by serum proteins accumulation on the nanoparticle surface. We also chose to work with the simplest magnetic core, composed of maghemite (oxidized iron oxide), and produced by simple co-precipitation of iron salts.
Such nanoparticles were described to enter by the endocytosis pathway, without impacting it, and concentrate in lysosomes in 2 hours. They are thus clustered in lysosomes (10^4 nanoparticles per lysosomes at maximum), as illustrated on the electron microscopy image shown in Figure 1A, and on additional images in supplementary Figure S2.
This agglomeration in lysosomes supplies each lysosome with a magnetic moment in the range of 10^{-15}-10^{-16}A.m². In the magnetic gradient used here (about 1000 T/m), the resulting magnetic force on a single lysosome is thus $1000*10^{-15}$ = 1 pN at maximum. Because the lysosomes are embedded within the viscoelastic network composing the cell interior, such a force is not enough to move the lysosomes. Indeed, the effective viscosity surrounding the lysosomes, measured using magnetic lysosomes as probes, was found to be close to 1 Pa.s. The magnetic velocity generated on a magnetic lysosome by the magnetic field is thus equal to approximately 20 nm per second (balance of the drag force 6π*effective viscosity*lysosome radius (about 0.5µm) * magnetic velocity and the magnetic force). Such a velocity is not sufficient to agglomerate the lysosomes on one side of the cell, and exert a force on the cell.
The fact that the intracellular medium is not a system at thermal equilibrium is also important. This is mainly due to the action of molecular motors which can be described with an effective temperature, almost 1000-fold the bath temperature (at low frequency) (see for instance PLoS one 2010, 5, e10046). k_BT_{eff} is thus in the order of 10^{-17} J, while the product of the force on lysosomes and the lysosomes diameter is only about 10^{-19} J, insufficient to drive an intracellular lysosomal movement, and thus a mechanosensitive response. We believe that these figures were not clear enough in the initial manuscript, because we were too concise in describing the order of magnitude of the different forces involved, and we modified the text accordingly in the discussion section:
"First, the magnitude of the magnetic (intracellular) force applied to single ESCs within the magnetic EB needs to be discussed. At 400 µm from the magnetic tip/attractor, the magnetic gradient is about 1000 T/m. This translates into a force per single ESC (loaded with 3 pg of iron, or equivalently a magnetic moment of $2x10^{-13}$ A.m²) of 200 pN approximately. This force is applied intracellularly on the nanoparticles clustered within lysosomes (at maximum 10^4 nanoparticles per lysosome), each lysosome bearing a magnetic moment in the range of 10^{-15} A.m², and thus submitted to a 1 pN force. Because lysosomes are embedded in the viscoelastic cytoplasm with effective viscosity (measured on magnetic lysosomes as probes) close to 1 Pa.s, the corresponding magnetic velocity is in the range of 0.1 µm/s, not enough to balance active motions. As a result, the lysosomes are not agglomerated one to the others (as also demonstrated on supplementary Figure 9 showing that the intracellular pattern of the magnetic lysosomes is the same with or without magnet application), and single cells does not experience an intracellular pulling force."
To confirm these numbers, we have included an additional experiment (presented as new supplementary Figure S10), where we fixed the magnetic ESCs either under the presence of the magnetic field (and corresponding magnetic gradient), or without any magnetic field, and we performed Prussian Blue staining (coloring iron in blue) in both conditions. Supplementary Figure S10 (reproduced below) shows no differences in the localization of the magnetic lysosomes in the cells, which appear dispersed, and never attracted on one side of the cell by the magnetic gradient.

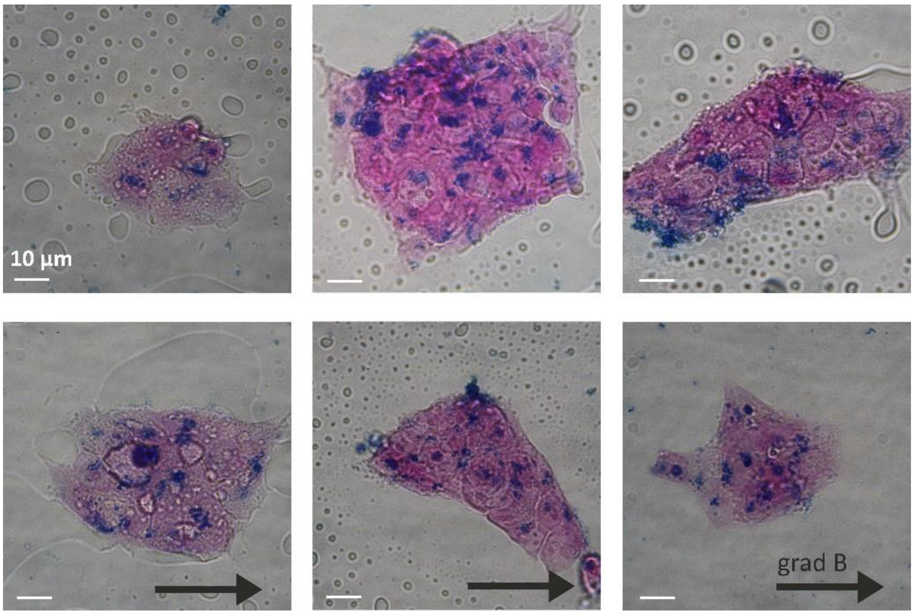

Figure S10: Observation of the magnetic lysosomes patterns inside the cells when submitted to a magnetic field gradient of 1000 T/m. The lysosomes are detected by Prussian Blue staining, which colors iron in blue. No lysosomes agglomeration can be seen in the bottom images, where the magnetic force was applied, as compared to top images, without any magnetic field application.

4. What would be the global stress or resultant strain per cell? Is it the stress or the strain that orchestrated mechanotransduction pathways during the cell differentiation? Can you provide some mechanistic insight? Please see earlier papers from the labs of Michael Sheetz, Dennis Discher, Ning Wang (del Rio et al., Science, 2009; Johnson et al., Science, 2007, Chowdhury et al, Nat Mater, 2010) for more details.

This is in continuation with the previous answer. Indeed, here, magnetism allows forming the EBs, and deforming the tissue as a whole, but not deforming single cells. We can also point out that the magnetically formed EBs, if not stimulated, present the same differentiation profile compared to the hanging drop condition. This is an interesting point regarding the fact that the cells are submitted to the same magnetic force than in the stimulated conditions.

Thus, in our set-up, magnetic forces are not used as a local mechano-transducer but are used to deform the whole aggregates. To further assess this phenomenon, we have added some live experiments of cyclic stretching. Cell membranes are stained by using a Pkh26 cell marker to follow their motion on a surface section. If the individual deformations of the cells are not accessible, we show - using a texture correlation algorith (PIV analysis) - that the strain rate distribution in the aggregates is uniform. Each cell encountered a 0.32 s-1 strain rate during stretching step and compression step. These data are shown on new Figure 4D.

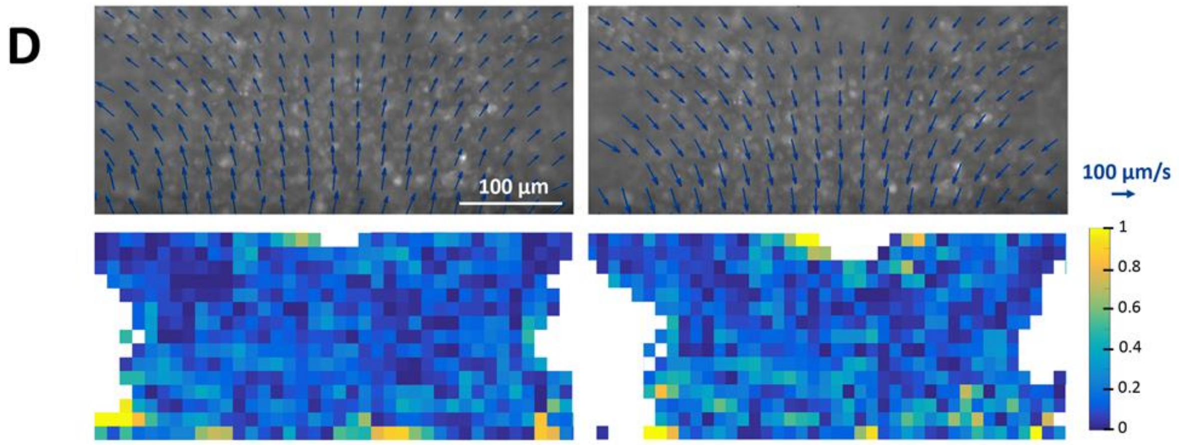

Figure 4D: Fluorescence images of membrane-stained cells in compressed (left) and stretched (right) EBs (10% imposed strain) are overlaid with velocity vectors extracted from PIV analysis (arrow bar scales for a speed of 100µm/s). Only one fourth of the vectors are represented for easy reading. The divergence of the velocity field (for stretching) or its opposite (for compression) representative for the strain rate is mapped in both cases. For compression and stretching steps the mean effective strain rate sensed by cells is calculated at 0.32± 0.08 and 0.32±0.06 s^{-1}, respectively.

It confirms that the cells do not incur a stress, but a homogeneous strain, transferred from the total strain (10%) imposed by the EB stretching.

We have thus added the following text in the Results:
"Cell movements were monitored over several stretching cycles using a membrane cell marker (Pkh26). PIV analysis provides the velocity field of the cells submitted to stretching and compression du to magnet movements (Figure 4D). No shear zones are noticeable on this figure. Moreover cells inside the EBs are submitted to a uniform strain over the whole aggregate. Indeed the divergence of the velocity field which is representative for the strain rate[35, 36] is homogenous. The mean of the effective strain is 0.32±0.08 s^{-1} for the stretching step and 0.32±0.06 s^{-1} for the compression step. Thus each cell experiences the same deformation rate."

We have also added some comments in the discussion:
"First, the magnitude of the magnetic forces applied to single ESC within the magnetic embryoid body needs to be discussed. One should then compare their intensities (in the 0.1 nN range) to the one of cell-cell adhesion. mESC-mESC adhesive forces (generated by E-cadherin/E-cadherin bonds, measured at 73 pN each) is about 9.1 × 10^5 pN.[47] This is of the same order as the force required to separate two cells (several hundred nN).[48] Thus, while the magnetic force is sufficient to attract cells and aggregate them, it is not enough to break out cell-cell bonds within an EB. This would explain why the attraction exerted by the magnetic microtip does not affect ESC differentiation: the cellular magnetic forces are far weaker than the intercellular cohesive forces, and no mechanosensitive pathways are activated by the cellular magnetic forces. Therefore it is quite difficult to compare our experiment with the mechanical stimulations and mechanotransduction evidenced in other studies.
By contrast, when we approached the second magnetic microtip towards an EB formed on the first magnetic microtip, the EB was rapidly deformed. The magnetic EB (containing N cells, each carrying a magnetic moment Mcell) must then be considered as a continuous tissue with a global magnetic moment of $N \times M_{cell}$. In the case where N = 10 000, the tissue force due to the field gradient created by the second tip is of the order of µN, which is sufficient to deform the EB. One must then consider that the stimulation is similar to a global strain, where the cells

reorient and rearrange during the stretching, and move as well together with the tissue during the cyclic stimulation. Cell movements analyzed by PIV (as shown in Figure 4D) demonstrate this vision of a tissue stimulation. Indeed, all the EB are deformed as a whole, and cells are moving altogether and deformed with the same strain, as demonstrated with no singularities in the divergence of the velocity."

And we describe this new experiment in the Material and Methods:
"Fluorescence live imaging: Cell membranes were stained with a red fluorochrome Pkh26 from Sigma. Cell stimulation with a 10% strain applied at 1 Hz was observed in situ, on living cells, by fluorescence microscopy.
Velocity mapping: The PIV analysis was computed using the Matpiv software package (a GNU public license software) for MATLAB (The MathWorks, Natick, MA).[66,67] We used 64 × 64-pixels (40x40µm) interrogation windows with 75% overlap. Calculation of the correlation between two successive subwindows was performed by fast Fourier transform (the "single" method). Aberrant vectors were filtered out from the velocity fields with a median Gaussian filter."

5. The manuscript lacks single cell analysis within the EBs and do not provide molecular mechanism of differentiation into mesoderm germ-layer.

We have modified the manuscript and included data showing single cell displacements within the EB during the stimulation. It demonstrates that the EBs experience a homogeneous strain, resembling the one applied during substrate stretching (albeit in a 2D setting). Indeed, here we do not provide molecular mechanisms, as it seems out of the scope of the present study. Nevertheless, we are now discussing in the Discussion section molecular mechanisms of differentiation into mesoderm germ-layer described in similar studies of ESCs stretching.

" It is finally important to emphasize that, herein, considering the low (0.1 pN per cell) cellular magnetic forces, the role of the mechanical stimulation is that of a global strain applied to the EB, and resembles the situation of a mechanical stretching on a 2D deformable substrate. In such cases, it was demonstrated that cyclic stimulation was sufficient to drive ESC differentiation into cardiac mesoderm. The exact molecular mechanism whereby stem cells translate external forces to mesodermal differentiation is still under investigation. Several mechanisms are proposed.[15] Many studies demonstrated that the production of intracellular reactive oxygen species initiates a cardiovascular differentiation program via induction of several signaling pathways such as PI3K/Akt, ERK1/2, JNK, and p38.[53, 54] In addition, integrin-mediated modification of cell signaling pathways such as PI3K/Akt and GSK-3ß has a particular role in cardiac differentiation.[54, 55] It is known that mechanical stimuli such as stretch and shear stress can activate these signaling pathways.[56-58] Moreover, Banerjee et al[59] demonstrated that mechanical strain is sufficient to regulate Tgf-β signaling and Tgf-β-dependent gene expression. Extracellular matrix proteins (ECM) are also considered as mediators of environmental forces to control cell differentiation. Upon cell compaction during tissue formation, the cells secrete more and more ECM components and they sense external forces either through cell-cell and cell-ECM interactions, mechanosensitive ion channels or by directly sensing the force by wave propagation throughout the cell and toward the nucleus.[60] For example, heparin sulfate proteoglycans have been observed to play such a role in ESCs. Toh and Voldman demonstrated that mouse ESCs also mechanically sense shear stress via heparin sulfate proteoglycans to modulate Fgf5 expression.[61] Cell stretching promotes also modulation of the cell membrane and orientation of actin filaments, which facilitates the

connections made between cells that are necessary to promote intracellular communication.[62] Cell-cell contacts through cadherins adhesion[63, 64] enhance this process by alpha-catenin and vinculin recruitment and are involved in regulation of the Oct4-Nanog-Sox2 circuitry.[65] ”
.

Minor:

1. What is the reason for using RPLP0 as normalizing gene in your qPCR experiments? Why not the commons normalizing genes such as GAPDH or EF1a?

The commonly used reference gene GAPDH is neither the most stable nor constantly expressed in all the tissues (Kouadjo KE et al, 2007, BMC Genomics, 8: 127; Thomas KC et al, 2014, PLos One, 9 (2): e88653). Ribosomal proteins are identified as the most constitutively and constantly expressed (de Jonge HJ et al, 2007, PLos one, 2(9): e898). In our experiments, the RPLP0 gene appears as a stable housekeeping gene in which the expression between the different conditions (static, hanging drop, stretched and stimulated) was unchanged : CT = 26.85 ± 0.4; 26.94 ± 0.4; 26.21 ± .43 and 26.84 ± 0.43 respectively, with 136 ± 3.5 ng cDNAs per reaction for each condition.

2. Overall, the introduction section lacks citations of relevant previous works. For example, in line 43-45, the cited reference on the effect of substrate rigidity on cell differentiation do not refer to Engler et al., Cell, 2006 paper. In addition, hESC related references do not apply to this study.

We totally agree that the article published in Cell in 2006 marked a turning point, showing that the relative rigidity of a substrate can orient the differentiation of mesenchymal stem cells (MSC), without the need for specific growth factors. This programming induced by local elasticity sensed by cells really pioneered the next studies. We chose to cite more recent ones, but we agree that we should have acknowledged the first one. It is now done in the revised manuscript.

Reviewer #2

In this manuscript, Du et al devise and test a novel method to generate and differentiate embryoid bodies from murine embryonic stem cells. This method utilises iron nanoparticles, which can be engulfed by the cells, allowing the cells to be mobilised using magnets. The authors first optimise the concentration of nanoparticles and duration of application to allow sufficient uptake of the particles but without causing overt toxicity. They test the capacity of labelled cells to aggregate into EBs and observe no overt difference compared with unlabelled ESCs using the hanging drop method. They then test the capacity of the nanoparticle-laden cells to aggregate in response to a magnetic stimulation, and thus efficiently generate EBs, which exhibit the same differentiation potential as unlabelled cells. Using movable magnets, they then apply forces to stretch the EBs (by single pulse or cyclic stimuli to resemble functioning cardiac muscle) and observe their differentiation potential compared with unstretched EBs. They show enhanced expression of a panel of differentiation markers using qRT-PCR and some confirmatory immunohistochemstry of Brachyury and Nkx2.2. The consequences of cell stretching on differentiation have been previously reported, but not using ESCs, so the demonstration of the effect in this system that the authors equate to embryonic development is novel.

However, in order for the technology to be adopted by the pluripotent stem cell field a more directed differentiation approach would need to be demonstrated as well as evidence that the engulfment of iron nanoparticles is consistent with longer term stable culture.

We thank the reviewer for these very constructive comments.

Concerning the first part, we have added new experiments to test over longer periods the differentiation profile of the stimulated EBs, and explore whether a more directed differentiation towards cardiomyocytes was achieved. EBs were left maturing up to day 10 after LIF removal, and specific cardiac markers were tested. The results in gene expression are now shown as new Figure 5C, and reproduced below. Remarkably, cardiomyocyte specific cytoskeletal *Tnnt2* (Cardiac Troponin-T), *Myh6* (Myosin heavy chain, α isoform) and *Myl2* (Myosin regulatory light chain 2) genes were significantly overexpressed for both the stretched and the cyclic conditions, and overexpression was more important for the cyclic setting. *Actc1* (Cardiac α-actin) was significantly overexpressed only for the cyclic condition, but upregulation was low in all conditions, suggesting that this marker is too late for an expression at day 10.

C

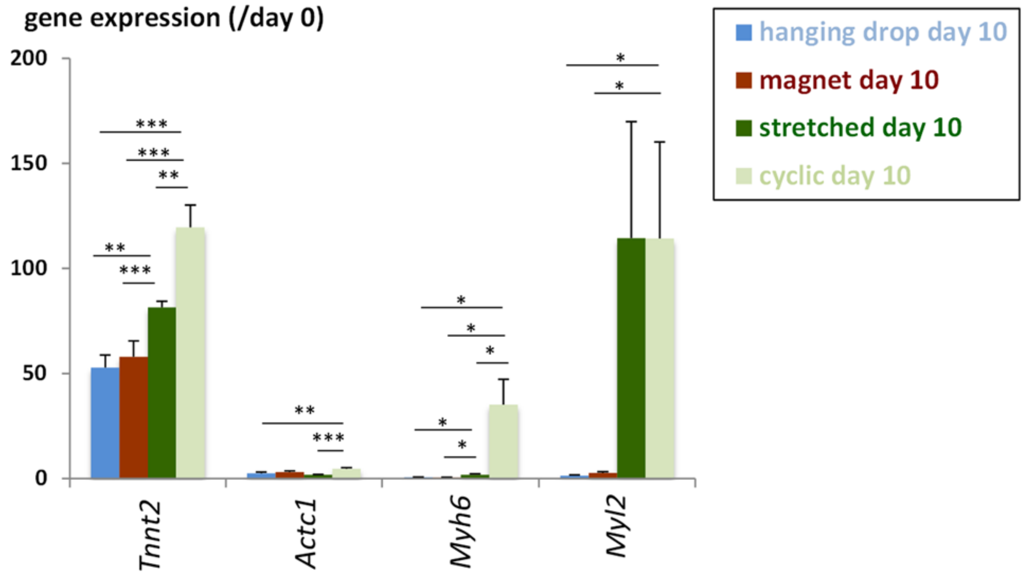

Figure 5C: Gene expression at longer maturation times (day 10) for specific cardiac markers cardiac troponin T (*Tnnt2*), cardiac α-actin (*Actc1*), α myosin heavy chain (*Myh6*) and myosin regulatory light chain 2 (*Myl2*). All EBs were obtained from 10000 ESCs. For the hanging drop formation (blue), ESCs were not labelled with the magnetic nanoparticles. For the three other conditions, ESCs were magnetic (3 pg of iron per cell): EB formation by magnet with no further stimulation (dark red), stretched stimulation (dark green) and cyclic stimulation (light green). mRNA levels are shown relative to control (day 0, defined as 1), and normalized to reference gene RPLP0.

These new results are now commented in the text, Results and Discussion sections:
"Finally, in order to detect if a commitment towards the cardiac lineage was really enhanced, we analyzed EB at longer maturation times (day 10), and quantified by qPCR the expression of transcripts encoding for specific cardiomyocyte markers. We tested troponin T (*Tnnt2*), involved in cardiomyocyte contraction, cardiac α-actin (*Actc1*), the cardiac cytoskeletal marker, α-myosin heavy chain (*Myh6*), involved in contraction and considered as a maturation marker, and myosin regulatory light chain 2 (*Myl2*), involved in the regulation of myosin ATPase activity and known as a ventricular cardiomyocyte marker (Figure 5C).
The hanging drop and magnet conditions of EBs formation led to similar results for all genes and, overall, the efficiency of differentiation towards functional cardiomyocytes was increased upon application of stretched and cyclic stimulations. Compared to the magnet condition, *Tnnt2*, *Myh-6* and *Myl2* genes were overexpressed for the stretched condition, and this upregulation was higher following cyclic stimulation for *Tnnt2* and *Myh-6*. The impact on cardiac α-actin was less pronounced, with a significant upregulation only for the cyclic condition. This protein is, among others involved in the left ventricular compaction,[37] and probably expressed later."

Concerning the second part of the reviewer comment, we have monitored the magnetism of EB over 7 days, which is a direct indicator of the nanoparticle integrity and presence within the cells over long culture times.
This experiment is now shown as a new Figure 2E (copied below). It consists of measuring the EB magnetic migration towards a magnet at different growth times, after the initial incorporation of the magnetic nanoparticles.

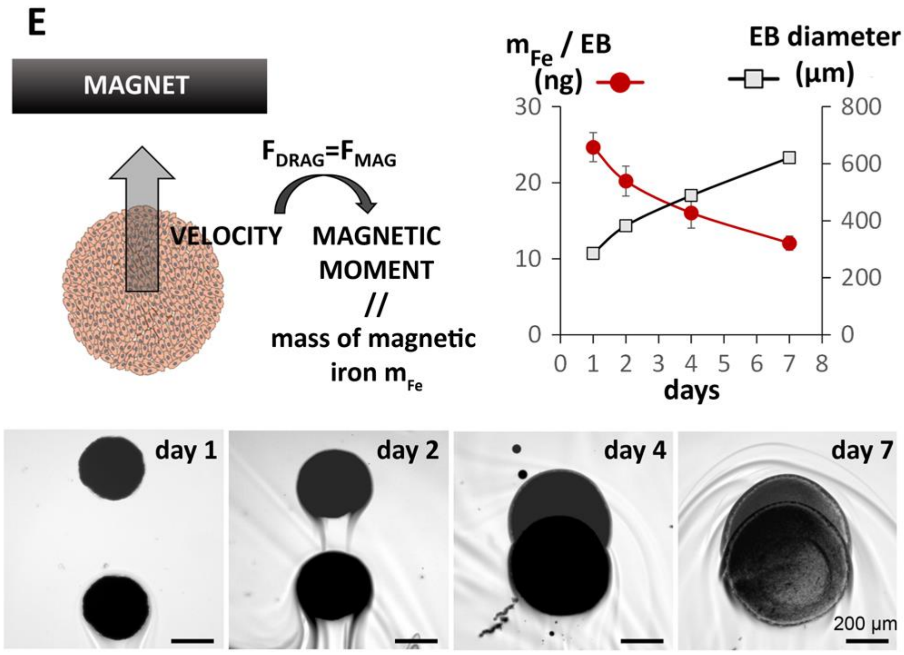

Figure 2E: Monitoring of EBs magnetism of EB over 7 days after nanoparticles cellular incorporation, and EB formation (day 0). It consists of tracking the EB magnetic migration towards a magnet (scheme shown on top left), and measuring the corresponding velocity, which translates into the EB magnetic moment (proportional to the mass of iron per EB) by balancing the viscous drag and the magnetic force. Typical migrations are shown (bottom) for the different times (days 1, 2, 4 and 7), corresponding to the superimposition of two images at 3 seconds interval. The mass of iron (circles) and the EBs diameters (squares), averaged over 8 different EBs, were then plotted as a function of time (top right).

The following texts have been added in the manuscript:
- In the Methods section:

"**EB magnetophoresis for long-term monitoring of nanoparticles fate**

To measure the magnetic moment (M) of the EBs, single EBs were immersed at each different time point after formation (days 1, 2, 4, and 7, n>8 for each condition) in a glycerol solution (80%, room temperature 23-24 °C, viscosity η=0.05 Pa.s) submitted to a magnetic field gradient (B= 150 mT, gradB=17.5T/m) generated by a permanent magnet (cylinder 25 mm in diameter, 10 mm height). Each EB thus experiences a magnetic velocity v_{mag} towards the magnet, by balancing the magnetic force MgradB, and the Stokes drag force $6\pi\eta R v_{mag}$, where R is the EB radius. EB migration was video-monitored every 0.1 s (4X objective, Leica DMIRB microscope). The magnetic moment calculated (in A.m², at 150 mT) can be converted to grams of (magnetic) iron (68 emu/g at 150 mT, 1A.m²=10^3emu)."

- In the Results section:

"One essential question remains that of the fate of the nanoparticles once internalized within ESCs. Or alternatively, will the EB stay magnetic over long-term culture conditions? To address this issue, we monitored EBs' magnetism (initially 10000 cells) at different times after EB formation, by magnetophoresis (Figure 2E). Briefly, it consists of tracking the EB magnetic mobility when submitted to a homogeneous magnetic field gradient created by a permanent magnet. The magnetic velocity can then be directly converted into the EB magnetic moment, or alternatively the amount of nanoparticles (expressed in mass of iron) contained within the EB. At day1 after formation, each EB contains in average 25 ng of iron, consistent with the initial iron load per single ESC of about 3 pg. This amount progressively decreases during EB

growth, attaining about half its initial value at day 7. This is due to the lysosomal degradation of the nanoparticles, as recently evidenced in MSC spheroids.[35] While degradation is beneficial for long-term ability of magnetically-labelled tissue to get rid of the initial nanoparticles, the fact that at day 7, EBs still retain half their magnetization is also beneficial for multiple magnetic stimulations before tissue maturation."

Although the manufacture and initial validation of the system are interesting, the application to regenerative medicine are not obvious.

The reviewer is totally right to stipulate that regenerative medicine application is not the scope of this manuscript, focused on EB stimulation, and its role on the differentiation profile. When we wrote that the system would have some potential for tissue engineering, we had in mind applications such as cell alignment (i.e. muscle cells) in a 3D cellular construct, for muscle tissue engineering. Anyway, as said, it is not the subject of this study, and we have removed the two sentences opening up the device to tissue engineering as such applications are still too premature.

Reviewer #3

What are the major claims of the paper?

In ‘An all-in-one magnetic tissue stretcher for forming and stimulating in situ embryoid bodies: towards remote mechanical control of stem cell differentiation’, the authors apply and optimize mouse embryonic stem cells (mESCs) magnetic labelling and assess its impact, together with magnetic stretching, on embryoid body (EB) formation and differentiation.
More specifically, the authors address three questions/issues:
1. ‘The impact of nanoparticle internalization on the cell phenotype, and particularly differentiation capacity’ in mESCs.
2. ‘Whether 3D magnetic “printing” of ESCs could be equivalent’ and ‘simpler’ to EB formation using the hanging drop method.
3. ‘Could magnetic forces alone drive stem cells differentiation within a magnetically formed 3D model tissue?’ According to the manuscript, it is ‘evidenced the impact of purely mechanical stimulation on EB differentiation’ as the magnetically formed EBs (=magnetic-EBs) seem to have enhanced differentiation toward cardiac fate.
The authors address these questions using a combination of qPCR and immunofluorescence analysis of specific genes (pluripotency and differentiation, depending on the experiment), cell viability tests and visual inspection of the generated EBs. In summary, the authors incorporate the magnetic nanoparticles in mESCs, generate EBs which can be deformed using magnetic forces which seems to enhance differentiation towards all embryonic layers, which is not *per se* very interesting or attractive. Although the study presented here shows some novelty and is of potential interest, overall, the work presented is still in a preliminary state with no clear major contribution to the field. A number of issues need to be addressed before I can recommend its publication.

We thank the reviewer for all the contributions he made to the manuscript by highlighting very interesting and relevant shortcomings. It helped us improving our manuscript, through the addition of several new experiments, in a way that we hope will fulfill the reviewer’s expectations.

Specific major comments:

For question No1,
1. This part of the study is not novel as Parsa *et al.1* did also used iron oxide nanoparticles and analysed how they affected mESCs viability, pluripotency and differentiation using EB formation. Magnetic nanoparticles have been used multiple times in the literature in the context of adult stem cells, as indicated by the authors citing some of the relevant papers.
The synthesis of the nanoparticles used in the present manuscript is not detailed enough to distinguish how different these nanoparticles are form others used in the literature.

The reviewer is right to stipulate that magnetic nanoparticles have been already used to label adult stem cells, generally in order to provide the cells with MRI contrast for cell imaging, and their impact on differentiation was thoroughly documented. By contrast, we could only find one study investigating the impact of magnetic nanoparticles on ESC differentiation, and this study tested only cardiomyogenesis. The study by Parsa et al. is another one, but it only tested the directed differentiation towards hematopoietic stem cells, and only assessed surface phenotypic markers by flow cytometry, but not gene expression. The impact on the whole ESC differentiation profile, towards the three embryonic layers, has still been untested. Besides, the study of Parsa et al. used large commercial nanoparticles (Endorem), which need a transfection

agent (protamine sulfate) to penetrate the cells, which, by itself, is more prone to change the cell phenotype than the nanoparticles themselves. We cited this article, and commented it briefly:

"Only few studies have investigated the impact of magnetic nanoparticles on ESCs. One reported that cardiomyogenesis was unaffected,[34] another that the self-renewal ability or surface phenotypic markers expressed after forced differentiation into hematopoietic cells by a cytokine cocktail were unchanged.[35] To the best of our knowledge, the impact of magnetic nanoparticles on the whole ESC differentiation profile, with no biochemical triggers, is still unknown."

We also agree that we should have provided more details on the nanoparticles we used.
They were selected to be minimally interactive with the cells, thus with neither protein coating (only citrate absorption to ensure aqueous stability), nor need for a transfection agent for them to penetrate the cells. Besides, incubation is performed without serum, to avoid opsonisation, and cells activation by serum proteins accumulation on the nanoparticle surface. We also chose to work with the simplest magnetic core, composed of maghemite (oxidized iron oxide), and produced by simple co-precipitation of iron salts. While the synthesis is particularly simple, it is also possible to buy them at reasonable prices (see for instance nanoComposix nanoparticles).

The material and methods section has been modified accordingly:
"Iron oxide nanoparticles were synthetized by alkaline coprecipitation of $FeCl_2$ (0.9 mol) and $FeCl_3$ (1.5 mol) salts. The nanoparticles were then oxidized into maghemite with 1.3 mol of iron nitrate under boiling. After magnetic decantation, the maghemite nanoparticles were heated at 80°C for 30 min in water, then supplemented with sodium citrate (70 g) to promote absorption of citrate anions onto their surface (to ensure electrostatic stabilization in aqueous solution) before precipitation in acetone at 25°C and resuspension in water. The resulting nanoparticles were 8 nm in diameter, with polydispersity index of 35%."

2. The authors show that, if they incubate the cells for 30min in 2mM [Fe], the used nanoparticles do not affect mESC pluripotency following the expression of NANOG and SOX2.
However there is an increase (statistically significant) of OCT4. How do the authors explain this? Does this increase of OCT4 have an impact on mESC behaviour? It has been previously shown that specific, tightly regulated levels of OCT4 are key for pluripotency2, the increase shown here can be potentially affecting the magnetized mESCs.

We agree with the reviewer comment that an upregulation of Oct4 could have a key impact for pluripotency. However, we emphasize that here, the increase in Oct4 for this specific condition (incubation 30 min at 2 mM) was only 1.3-fold, and that for higher doses (incubations 2 hours at 2 mM and 5 mM concentration) we failed to record such an increase in Oct4 expression. We thus believe that this increase should not be considered significant. Besides, differences less than 1.5-fold in q-PCR should be treated with caution.
We are now presenting the data in a larger scale and have added a small comment in the caption of the corresponding Figure 1E.

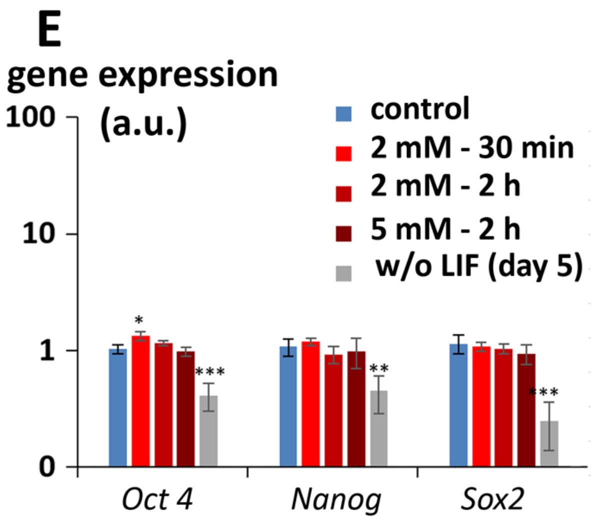

Figure 1E: One can note that only one condition led to a significant upregulation (*Oct4* – incubation at 2 mM for 30 min). However the gene was upregulated less than 1.5-fold (1.3-fold exactly). Besides, higher doses (2 hours incubation at 2 and 5 mM) provide the same *Oct4* expression as the control.

3. On a related topic to point 2, the authors do not show what the long term effect of the nanoparticles in the mESCs is.
Also the following should be tested:
-for how long are the nanoparticles maintained in the cells once they've been endocytosed? If the authors passage the cells, after how many passages do these particles disappear? This is an important issue if the application of this technique will eventually be to do *in vivo* work.
-the cell viability tests shown are performed after 30min or 2-4h after incubating the cells with the nanoparticles, if the incubation is done for 30min and then viability tests are done a few days after, do the magnetised cells show a decreased viability?

Concerning the long term effect of the nanoparticles, first, we must say that at days 5 and 7 of EBs maturation, we detected no changes in gene expression for magnetic cell compared to control cells (Figure 1F). Besides, we have now performed new experiments at longer maturation times (day 10), and again, we have not seen differences in gene expression (here for the cardiac markers) for EBs formed with unlabeled cells, or cells having nanoparticles inside (see new Figure 5C).

Concerning the long-term fate of nanoparticles within the cells, we have performed a new series of experiments to monitor the magnetism of EB over 7 days, which is a direct indicator of the nanoparticle integrity and presence within the cells over long culture times. It demonstrates that EBs are still magnetic after one week of tissue maturation.
This experiment is now shown as a new Figure 2E (copied below). It consists of measuring the EB magnetic migration towards a magnet at different growth times, after the initial incorporation of the magnetic nanoparticles.

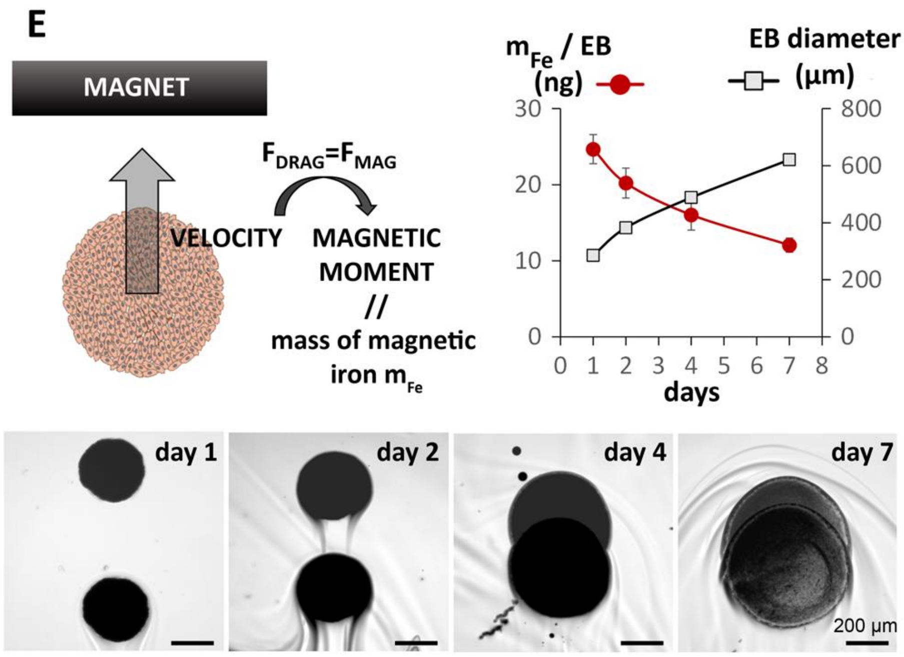

Figure 2E: Monitoring of EBs magnetism of EB over 7 days after nanoparticles cellular incorporation, and EB formation (day 0). It consists of tracking the EB magnetic migration towards a magnet (scheme shown on top left), and measuring the corresponding velocity, which translates into the EB magnetic moment (proportional to the mass of iron per EB) by balancing the viscous drag and the magnetic force. Typical migrations are shown (bottom) for the different times (days 1, 2, 4 and 7), corresponding to the superimposition of two images at 3 seconds interval. The mass of iron (circles) and the EBs diameters (squares), averaged over 8 different EBs, were then plotted as a function of time (top right).

The following texts have been added in the manuscript:

- In the Methods section:

"**EB magnetophoresis for long-term monitoring of nanoparticles fate**

To measure the magnetic moment (M) of the EBs, single EBs were immersed at each different time point after formation (days 1, 2, 4, and 7, n>8 for each condition) in a glycerol solution (80%, room temperature 23-24 °C, viscosity η=0.05 Pa.s) submitted to a magnetic field gradient (B= 150 mT, gradB=17.5T/m) generated by a permanent magnet (cylinder 25 mm in diameter, 10 mm height). Each EB thus experiences a magnetic velocity v_{mag} towards the magnet, by balancing the magnetic force MgradB, and the Stokes drag force $6\pi\eta R v_{mag}$, where R is the EB radius. EB migration was video-monitored every 0.1 s (4X objective, Leica DMIRB microscope). The magnetic moment calculated (in A.m², at 150 mT) can be converted to grams of (magnetic) iron (68 emu/g at 150 mT, 1A.m²=10^3emu)."

- In the Results section:

"One essential question remains that of the fate of the nanoparticles once internalized within ESCs. Or alternatively, will the EB stay magnetic over long-term culture conditions? To address this issue, we monitored EBs' magnetism (initially 10000 cells) at different times after EB formation, by magnetophoresis (Figure 2E). Briefly, it consists of tracking the EB magnetic mobility when submitted to a homogeneous magnetic field gradient created by a permanent magnet. The magnetic velocity can then be directly converted into the EB magnetic moment, or alternatively the amount of nanoparticles (expressed in mass of iron) contained within the EB. At day1 after formation, each EB contains on average 25 ng of iron, consistent with the initial iron load per single ESC of about 3 pg. This amount progressively decreases during EB growth, attaining about half its initial value at day 7. This is due to the lysosomal degradation

of the nanoparticles, as recently evidenced in MSC spheroids.[35] While the degradation is beneficial for long-term ability of magnetically-labelled tissue to get rid of the initial nanoparticles, the fact that at day 7, EBs still retain half their magnetization is also beneficial for multiple magnetic stimulations before tissue maturation."

Finally, concerning cell viabilities a few days after the magnetic labeling, they are not affected by the intracellular presence of the nanoparticles, as shown in new supplementary Figure S3.

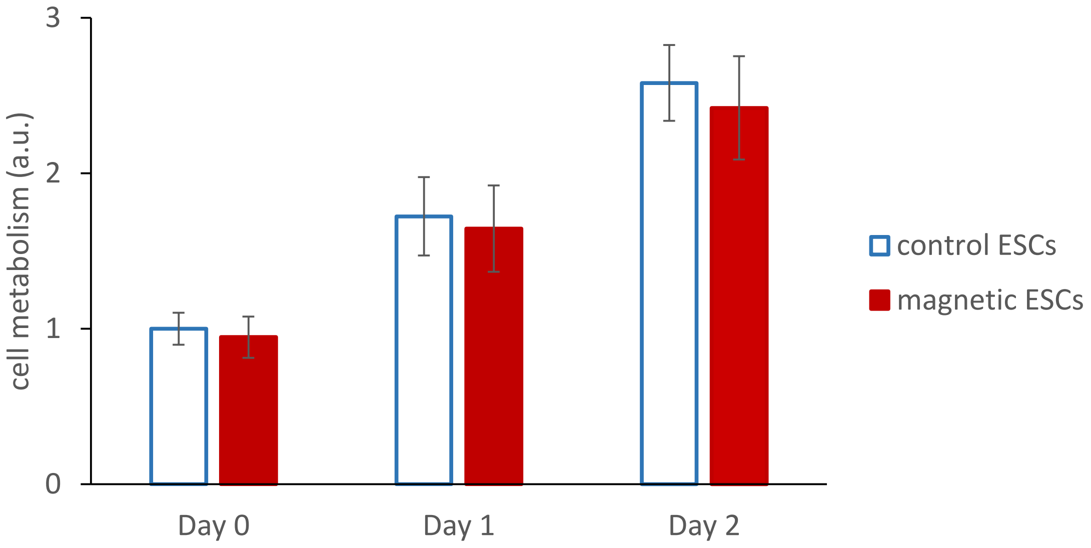

Figure S3: Cell metabolism measured by Alamar Blue assay at different times after incubation with magnetic nanoparticles (cellular uptake of 3 pg of iron per cell): first day (day 0, two hours after incubation; 24 hours after (day 1); and 48 hours after (day 2).

For issue/question 2, the authors show that their method results in equivalent EBs (differentiation marker expression profile shown in Fig. 2) as shown in *1*. They propose that generating EBs using magnetic forces is a simpler method as the magnetic-EBs are less variable in size and more homogeneous than the traditional hanging drop-EBs. However, besides a couple of images, this has not been quantified. On this, it would be advisable that the authors undertake a detailed comparison between both methods showing measurements of EB numer, size and sphericity to show that magnetic-EB are more efficiently formed.

We agree with the reviewer that we should have provided more figures on the comparison between our magnetic technique of EB formation, and the hanging drop method.
This now appears as Figure 3A, showing the quantification of formation efficiency, diameter, and ellipticity of the different EBs.

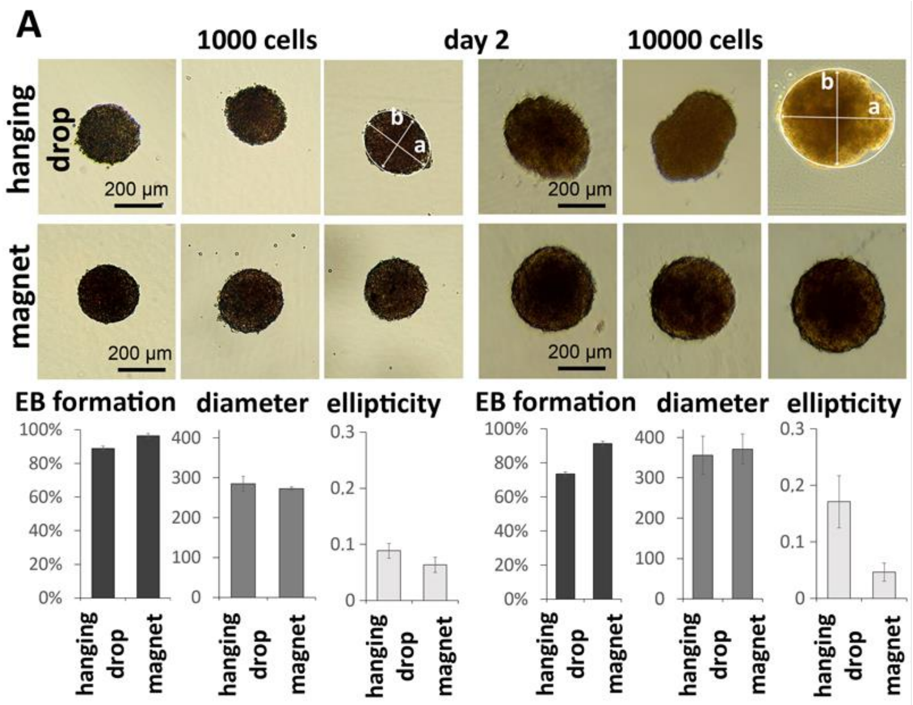

Figure 3A: Morphological comparison and formation efficiency of EBs formed by the hanging drop method, or by the 3D magnetic patterning. Top: typical images of EBs observed at day 2 after seeding (of 1000 or 10000 ESCs), either in hanging drop or over a magnetic attractor. Bottom: Quantification over 50 EBs: Efficiency is calculated as the number of EBs actually formed over the number of hanging drops deposited or of magnetic attractors present below the dish; the diameter (expressed in µm) is the effective diameter computed from the EBs areas; and the ellipticity is defined as 1-b/a, where b is the short axis and a the long-axis of the equivalent ellipse determined by image analysis (Image J).

It demonstrates that not only the magnetic patterning increases EB circularity, but also it provides a greater efficiency of EB formation, especially in the case of 10000 cells deposited initially.

These new experiments are described in the Results section:
"***EB formation: magnetic versus hanging drop***
This system of magnetic formation allows tight control of EB size, contrary to the hanging drop method, which yields EBs of more variable size and, in some cases, no EBs at all. Figure 3A shows the percent of EB successfully formed, the EB average diameter and circularity, for magnetic EB formation or hanging drop, starting from 1000 or 10000 ESCs. Magnetic EB formation appears particularly advantageous when starting from 10000 cells, where the success rate of formation increases from 73% to 91% when using magnetic formation instead of the hanging drop approach, and EB ellipticity decreases from 0.17 to 0.04. The size control is also increased as demonstrated by a thinner distribution of EBs sectional areas in case of magnetic formation (see supplementary Figure S6)."

Anyway the proposed method is not simpler as it required the use of custom made devices which are not at hand to other researchers.
We agree with the reviewer that devices were custom made. However, we must emphasize that their fabrication is quite simple, accessible to all. It consists of an array of sewing needles placed in a non-magnetic metal (e.g. dural alloy) plate pierced with 0.7 mm holes (typical drill of a

drilling machine), and magnetized by a permanent neodymium magnet. Details on the magnetic set-up are now presented in supplementary Figure S6. It shows how simple are the devices, and how user-friendly and widely accessible is their fabrication.

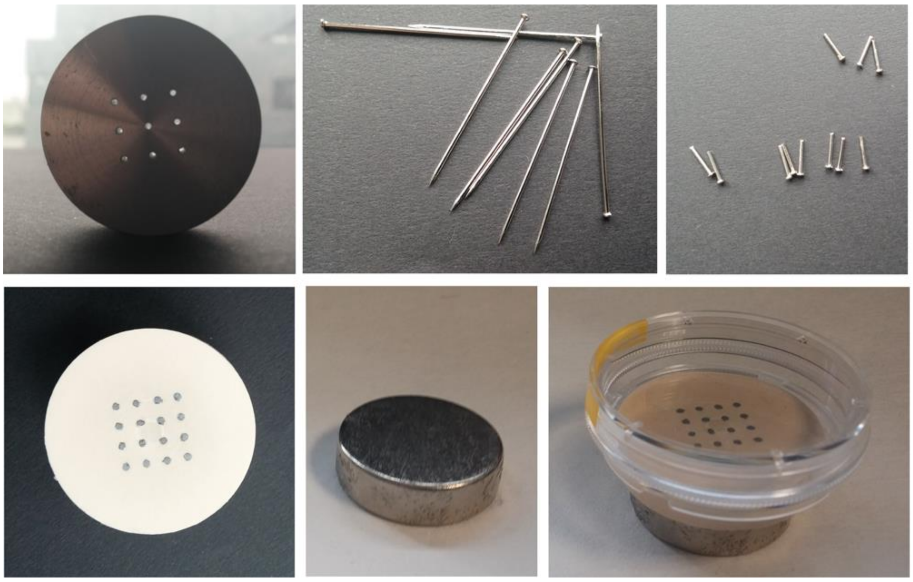

Figure S6: Magnetic device including micro-magnets to form EBs. Device fabrication is particularly simple: 1. Make holes (typically 9 or 16, arranged in a square 3-4 mm lattice) with 0.8 mm drill through aluminium cylindrical plates (Dural) 8 mm thick and 35 mm diameter to match the size of small Petri dishes; 2. Take typical sewing pin, which you insert in the holes, and cut at the plate surface (use a drilling machine to level the surface); 3. Place this magnetic pins array over a permanent magnet (typically disc neodymium magnet Ø 20 mm diameter, 8 mm height, strength about 10 kg, magnetic field created at the surface approximatively 0.4 T) 4. The device is ready to be used. Place it over a Petri dish with glass bottom, and deposit the ESCs in culture medium.

On issue/question 3, and this is the issue that the authors really try to oversell**:**
1. They show that the magnetically formed EBs (=magnetic-EBs) do differentiate in an equivalent manner to the hanging drop-formed EBs (albeit with different levels of expression of several markers, see below). Contrary to what the authors claim, this does not show that mechanical stimulation does drive differentiation: in magnetic-EBs with continuous or cyclic stretching differentiation occurs following the same pattern of gene expression as in otherwise formed EBs, albeit with enhanced expression of all embryonic layers. This indicates that magnetic forces alone do not drive differentiation, if any, they enhance differentiation.

Here, we believe that one misunderstanding comes from the way we presented our data. Indeed, we have now normalized gene expression to that of undifferentiated ESC by taking 1 as the reference value on the horizontal axis. It then appears clearly (Figure 4C) that markers for the endoderm and ectoderm layers are not overexpressed, and are even sometimes downregulated, compared to undifferentiated ESCs.

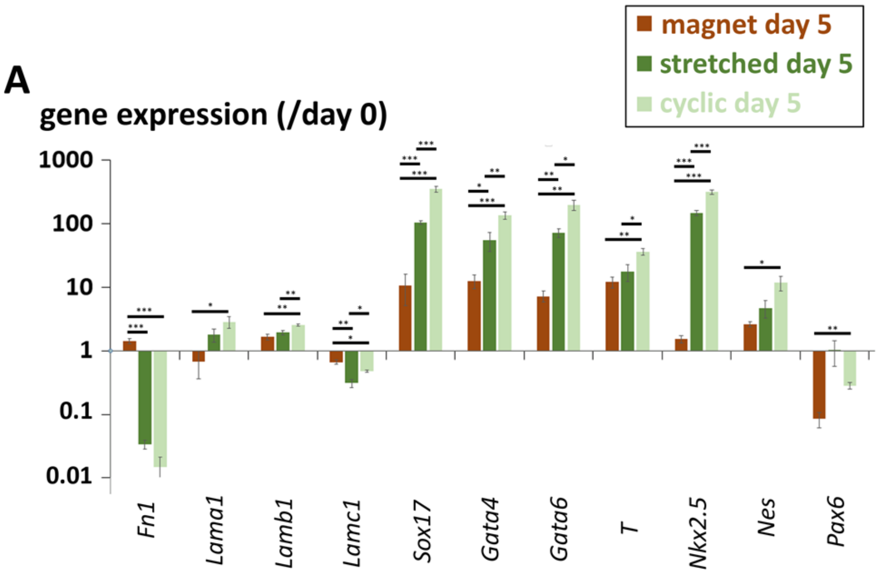

Figure 4C

If the authors want to claim this, they should show that in the absence of differentiation cues, magnetic forces do drive differentiation. In the presented experiment, EBs are formed which in itself is a source of differentiation cues, therefore this is not a suitable model to address whether magnetic forces do drive differentiation. This is not something easily addressed experimentally, as the suitable model would require absolutely no differentiation cue but the magnetic forces applied. Maybe, they should check whether there is a cell fate change during the differentiation assays in the presence of magnetic forces.

We agree that EBs formation by itself drives the differentiation towards the mesoderm layer, as it appears also in Figure 3D (copied below). However, stimulation further increases the expression of cardiac mesoderm markers.
We also want to emphasize that it is the originality of this work to apply a stimulation to an EB, to more closely mimic the in vivo situation, in contrast to what has usually been done in adherent ESCs.
We have commented this in the revised manuscript:

"We acknowledge that the initial step of EB formation by itself drives differentiation towards the mesoderm layers (see gene expression of the three layers at day 5 in Figure 2B). Still, the difference between cardiac mesoderm and endoderm/ectoderm markers is much more accentuated when stimulation is applied (Figure 4C). The stimulation by itself thus does not drive the differentiation towards the cardiac mesoderm but it strongly enhanced it."

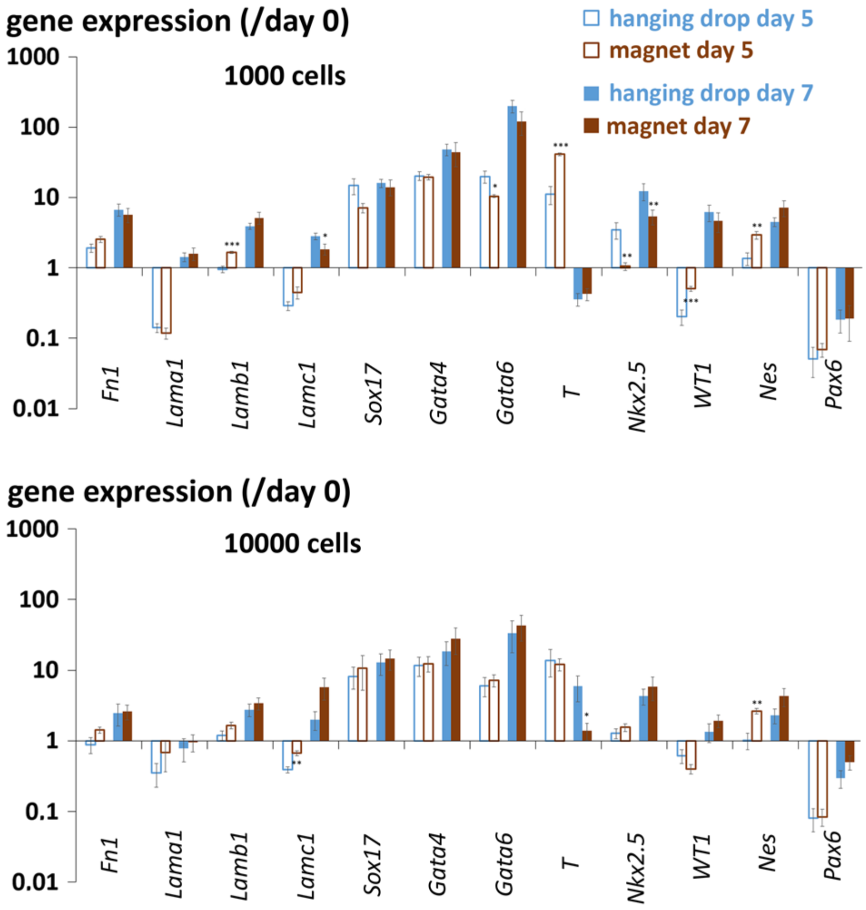

Figure 3B

Probably this is the reason why the authors want to convince us that that cardiac mesoderm differentiation pathway is enhanced as genes such as *Nkx2.5, Sox17, Gata4* and *Gata6* expression levels are the ones which show a greater fold-change increase in expression. The expression of other embryonic layers 'were…almost unchanged'. This is absolutely not true: all the other makers for other fates change their expression levels indicating that the other fates are affected as well.
In summary, it seems that applying magnetic forces induces general differentiation towards all embryonic layers, not only cardiac mesoderm.

We acknowledge that magnetic stimulation induced a global change in the gene expression profile of the ESC. However, genes committed to the cardiac mesoderm were differentially upregulated to a greater extent while the expression of endodermal/ectodermal genes was often not substantially different from that of the undifferentiated starting material.

If the authors really want to convince the readers that cardiac fate is really enhanced, they should really complete a differentiation protocol to fully differentiated cardiomyocytes (either functionally or at least expression of later markers such as cardiac α-actin, Troponin T and/or Connexin-43) and show that the efficiency of differentiation of functional cardiomyocytes is increased upon application of magnetic forces.

We have added new experiments to test over longer periods the differentiation profile of the stimulated EBs, and explore whether a more directed differentiation towards cardiomyocytes was achieved. EBs were left maturing up to day 10 after LIF removal, and specific cardiac markers were tested. The results in gene expression are now shown as new Figure 5C, and reproduced below. Cardiomyocyte specific cytoskeletal *Tnnt2* (Cardiac Troponin-T), *Myh6* (Myosin heavy chain, α isoform) and *Myl2* (Myosin regulatory light chain 2) genes were significantly overexpressed for both the stretched and the cyclic conditions, and overexpression was more important for the cyclic setting. *Actc1* (Cardiac α-actin) was significantly overexpressed only for the cyclic condition, but upregulation was low in all conditions, suggesting that this marker is too late for an expression at day 10.

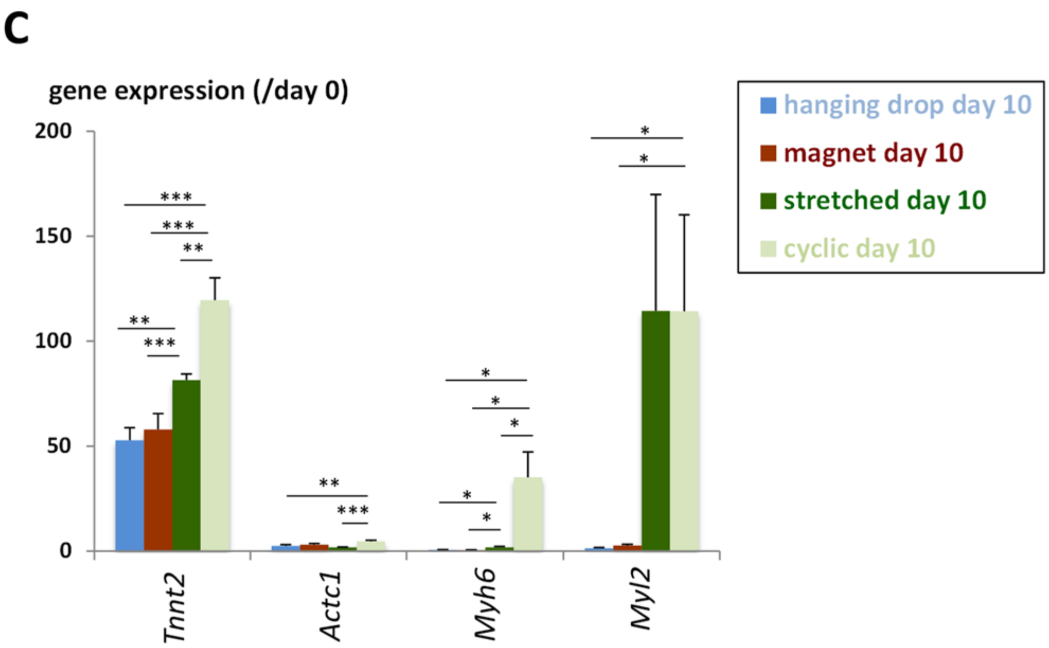

Figure 5C: Gene expression at longer maturation times (day 10) for specific cardiac markers cardiac troponin T (*Tnnt2*), cardiac α-actin (*Actc1*), α myosin heavy chain (*Myh6*) and myosin regulatory light chain 2 (*Myl2*). All EBs were obtained from 10000 ESCs. For the hanging drop formation (blue), ESCs were not labelled with the magnetic nanoparticles. For the three other conditions, ESCs were magnetic (3 pg of iron per cell): EB formation by magnet with no further stimulation (dark red), stretched stimulation (dark green) and cyclic stimulation (light green). mRNA levels are shown relative to control (day 0, defined as 1), and normalized to reference gene RPLP0.

These new results are now commented in the text, Results and Discussion sections:
"Finally, in order to detect if a commitment towards the cardiac lineage was really enhanced, we analyzed EB at longer maturation times (day 10), and quantified by qPCR the expression of transcripts encoding for specific cardiomyocyte markers. We tested troponin T (*Tnnt2*), involved in cardiomyocyte contraction, cardiac α-actin (*Actc1*), the cardiac cytoskeletal marker, α-myosin heavy chain (*Myh6*), involved in contraction and considered as a maturation marker, and myosin regulatory light chain 2 (*Myl2*), involved in the regulation of myosin ATPase activity and known as a ventricular cardiomyocyte marker (Figure 5C).
The hanging drop and magnet conditions of EBs formation led to similar results for all genes and, overall, the efficiency of differentiation towards functional cardiomyocytes was increased upon application of stretched and cyclic stimulations. Compared to the magnet condition, *Tnnt2*, *Myh-6* and *Myl2* genes were overexpressed for the stretched condition, and this

upregulation was higher following cyclic stimulation for *Tnnt2* and *Myh-6*. The impact on cardiac α-actin was less pronounced, with a significant upregulation only for the cyclic condition. This protein is, among others involved in the left ventricular compaction,[37] and probably expressed later."

2. In the attempt to show more evidence towards the cardiac mesoderm differentiation, the authors show Brachyury and NKX2.5 immunostainings (Fig. 4B). Brachyury and Nkx2.5 are both transcription factor with clear nuclear localization, however in the images shown, both proteins look like membrane localised. Do the authors have any explanation for this?

We agree with the reviewer. Furthermore, as brachyury is already at very low levels at day 5 (maximum at day 3, see also the following answer to question 5), we have deleted brachyury immunostainings, to avoid some misinterpretation.

3. The authors conclude the manuscript stating that 'The magnetic stretcher can be used to virtually form 3D model tissues from any cells, magnetically and in situ, and then stimulate them at will, opening windows not only for biophysical studies, but also for tissue engineering'. While it is true that the system developed by the authors (re)opens the possibility of magnetically stimulate mESCs during differentiation, how this could be applied to tissue engineering is very obscure. As the authors show themselves, as soon as they remove the magnetic force applied, the EB recover the original shape. In order to do 3D cell engineering, it is a must that cells keep the shape within the 3D structure.

The reviewer is totally right to stipulate that regenerative medicine application is not the scope of this manuscript, focused on EB stimulation, and its role on the ESC differentiation profile. When we wrote that the system would have some potential for tissue engineering, we had in mind applications such as cell alignment (i.e. muscle cells) in a 3D cellular construct, for muscle tissue engineering. That said, we have removed the sentences opening up the device to tissue engineering as such applications are still too far-fetching.
However, we emphasize that the tissue indeed keeps itsshape after removal from the stretcher. This can be clearly seen in new Figure 4E, where we imaged cryosections in the parallel and perpendicular directions of the tissue.

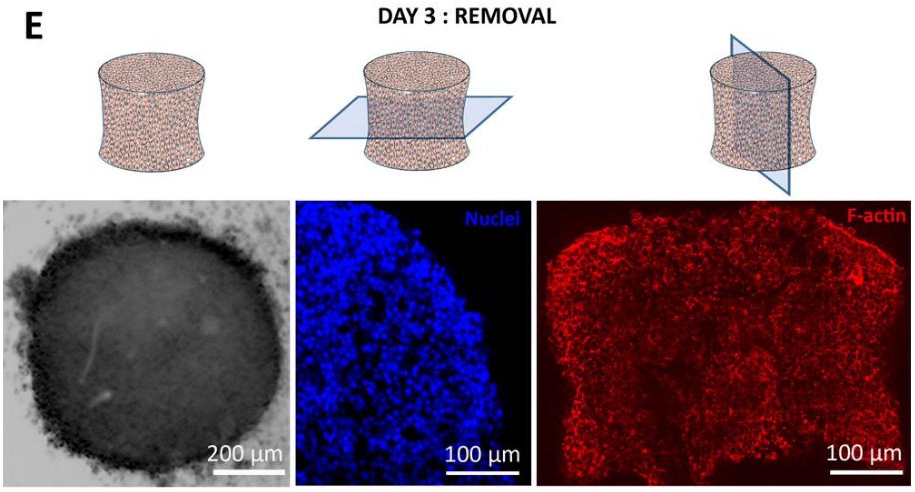

Figure 4E: Fluorescence imagee (DAPI staining, middle; F-actin staining, right) of 16-µm cryosections in the perpendicular (middle) and parallel (right) direction of the tissue axis. The

nuclei image shows a homogeneous cell density in the center of the EB, while F-actin is homogenous whatever the localization of the cell inside the stretched EB. All EBs were formed with 10 000 ESCs.

4. Something that seems contradictory in the discussion and the authors do not comment on it: according to the paper cited *3*, the force required to separate 2 cells is several hundred nN, and the force the authors use to deform the EB is in the order of µN. How do the authors explain that cells in their EB only deform but do not separate from one another?

We apologize for this misunderstanding. Indeed the force required to separate 2 cells in several hundred nN. By contrast, the magnetic force created here on a single cell is 100 pN, as said in the manuscript in another section. We should have reminded it in the section describing the global tissular force, which is in the order of µN because it is the additive action of all cellular forces generated on the 10000 cells embedded within the EB. This discussion on the magnitude of forces applied here is very important, especially to emphasize that the stimulation is a global strain applied to the tissue, and that forces on single EBs are too small to impact the cell morphology or cell-cell adhesions.
To further assess this phenomenon, we have added some live experiments of cyclic stretching (new Figure 4D). It demonstrates that the strain rate distribution within the EB, determined by a texture correlation algorithm (PIV analysis), is uniform.

This is now discussed as follows:
"First, the magnitude of the magnetic forces applied to single ESC within the magnetic embryoid body needs to be discussed. One should then compare their intensities (in the 0.1 nN range) to the one of cell-cell adhesion. mESC-mESC adhesive forces (generated by E-cadherin/E-cadherin bonds, measured at 73 pN each) is about 9.1×10^5 pN.[47] This is of the same order as the force required to separate two cells (several hundred nN).[48] Thus, while the magnetic force is sufficient to attract cells and aggregate them, it is not enough to break out cell-cell bonds within an EB. This would explain why the attraction exerted by the magnetic microtip does not affect ESC differentiation: the cellular magnetic forces are far weaker than the intercellular cohesive forces, and no mechanosensitive pathways are activated by the cellular magnetic forces. Therefore it is quite difficult to compare our experiment with the mechanical stimulations and mechanotransduction evidenced in other studies.
By contrast, when we approached the second magnetic microtip towards an EB formed on the first magnetic microtip, the EB was rapidly deformed. The magnetic EB (containing N cells, each carrying a magnetic moment Mcell) must then be considered as a continuous tissue with a global magnetic moment of $N x M_{cell}$. In the case where N = 10 000, the tissue force due to the field gradient created by the second tip is of the order of µN, which is sufficient to deform the EB. One must then consider that the stimulation is similar to a global strain, where the cells reorient and rearrange during the stretching, and move as well together with the tissue during the cyclic stimulation. Cell movements analyzed by PIV (as shown in Figure 4D) demonstrate this vision of a tissue stimulation. Indeed, all the EB are deformed as a whole, and cells are moving altogether and deformed with the same strain, as demonstrated with no singularities in the divergence of the velocity."

5. There are a number of occasions where the authors do try to oversell or interpret results in the way it seems more convenient to them:
a. P. 11 (lines 222-224):'First, we can note an increase in the expression of the 2 genes (T and Nkx2.5) involved in the (cardiac) mesoderm pathway: 1.5-fold and 3-fold for T in the stretched

and cyclic conditions, respectively;' The same fold-change was disregarded in the experiment shown in Fig. 2E.

This suggestion has been considered in the revised version of the manuscript. Because T is expressed only during the very early days of differentiation (1-3), its regulation is not relevant to be accounted for at day 5. We have therefore deleted all comments relative to T in the manuscript.

b. P. 11 (lines 232-234): 'Finally, and logically, the expressions of other genes involved in the endoderm or ectoderm pathways, were either almost unchanged (Lama1, Lamb1, Lamc1, Nes and Pax6)…' the expression of these genes do change and the difference is statistically significant.

We have reformulated these sentences, and highlighted that the expression of these genes is hardly, if not at all, up-regulated compared to undifferentiated ESCs.

Minor comments:

1. P. 3 line 38: There is an extra ').'

Thank you for noticing. It has been removed.

2. LamC1 does show a difference when forming the magnetized EBs, (Fig.1F), what is the explanation for this?

The increase in that case is small (1.4-fold), and given the low level of expression, we believe that, even if statistically significant, this upregulation is not significant (below the "1.5-fold threshold" usually taken to trust qPCR differences).

3. Time frames in Fig 2A are not indicated

The image shown in Figure 2A was obtained by superimposing frames filmed at 0.1 s intervals. It was indicated in the caption:

"The microtip was introduced into a chamber containing suspended cells under a microscope, and cell movements were video-monitored with a 10x objective at 0.1 s intervals".

However, in the next sentence describing the image, it was not specified, and we modified it accordingly:

"Here, 100 movie images were superimposed (0.1 s time intervals) in order to directly observe the trajectories of the cells migrating towards the magnetic microtip."

4. Some features in the figures are too small to be legible.

We tried to make them more legible.

5. Cells in Fig 3B do not appear elongated as claimed.

We agree that previous Figure 3B was poor. We have removed it. Instead, we are now showing fluorescent images on new Figure 4D with PIV analysis. And we have deleted any claim concerning cell elongation.

6. Fig. S3: the scale bar size is not indicated.

Sorry for the omission, size is now indicated in the figure caption.

7. There is no reference indicating the source of the information included in the diagram shown in Fig S9.

Sorry again, and thank you for noticing. The sources are now included.

Reviewers' comments:

Reviewer #1 (Remarks to the Author):

Thank you for revising the manuscript. I appreciate your efforts in addressing my concerns. However, the following points need your clarification:

1. The statement in your rebuttal letter "However, most of them concerned mesenchymal stem cells or endothelial progenitor cell differentiation" is not correct. To name a few, here are some examples of ESC/ pluripotent stem cells work that shows "purely mechanical factors" can indeed drive differentiation:
a. Sun et al., Nature Materials, 2014
b. Keung et al., Intg. Biol., 2012
c. Du et al., PNAS, 2011
d. Uda et al., BBRC, 2011
e. Chowdhury et al., Nature Materials, 2010
f. Adamo et al., Nature, 2009
I am not so sure if the manuscript, in its current form, describes the development of the field and the current status of the field. Therefore, the achievement reported by this manuscript seem a bit of overstatement.

2. Another statement "By contrast, stimulation on 3D constructs was rarely achieved" may not be correct. Stimulation can be of various form. There might be environmental cues present to stimulate endogenous force generation via different pathways. Previously many works have been done on 3D engineered constructs that provide certain ECM cues. Please see original/ review articles from Todd McDevitt lab. Also please refer to a fairly recent article from Ning Wang's lab (Poh et al., Nature Communications, 2014) relevant to ESCs.

3. While comparing 2D single cell analysis vs 3D in vitro model analysis, I think the authors do not quite appreciate how much insight and depth of knowledge single cell analysis on 2D substrates provided over the last decade. It is true that there might be some differences between 2D and 3D conditions. However, your results with 3D constructs do not provide any novel molecular mechanism (e.g. detailed outside-in signaling) or challenge any current understanding of molecular mechanism, therefore your statement is not well justified.
4. This is where I am getting completely lost.
a. Please explain what you meant by "deforming the tissue as a whole, but not deforming single cells." Aren't you deforming the single cells containing the nanoparticles within the aggregate that in turn results in aggregate deformation?
b. Please help me understand "It confirms that the cells do not incur a stress, but a homogeneous strain, transferred from the total strain (10%) imposed by the EB stretching." How can a viscoelastic body such as the cell when subjected to a uniform strain field do not experience a stress?

5. When you say "Thus each cell experiences the same deformation rate" are you measuring strain rate over the entire EB in 3D stacks? If yes, perhaps you should provide evidence. It would be informative to see if there are any singularities or any non-uniformities in strain rate for any part of the EB.

6. You are stating that "no mechanosensitive pathways are activated by the cellular magnetic forces' which you cannot state unless the mechanotransduction pathways are thoroughly investigated in a systematic way. In the discussion when you say 0.1 pN of force applied to single cells induce cardiac mesoderm formation, it's not clear why they make such commitment. What are the key molecules responsible in the differentiation pathway with such low forces? Why aren't they committing to other lineages? In general, the manuscript lacks molecular mechanism as agreed by the authors.

Reviewer #2 (Remarks to the Author):

I am satisfied that the authors have addressed all my concerns and worked hard to incorporate all the reviewers' comments into their new improved manuscript.

Reviewer #3 (Remarks to the Author):

This reviewer is overall satisfied with the authors' responses to most of my comments and acknowledge the substantial improvement to Du et al. manuscript. There are still a misunderstandings which I think is worth stating and commenting on, but I'll leave it to the editor to decide whether it should be addressed by the authors before publication or not.

Comment 3. On a related topic to point 2, the authors do not show what the long term effect of the nanoparticles in the mESCs is. Also the following should be tested: -for how long are the nanoparticles maintained in the cells once they've been endocytosed? If the authors passage the cells, after how many passages do these particles disappear? This is an important issue if the application of this technique will eventually be to do in vivo work. -the cell viability tests shown are performed after 30min or 2-4h after incubating the cells with the nanoparticles, if the incubation is done for 30min and then viability tests are done a few days after, do the magnetised cells show a decreased viability?

Authors' response: 'Concerning the long term effect of the nanoparticles, first, we must say that at days 5 and 7 of EBs maturation, we detected no changes in gene expression for magnetic cell compared to control cells (Figure 1F). Besides, we have now performed new experiments at longer maturation times (day 10), and again, we have not seen differences in gene expression (here for the cardiac markers) for EBs formed with unlabeled cells, or cells having nanoparticles inside (see new Figure 5C). Concerning the long-term fate of nanoparticles within the cells, we have performed a new series of experiments to monitor the magnetism of EB over 7 days, which is a direct indicator of the nanoparticle integrity and presence within the cells over long culture times. It demonstrates that EBs are still magnetic after one week of tissue maturation. This experiment is now shown as a new Figure 2E (copied below). It consists of measuring the EB magnetic migration towards a magnet at different growth times, after the initial incorporation of the magnetic nanoparticles.'

The authors decided to check magnetism of the cells rather than cell viability which was what was clearly asked. In other words: if the authors plate the cells with the nanoparticles and keep them in culture (normal 2D culture with standard medium), do the cells show reduced viability? (do they die due to the presence of the nanoparticles?). And how many passages do the authors need to do before the nanoparticles are excreted (if they are)? The authors only check cell viability after 2 days.

Finally, please do check the numbering of the Sup Figs. As they often do not correspond to the number

Response to reviewer #1

Thank you for revising the manuscript. I appreciate your efforts in addressing my concerns.

Thank you for this appreciation.

However, the following points need your clarification.

We have clarified each point, as detailed below.

1. The statement in your rebuttal letter "However, most of them concerned mesenchymal stem cells or endothelial progenitor cell differentiation" is not correct. To name a few, here are some examples of ESC/ pluripotent stem cells work that shows "purely mechanical factors" can indeed drive differentiation:
a. Sun et al., Nature Materials, 2014
b. Keung et al., Intg. Biol., 2012
c. Du et al., PNAS, 2011
d. Uda et al., BBRC, 2011
e. Chowdhury et al., Nature Materials, 2010
f. Adamo et al., Nature, 2009
I am not so sure if the manuscript, in its current form, describes the development of the field and the current status of the field. Therefore, the achievement reported by this manuscript seem a bit of overstatement.

We understand the reviewer's viewpoint, and we thank her/him for this update. Like the reviewer, we are aware that there exist works assessing the role of mechanical factors in the differentiation of ESC / pluripotent stem cells. In the introduction of the original manuscript, we had emphasized that works on MSCs and EPCs are more numerous than works on ESCs, and cited a selection of corresponding studies. We understand, however, that the phrasing may have been misleading.

To avoid any confusion, we have carefully modified the introduction. In doing so, we now quote the works suggested by the reviewer on pluripotent stem cells in the 2D culture setting, either exploring mechanotransduction pathways (and particularly Oct3/4 expression) on single ESCs by applying mechanical stresses to integrin receptors or focal adhesions (references d,e), or analyzing the hematopoietic response of ESC to fluid shear stress (reference f), or demonstrating the role of microenvironmental cues such as substrate rigidity on hPSCs neuronal differentiation (references a,b).

"During this last decade, a growing number of studies have evidenced that mechanical factors can influence stem cell differentiation[7]. This idea of a physical guidance of differentiation emerged from studies using adult mesenchymal stem cells, and was then tested on pluripotent/embryonic stem cells. Most techniques applied on two-dimensional (2D) cell cultures, focusing on the role of (i) microenvironmental mechanical cues such as substrate rigidity;[8-13] (ii) flow-induced shear stress;[14-16] (iii) strains imposed on cell monolayers by the stretching of deformable supporting membranes;[17-19] or (iv) local forces applied on beads attached to the cell surface.[20, 21] "

7. Discher., et al. *Science* **324**, 1673-1677 (2009).
8. Engler, A.J., et al. *Cell* **126**, 677-689 (2006).
9. Evans, N.D. et al. *Eur Cell Mater* **18**, e13 (2009).
10. Gobaa, S., et al. *Integrative Biology* **7**, 1135-1142 (2015).

11. Sun, Y. et al. *Nature materials* **13**, 599-604 (2014).
12. Keung, A.J., et al. *Integrative Biology* **4**, 1049-1058 (2012).
13. Przybyla, et al. *Cell Stem Cell* **19**, 462-475 (2016).
14. Huang, Y. et al. *PloS one* **7**, e34960 (2012).
15. Adamo, L. et al. *Nature* **459**, 1131-1135 (2009).
16. Yamamoto, K. et al *Am. J. Physiol. Heart Circ. Physiol.* **288(4)**: H1915-1924 (2005)
17. Geuss, L.R. & Suggs, L.J. *Biotechnology progress* **29**, 1089-1096 (2013).
18. Gwak, S.-J. et al. *Biomaterials* **29**, 844-856 (2008).
19. Kurpinski, K., et al. *Proceedings of the National Academy of Sciences* **103**, 16095-16100 (2006).
20. Chowdhury, F. et al. *Nature materials* **9**, 82-88 (2010).
21. Uda, Y. et al. *Biochemical and biophysical research communications* **415**, 396-400 (2011).

All these works were performed in the 2D culture setting, on monolayers, at the single cell scale. The novelty of the present engineering approach is to provide a way to probe physical sensing in the 3D setting, without the need for a scaffold. This is further discussed in the next question.

2. Another statement "By contrast, stimulation on 3D constructs was rarely achieved" may not be correct. Stimulation can be of various form. There might be environmental cues present to stimulate endogenous force generation via different pathways. Previously many works have been done on 3D engineered constructs that provide certain ECM cues. Please see original/ review articles from Todd McDevitt lab. Also please refer to a fairly recent article from Ning Wang's lab (Poh et al., Nature Communications, 2014) relevant to ESCs.

We agree with the reviewer. However, strain (or stress) has rarely been imposed on purely cellular 3D engineered tissues. Most existing stimulations have been driven by modifying environmental cues such as extracellular matrix components or supporting gels stiffness. Again, we acknowledge that the phrasing has been misleading, and that stimulations provided by ECM cues must be clearly presented.

In this context, we agree that works from McDevitt lab produced new insights in stem cells differentiation in 3D gels with precise control of the biophysical and biochemical environment and of the spatial patterning of the aggregates. Similarly, works such as Poh et al brilliantly demonstrated that cell–matrix and cell–cell interactions can organize germ layers spatially after ESC lineage-specific differentiation. We have modified the introduction accordingly.

"Multicellular tri-dimensional (3D) approaches have also recently received an increasing interest for studying stem cell behavior beyond the classical 2D culture conditions. Two main strategies are used. First, scaffold-based constructions not only allow to stimulate mechanically the seeded stem cells,[22, 23] but also provide precise 3D control of extracellular matrix cues.[24, 25] Second, scaffold-free magnetic or printing technologies make it possible to control spatial patterning of aggregates[26] or to create multilayer structures.[27] To the best of our knowledge, the possibility to form EBs from ESCs and further apply to them a controlled strain (or stress) in situ, in the 3D geometry, and without the need for a supporting matrix, has not yet been reported."

20. Pelaez, D., et al. *Stem cells and development* **18**, 93-102 (2009).
21. Henstock, J. & El Haj, A. *Regenerative medicine* **10**, 377-380 (2015).
22. Matthys, O.B., Hookway, T.A. & McDevitt, T.C. *Current stem cell reports* **2**, 43-51 (2016).
23. Poh, Y.-C. et al. *Nature Communications* **5** (2014).

24. Bratt-Leal et al. *Int. Biol.* **3**, 1224-1232 (2011).
25. Mironov V. et al *Biomaterials* **30** 2164-2174 (2009)

3. While comparing 2D single cell analysis vs 3D in vitro model analysis, I think the authors do not quite appreciate how much insight and depth of knowledge single cell analysis on 2D substrates provided over the last decade. It is true that there might be some differences between 2D and 3D conditions. However, your results with 3D constructs do not provide any novel molecular mechanism (e.g. detailed outside-in signaling) or challenge any current understanding of molecular mechanism, therefore your statement is not well justified.

We agree that we do not provide molecular mechanisms. Here we have adopted an engineering approach to propose new tools to stimulate stem cells, in the 3D embryoid body setting, without the need for a scaffold. Our achievement is to modulate the differentiation pathway, in this purely 3D cellular configuration. Forming and deforming an embryoid body in a cyclic manner without any layout has not yet been achieved, and we are thus providing an original magnetic methodology to perform it. We did not explore the molecular mechanism involved. We refer to 2D works, which, as pointed out by the reviewer, have already provided some deep knowledge.
We have now emphasized this point in the discussion section, where we discuss other studies providing molecular mechanisms on mechanical cyclic stretching of ESCs on 2D substrates.

"It is important to emphasize that, herein, we have adopted an engineering approach to offer magnetic tools allowing to form and deform an EB in a cyclic manner without the need for a supporting scaffold. This approach demonstrated that it is possible to enhance cardiac mesoderm differentiation by a mechanical 3D stimulation. The underlying molecular mechanisms involved are still unclear; however, because the mechanical stimulation is that of a global strain applied to the EB, and resembles the situation of a mechanical stretching on a 2D deformable substrate, there are some clues regarding the mechanisms that underly cardiac differentiation.[1] Some studies demonstrated that the production of intracellular reactive oxygen species initiates a cardiovascular differentiation program via induction of several signaling pathways such as PI3K/Akt, ERK1/2, JNK, and p38.[2, 3] In addition, integrin-mediated modification of cell signaling pathways such as PI3K/Akt and GSK-3ß has a particular role in cardiac differentiation.[3, 4] Yet, it is known that mechanical stimuli such as stretch and shear stress can activate these signaling pathways.[5-7] Moreover, Banerjee et al[8] demonstrated that mechanical strain is sufficient to regulate Tgf-β signaling and Tgf-β-dependent gene expression. Toh and Voldman demonstrated that mouse ESCs also mechanically sense shear stress via heparin sulfate proteoglycans to modulate Fgf5 expression.[9] Finally, cell stretching also triggers modulation of the cell membrane and orientation of actin filaments, which facilitates cell-cell connections required for intercellular communication.[10] Cell-cell contacts through cadherin adhesion[11, 12] enhance this process by alpha-catenin and vinculin recruitment and are involved in regulation of the Oct4-Nanog-Sox2 circuitry.[13] "

4. This is where I am getting completely lost.
a. Please explain what you meant by "deforming the tissue as a whole, but not deforming single cells." Aren't you deforming the single cells containing the nanoparticles within the aggregate that in turn results in aggregate deformation?

Here we understand that we did not appropriately describe the set-up and the forces, and we are very grateful to the reviewer for pointing it out.

We have added a few paragraphs to do so (copied below in blue lettering), as well as a new figure (Scheme 2, also copied below). The main argument goes as follows:

The magnetic forces exerted by the magnet on the individual cells are strongest near the magnetic microtips (see field gradient map in Figure 2B). That, combined with the fact that these forces accumulate as cells push onto each other, implies that most of the stress is concentrated in the "proximal region" (cells located near the glass walls) which is squeezed between the magnetic forces and the glass wall reaction force. The magnetic forces and the wall reaction force thus play the role of a clamp. By contrast, within the majority of the sample (intermediate region, outside both clamps), the strain (and, correspondingly, the stress) is determined mainly by the relative motion of both clamps and only marginally by the stress within each clamp. As a result, the strain is expected to be rather uniform. To check that, we performed PIV analysis (with cells used as tracers) and were able to show that the stretching rate is uniform over the whole aggregate.

In short, what we meant is: the magnetic field does not deform single cells (except within each clamp) and the apparatus deforms the tissue as a whole (uniformly except within the clamps).

b. Please help me understand "It confirms that the cells do not incur a stress, but a homogeneous strain, transferred from the total strain (10%) imposed by the EB stretching." How can a viscoelastic body such as the cell when subjected to a uniform strain field do not experience a stress?

Our sentence was indeed awkward. What we meant was: The magnetic forces are mainly concentrated within both "clamps", and are negligible in the remaining part of the sample. As a result, the stress is uniform in the EB (except in the clamps). When we were clumsily saying "the cells do not incur a stress", we meant to refer to most cells in the EB and only to the magnetic contribution to the stress.

Of course, the uniform stress in the region between both clamps directly implies a uniform strain.

The sentence under discussion has now been removed, and, as mentioned above, a whole description of the stresses in the EB has been introduced (pages 16-17), as copied below, with reformulation of some of the previous statements, and with the introduction of new concepts.

"In order to fully understand the formation and stimulation of the EB from a mechanical point of view, let us now examine the corresponding force balance in the magnetic stretcher apparatus, as depicted in Scheme 2.

During EB formation (part A1), the magnetic microtip subjects each cell to a magnetic force (blue arrow), pulling it against its neighbors, and thus contributing to squeezing all cells, but more strongly the ones closer to the microtip. The total resulting cellular magnetic force is then transmitted to the glass wall above the microtip, and is exactly balanced by the wall reaction force (green arrow). Straight after magnetic cell assembling, cohesion builds up (part A2) through cell-cell junctions, and the whole assembly displays enough cohesion to be used as a standalone EB (part A3) and sustain stretching. Magnetic stretching is initiated by approaching a second magnetic microtip. The upper cell layers are then pulled against the upper wall (parts B1-B3). At each wall, the magnetic stretcher thus plays the role of a "clamp" acting on a "proximal region" of the sample held by the opposing magnetic force and the wall reaction force. At first, for a small distance between the microtips (part B1), the intermediate part of the EB is at rest while within each "clamp", the wall reaction force exactly balances the corresponding total magnetic attraction force. Moving the "clamps" apart stretches the intermediate part of the EB (parts B2-B3) and exerts pulling forces (black arrows) on the upper and lower (thin) proximal regions. Modulating the distance between the walls (parts B1-B3) affects the degree of stretching of the large intermediate part (in a uniform manner as shown by the PIV measurements depicted in Figure 4D) and the corresponding pulling forces and wall reaction

forces. Meanwhile, the magnetic forces remain unchanged. The "stretched" and "cyclic" conditions for the aggregate correspond to a stretched situation like B2 and to cycling between situations B2 and B3, respectively.

Let us now discuss whether the magnetic forces used to manipulate the magnetic EB can endanger the EB cohesion that results from cell-cell adhesion forces. Adhesive forces are generated by E-cadherin/E-cadherin bonds, measured at 73 pN each, which amounts to about 900 nN per mESC-mESC pair.[55] One should compare this intensity to the highest tensile cell-cell forces within the aggregate. This happens to be within the intermediate region in the stretched configuration (see Scheme 2, part B3). The magnitude of the tensile force in this region is at most equal to that of the total magnetic force, around 1000 nN. This tensile force is distributed over all cells within a horizontal section of the aggregate (for instance the mid-height plane), corresponding to roughly 500 cells. It yields a typical maximum tensile force of 2nN per cell pair, safely below the mESC-mESC separation force.

Finally, it is important to emphasize that, using only one magnetic microtip does not alter significantly the gene expression (Figure 3B), while using two magnets, whether in the "stretching" (static) or "cyclic" condition, clearly upregulate some genes (Figures 5A and 5C). With just one magnetic microtip (Scheme 2, part A2), the tip behaves as a "clamp" holding a limited ("proximal") region of the EB, the only region where substantial forces are present. Indeed, the applied magnetic forces and resulting compression strongly decay with distance from the tip. The major part of the EB thus undergoes negligible stress in this one-magnet situation, and overall gene expression is not affected. By contrast, in the two-magnet situation, the major part of the EB is stretched (Scheme 2, parts B2 and B3). As a result, gene expression is expected to be altered in most cells, as detected with global PCR measurement."

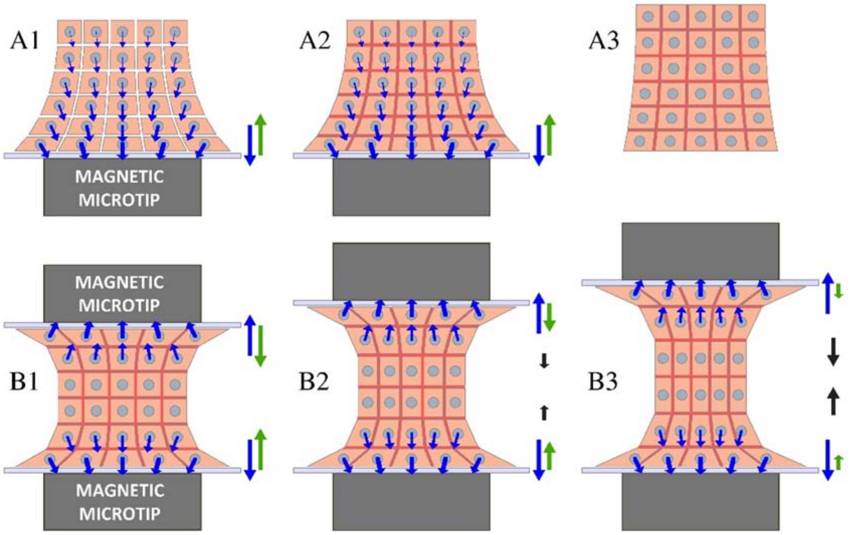

Scheme 2: Schematic view of the forces involved within the EB in the magnetic stretcher.
(A1) Formation of the EB on the magnetic microtip located below a glass wall. Each cell is subjected to a magnetic force (blue arrow). The total resulting magnetic force (shown on the right-hand side, also in blue) is exactly balanced by the wall reaction force (green arrow). This pair of forces act like a "clamp" that holds mainly the "proximal" region of the sample, closest to the glass wall.
(A2-A3) Adhesion molecules (in red) develop the EB cohesion, without affecting forces, and the whole aggregate can be used as a standalone EB. (B1-B3) When another magnetic microtip is approached with another glass wall, the upper cell layers are "clamped" against the upper wall in a similar way as in (A1-A2). Varying the separation of both "clamps" makes it possible to adjust or cycle

the (tensile) strain of the main part of the EB (represented here with a thickness of only two cells for simplicity, but actually corresponding to the major part of the entire EB).

5. When you say “Thus each cell experiences the same deformation rate” are you measuring strain rate over the entire EB in 3D stacks? If yes, perhaps you should provide evidence. It would be informative to see if there are any singularities or any non-uniformities in strain rate for any part of the EB.

Our conclusions are based on two majors points:

- First, the observation of the whole aggregates at the end of the stimulation does not show singularities;
- Second, using cells as tracers during the cyclic stimulation of the cylindrical EB, we performed particle image velocimetry (PIV) over one entire side view of the aggregate (note that light diffusion hinders observation of the aggregate bulk). We thus obtained a measurement of the 2D-projected velocity field of the cylinder edge and derived the corresponding 2D strain rate, using the technique already published in experiments performed on muscle cells included in polymer matrix (Zhao et al Advanced Mat. 2013). We observed that the 2D strain rate was uniform over the whole field of view. Since the aggregate is axisymmetric, the strain rate can be considered uniform over the entire sample.

In the sentence “Thus each cell experiences the same deformation rate”, we just wanted to emphasize that we focus on the engineering strain applied to the EB and not on the stress.

6. You are stating that “no mechanosensitive pathways are activated by the cellular magnetic forces’ which you cannot state unless the mechanotransduction pathways are thoroughly investigated in a systematic way. In the discussion when you say 0.1 pN of force applied to single cells induce cardiac mesoderm formation, it's not clear why they make such commitment. What are the key molecules responsible in the differentiation pathway with such low forces? Why aren't they committing to other lineages? In general, the manuscript lacks molecular mechanism as agreed by the authors.

Here we truly apologize for the mistake that we indeed detected on page 18 “It is finally important to emphasize that, herein, considering the low (0.1 pN per cell)”. This was really a very confusing typing mistake, and we are grateful to the reviewer for noticing it. The correct value is 0.1 nN, as it was indicated correctly 2 pages above, on page 16 (“First, the magnitude of the magnetic force applied to single ESC within the magnetic embryoid body needs to be discussed. One should then compare its intensity (in the 0.1 nN range) to the one of cell-cell adhesion”) as well as in the Results section page 8 (“The field gradient is 500 T/m at 1 mm from the surface of the microtip (1000 T/m at 0.4 mm), equivalent to a force of about 100 pN (200 pN, respectively) on an ESC containing 3 pg of iron.”) and on page 12 (“At 400 µm from the magnetic tip/attractor, the magnetic gradient of about 1000 T/m provides a force per single ESC (loaded with 3 pg of iron, or equivalently a magnetic moment of $2x10^{-13}$ A.m²) of 200 pN approximately.”).

The magnetic force range is thus in the 0.1 nN range.

Anyway, such a force is still small. It is true that we cannot rule out that it could have an impact without investigating the mechanotransduction pathway. It is unlikely, but anyway, we removed this comment. By contrast, we believe that we are now explaining in a more understandable way how the mechanical stimulation is applied onto cells within the embryoid body. It is a global stretching,

and it is the stretching forces that are responsible for the commitment. That is why we said that it was not the magnetic forces *per se*.

This is also why we are comparing our 3D deformation to 2D stretching, and discuss the mechanisms involved in this 2D cyclic stretching towards cardiac mesoderm differentiation. Our goal here is to provide an engineering method to form and stimulate embryoid bodies *in situ* without the need for a scaffold, and to determine the impact of the stimulation on the EBs differentiation profile. We now clearly describe this goal at the beginning of the discussion, and we acknowledge that we do not provide here a molecular mechanism:

"The main (successful) objective of this work is to provide a method for assembling embryonic stem cells into 3D embryonic bodies without the need for a scaffold and further stimulating mechanically this embryoid body *in situ*, with the overriding aim to determine whether embryonic stem cell differentiation could be enhanced in this 3D setting through mechanical stimulation. The corresponding detailed molecular mechanisms involved are beyond the scope of the present study, but a brief review of potential mechanisms is provided".

Response to reviewer #2

I am satisfied that the authors have addressed all my concerns and worked hard to incorporate all the reviewers' comments into their new improved manuscript.

We are grateful to the reviewer for all previous comments helping in improving the manuscript.

Response to reviewer #3

This reviewer is overall satisfied with the authors' responses to most of my comments and acknowledge the substantial improvement to Du *et al.* manuscript.
We thank the reviewer for this positive evaluation of our revision, and we thank her/him again for all the suggestions initially made that significantly improved our work.

There are still a misunderstandings which I think is worth stating and commenting on, but I'll leave it to the editor to decide whether it should be addressed by the authors before publication or not. The authors decided to check magnetism of the cells rather than cell viability which was what was clearly asked. In other words: if the authors plate the cells with the nanoparticles and keep them in culture (normal 2D culture with standard medium), do the cells show reduced viability? (do they die due to the presence of the nanoparticles?). And how many passages do the authors need to do before the nanoparticles are excreted (if they are)? The authors only check cell viability after 2 days.

In order to answer the initial reviewer's concern, we did check viability after labeling, in a normal 2D culture with standard medium. But we compared magnetic cells viability to control cells only 2 days after labeling. To fully answer the reviewer, we are now presenting additional experiments in the new supplementary figure S3, part A, to check viability over a longer period of time, i.e., up to 9 days after labeling. The new figure is copied below, and clearly shows no impact of the magnetic nanoparticles over long culture periods. This is now indicated in the manuscript: "Cell viability was also examined on the long-term, i.e., over 9 days after labeling for the 30-min incubation condition (3pg per cell), and no impact was observed on the cells' viability and ability to replicate compared to control cells (supplementary Figure S3)."

Concerning the second part of the question, i.e., the presence of the nanoparticles after maintaining the cells in culture over several days, we initially made the choice to monitor the magnetism (and thus the presence of the nanoparticles) in the embryoid body setting, because it is the configuration we systematically used in our study, straight after the nanoparticle incorporation. However, we agree that it is also interesting to monitor the magnetism of single cells kept in 2D culture after labeling. We have performed this measurement by single-cell magnetophoresis, up to 4 days after labeling. As expected, over the course of cell division (about one per day), each cell shares its magnetic content in between its two daughter cells, resulting in a half-fold decrease of the mass of iron per cell. This also demonstrates that no massive expulsion of nanoparticles occurs after their intracellular incorporation.

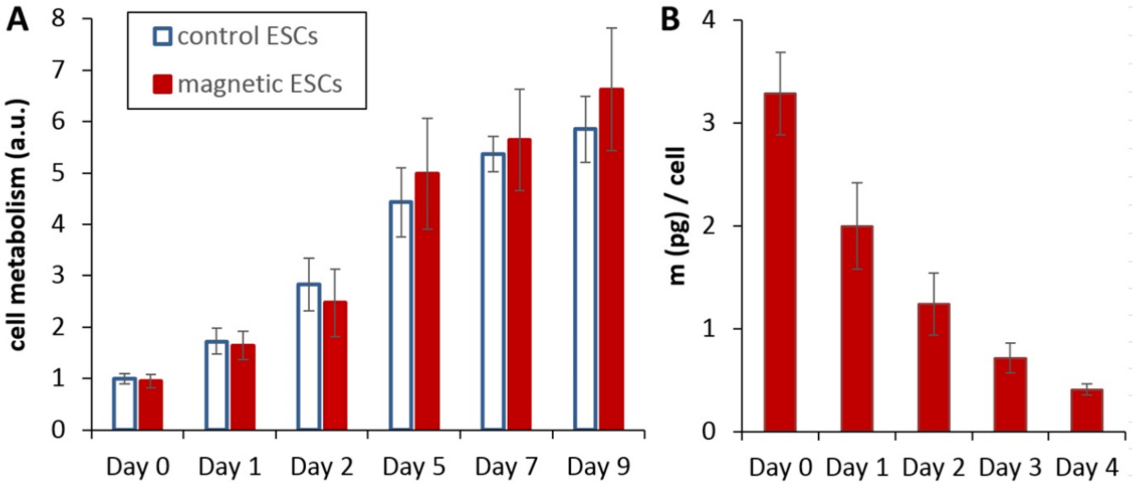

Figure S3: **A**. Cell metabolism measured by Alamar Blue assay on standard 2D ESCs culture at different times after incubation with magnetic nanoparticles (cellular uptake of 3.2±0.2 pg of iron per cell): first day (day 0, two hours after incubation); 24 hours later (day 1); 48 hours later (day 2); and then 5, 7 and 9 days later. The data show that cell viability is not impacted by the intracellular presence of the nanoparticles over long-term periods. **B**. Intracellular mass of iron (expressed in pg per cell) measured each day by single-cell magnetophoresis, during the 4 days following the initial nanoparticle incorporation (day 0). The cellular iron decrease follows the same trend as the cell proliferation on part A, demonstrating that each cell shares its content between the daughter cells during division. One can however note that the decrease in cellular iron mass is slightly more marked than the increase in cell numbers, revealing a degradation of the nanoparticles inside the endosomes (as quantified in Figure 2E), more likely than a nanoparticle expulsion, which we never observed under normal culture conditions.

Finally, please do check the numbering of the Sup Figs. As they often do not correspond to the number

Thank you for noticing. We carefully proofread the manuscript, and we detected one wrong supplementary figure number (supplementary Figure S8 instead of S11, on page 13), which we replaced.

REVIEWERS' COMMENTS:

Reviewer #1 (Remarks to the Author):

I am satisfied with the current revision and thank the authors for taking the time to address my concerns.

Reviewer #3 (Remarks to the Author):

I am satisfied that the authors have addressed all my concerns and worked hard to incorporate all the reviewers' comments into their new manuscript.
